# Effects of high-intensity training on the quality of life of cancer patients and survivors: a systematic review with meta-analysis

**DOI:** 10.1038/s41598-021-94476-y

**Published:** 2021-07-23

**Authors:** Ana Myriam Lavín-Pérez, Daniel Collado-Mateo, Xián Mayo, Gary Liguori, Liam Humphreys, Robert James Copeland, Alfonso Jiménez

**Affiliations:** 1 PhD International School, Program of Epidemiology and Public Health (Interuniversity), Rey Juan Carlos University, 28933 Móstoles, Spain; 2 Centre for Sport Studies, Rey Juan Carlos University, 28943 Fuenlabrada, Spain; 3 GO fitLAB, Ingesport, 28003 Madrid, Spain; 4 University of Rhode Island, Kingston, 02881 USA; 5 Advanced Wellbeing Research Centre, College of Health, Wellbeing and Life Sciences, Sheffield Hallam University, Sheffield, S9 3TU UK

**Keywords:** Cancer, Health care

## Abstract

Cancer and associated medical treatments affect patients' health-related quality of life (HRQoL) by decreasing functional dimensions of physical, social, cognitive, and emotional well-being, while increasing short and late-term symptoms. Exercise, however, is demonstrated to be a useful therapy to improve cancer patients' and survivors’ HRQoL, yet the effectiveness of high-intensity training (HIT) exercise is uncertain. This systematic review and meta-analysis aimed to analyse the effects of HIT on HRQoL dimensions in cancer patients and survivors as well as evaluate the optimal prescription of HIT. The search followed the Preferred Reporting Items for Systematic Reviews and Meta-Analyses guidelines (PRISMA) and examined Web of Science and PubMed (Medline) databases. Data were analysed utilizing Review Manager Software. Twenty-two articles were included in the systematic review and 17 in the meta-analysis. Results showed HIT improved global quality of life, physical functioning, role functioning, social functioning, cognitive functioning, fatigue, pain, dyspnea, and insomnia, compared to an inactive control group, yet no differences were found between HIT and low to moderate-intensity exercise interventions. Particular improvements in HRQoL were observed during cancer treatment and with a training duration of more than eight weeks, a frequency of 2 days/week, and a volume of at least 120 min/week, including 15 min or more of HIT. Our findings whilst encouraging, highlight the infancy of the extant evidence base for the role of HIT in the HRQoL of cancer patients and survivors.

## Introduction

Cancer survivorship continues to increase, with the latest data indicating an estimated 16.9 million people have survived cancer in the United States. This figure is projected to reach more than 26 million by 2040^1^. Moreover, by 2040, 73% of cancer survivors will be at least 65 years old, suggesting a higher comorbidity burden^1^. Cancer and associated therapies can have severe consequences, including treatment-related side effects that decrease health-related quality of life (HRQoL). HRQoL represents the perception of an individual’s current physical, social, emotional, and cognitive health (functional dimensions), together with individual wellbeing and the cancer symptoms suffered^2^. HRQoL is an important variable to consider when making clinical decisions^2^, and HRQoL correlates with patients' cardiorespiratory fitness^3^ and cancer-specific mortality^4^ in different types of cancer such as breast^5–7^, lung^8^, colon^9^, prostate^10^.

Short- and long-term^11^ effects of cancer treatments have been shown to compromise patients’ HRQoL. Short-term effects include symptoms of fatigue^12^, weight loss^13^, weight gain^14^, sarcopenia and cachexia^15^, nausea/vomiting^16^, pain^17^, hair loss^18^, dyspnea^19^, insomnia (sleep disturbance)^20^, constipation^21^, and drowsiness^22^. Symptoms such as diarrhea, appetite loss, sore mouth, and sweating are also reported^23^. Late effects of chemotherapy and radiation therapy most commonly include secondary cancers^24^ and cardiovascular disease^25^. Short-term and late effects vary depending on a patients’ medical history and treatment exposures^11^, and can directly impact a survivors physical and mental health, which can worsen with the increased comorbidities that likely occur with aging^26^. Thus, cancer patients’ HRQoL functional capacities, which include physical, emotional, cognitive, social, and mental components, may be negatively affected during and after treatment, and this negative experience may last throughout survivorship^27^.

In addition to pharmacological therapies, numerous interventions (e.g. psychological therapies, meditation, alternative medicines) are available that aim to reduce the effects of cancer, including treatment-related side effects of cancer drugs^28^. The role of exercise as a cancer therapy appears promising given its potential impact on variables such as physical and mental health, cancer symptoms, and clinical components^29^. Exercise programs are known to help improve the management of treatment side-effects, improve functional outcomes^30^, enhance global quality of life, and help manage fatigue^31^. Several meta-analyses and systematic reviews have investigated the role of exercise in the HRQoL of cancer survivors, trying to approximate the best dose–response and the critical exercise loading characteristics of frequency, intensity, type (FITT). Sweegers et al. (2018) and Buffat et al. (2017) concluded that supervised exercise programmes can improve HRQoL and physical function^32,33^ and are more beneficial than unsupervised interventions. Furthermore, in Hong et al. (2019) meta-analysis, positive social functioning effects were found as a result of exercise, with the largest benefits seen when exercise sessions were 45 to 90 min in duration^34^. Moreover, physical cancer symptoms of fatigue, pain, insomnia, and dyspnoea also showed a decrease with exercise programmes^35^.

Low-intensity exercise has been shown to improve depression, anxiety, and overall physical functioning^36^. Moderate-to-vigorous exercise has demonstrated improvements in physical function and reductions in cancer-related consequences^30^. High Intensity Training (HIT) has yielded positive effects^37^ on cardiorespiratory fitness^38^, strength^39^, and body composition^40^, as well as reduced tumor growth^41^. Although the use of HIT as part of cancer-related therapy is increasing, its benefits on HRQoL are unclear. Toohey et al. (2017) systematically reported a higher effect on HRQoL in patients using HIT. Whereas Mugele et al. (2019) stated that HIT did not improve global health status, pain, fatigue, or insomnia. Adams’ et al. (2018) found improvements in cancer-related fatigue and self-esteem when assessed by the Functional Assessment of Cancer Therapy-Fatigue questionnaire. These reviews point out the need for further investigation to clarify the possible beneficial effects of HIT in cancer patients and survivors. Therefore, the primary aim of this study was to explore the effect of HIT on HRQoL dimensions in cancer patients and survivors. Second, we aimed to evaluate the characteristics of HIT for each HRQoL dimension with regard to the intervention timing related to the cancer treatment, mode of exercise, and dose (i.e. duration and frequency).

## Methods

The methodology of the current systematic review was carried out according to the PRISMA (Preferred Reporting Items for Systematic reviews and Meta-Analyses) guidelines^42^. The systematic review was registered with the International Prospective Register of systematic reviews (PROSPERO), identification number CRD42020167203. In this manuscript, we reported the effects on each HRQoL dimension, which includes more than 100 meta-analyses that have been summarized in tables and in the supplementary data. For readers interested in the effects of HIT on cardiorespiratory fitness outcomes in cancer, these have been reported elsewhere^43^.

### Data sources and searches

PubMed (MEDLINE) and Web of Sciences (which includes articles indexed in the KCI-Korean Journal Database, MEDLINE, Russian Science Citation Index, and SciELO Citation Index) databases were used for article searches. The boolean operators employed were (cancer or “neoplasm”) and (HIIT or “high intensity”) and (“quality of life” or “hrqol” or “qol”), limiting the results to articles published in the last 10 years and written in English or Spanish. The search was done from November 2019 to March 2020. The search for published studies was independently performed by two authors (A.M.L-P and D.C-M.), and disagreements were resolved through discussion.

The inclusion criteria established to select the articles were: (a) studies involving any kind of cancer patients, (b) interventions with any kind of high-intensity exercise, (c) articles with any HRQoL outcome registered, and (d) investigations including at least one other group to compare the effects of HIT. Additionally, interventions were excluded in cases of being a letter to the editor, a consensus or guideline, a study protocol or study design, a case report, a follow-up study, meta-analysis, or systematic review. The current review considered high-intensity training as any program (cardiovascular and/or resistance exercise) whose authors classified it as “high-intensity”, including both high intensity interval training and high intensity training.

### Risk of bias assessment

The analysis of the risk of bias was done using the PEDro scale. The scale is known as a valid and reliable instrument to assess eligibility, allocation to groups, blinding of allocation, and comparison between groups at baseline and its outcomes^44^. The leading reason for its selection is due to it being the most used in the Sport Sciences for Health scientific area^45^.

### Data extraction

The main data of participants, intervention, comparisons, results, and study design (PICOS) of each group included in the articles were reported according to the PRISMA methodology^42^. Regarding participants, studies sample size, patients age (mean and standard deviation) and body mass index (BMI), type of cancer, stage, cancer treatment, and exercise intervention timing concerning the therapy phase were reported. The intervention characteristics registered were: program length (in weeks), duration of sessions, weekly frequency, a description of the exercise, its corresponding intensity (control and progression), and adherence data. HRQoL was the outcome reported in this review. The questionnaires used in the different studies were the European Organisation for Research and Treatment of Cancer quality of life Questionnaire C30 (EORTC QLQ-C30), the Functional Assessment of Cancer Therapy (FACT), and the Short-form 36 (SF-36). Results regarding the questionnaires used are in the meta-analysis figures or the supplementary data tables. Of all the surveys, the EORTC QLQ-C30 questionnaire was the most commonly reported and the one with more specific variables analyzed^46^. To make a representative analysis, the EORTC QLQ-C30 dimensions were used to group FACT and SF-36 results. We examined all the questionnaire’s dimensions and related items to establish similarities between the categories. We did this even if there were named differences, but their items evaluated the same topic. Item categorization was analyzed by two of the researchers (AMLP and DCM) who discussed similarities and differences to classify them in useful variables for the meta-analysis. The variables were divided into categories consistent with EORTC QLQ.C30 dimensions, distinguishing global health, functional scale, and symptoms scale. The data from those items that did not correspond to any variable group created, or was registered by less than three articles, was not included in the literature part.

### Statistical analysis

Post-intervention means and standard deviations were extracted from the articles and analyzed using Review Manager Software (RevMan, 5.3)^47^ based on; High-intensity exercise group (HIEG), low-to-moderate exercise group (LMEG), and inactive control group (CG). When outcomes were evaluated on scales with opposite directions, (e.g. pain or fatigue), one of the results directions was multiplied by − 1^48^. The results were reported using standardized mean differences (SMDs) and interpreted according to the Cochrane Handbook^48^ i.e. small effects with scores < 0.4, moderate effects from 0.4 to 0.7, and large effects with > 0.7. The statistical method employed was inverse variance with random effects^49^ and the interval confidence (CI) utilized was 95%.

Different analyses were computed for each dimension (Global health, Physical functioning, Role functioning/physical role, Emotional functioning/wellbeing and mental health, Cognitive functioning, Social functioning, Fatigue/vitality, Nausea, Body pain, Dyspnoea, Constipation, Insomnia, Diarrhoea and Appetite loss). The described procedure was carried out, first, to analyze the difference according to the type of intervention group: LMEG or CG. Secondary calculations were performed contrasting HIEG and CG outcomes with more detail making the following subgroups analysis: (1) interventions conducted before, during, or after cancer treatment, (2) interventions of ≤ 8 weeks or > 8 weeks, (3) only aerobic exercise programs or work-outs with any resistance component, (4) studies where participants exercised ≤ 2 times per week or those > 3 times per week (5) interventions of ≤ 120 min or > 120 min per week, (6) training designs with a high-intensity aerobic session part of 15 min or less and separately those with greater than 15 min duration.

## Results

### Study selection

**Figure 1:**
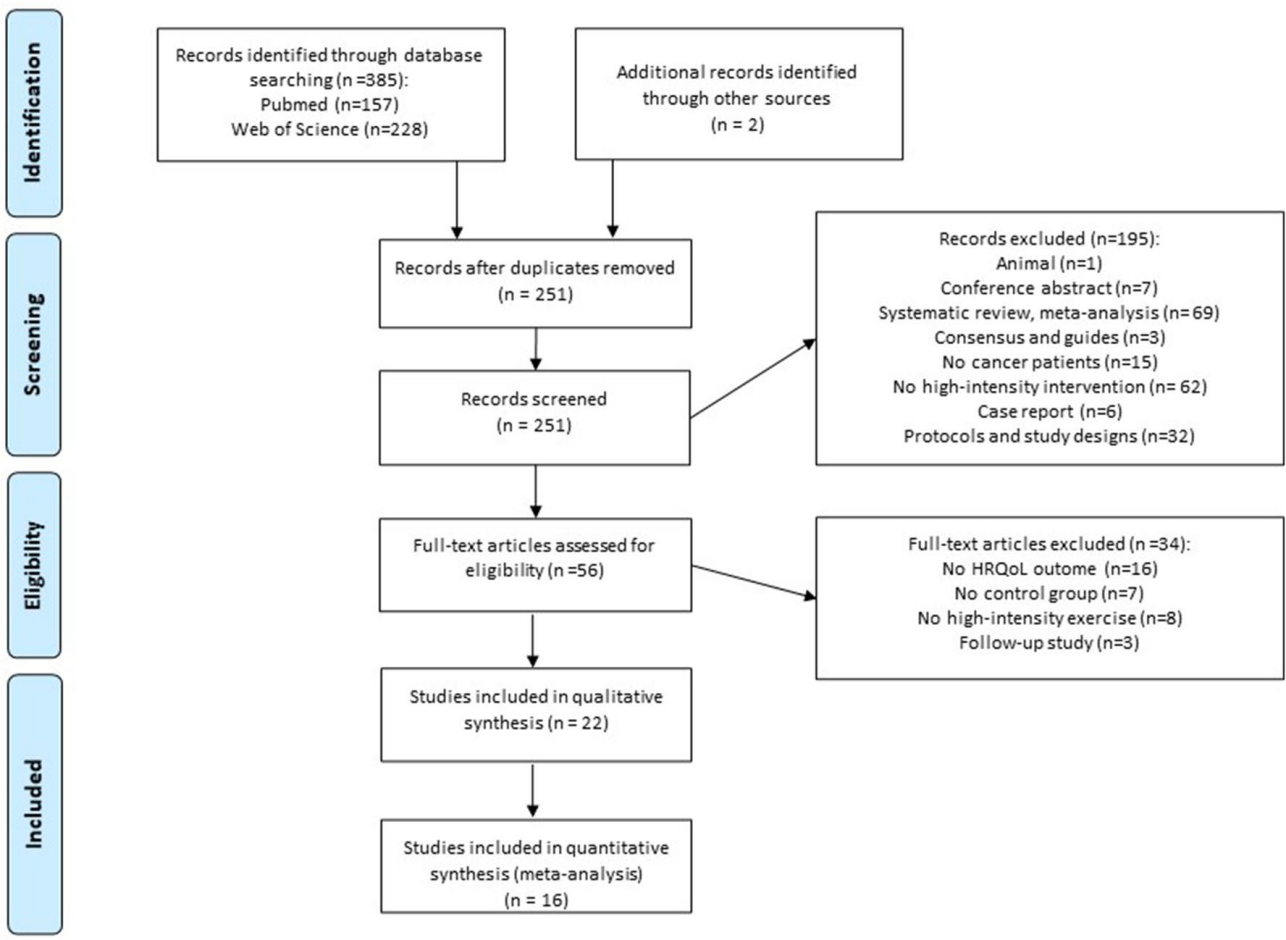
Study flow diagram.

Figure 1 sets out the data from the study selection process. The search obtained 385 articles, 157 in the PubMed database and 228 in Web of Science. Two more papers were identified in the references of articles and were therefore included^50,51^. One hundred thirty-five of the found studies were duplicated, so 251 were screened by examining the title and abstract. Following the exclusion criteria, one animal intervention, seven conference abstracts, 69 reviews, three consensus or guideline writings, 15 studies not focused on cancers, 62 not involving a high-intensity intervention, six case reports, and 32 study designs were removed. Fifty-six articles were full-text analyzed. From those, 16 were excluded because they did not have HRQoL as a variable, seven did not include a CG, eight did not carry out a HIT programme (most of them were respiratory exercises), and three were follow-up studies. In total, 22 studies were eligible for the systematic review, and from those sixteen had data to be included in the meta-analysis process.

### Risk of bias

The risk of bias was evaluated using the PEDro scale and ranged from 3 to 8, see Table 1 (being 10 the best score of the scale). The mean of the scores was 6.3. All the articles fulfilled Items 1 (“the election criteria were specified”) and 10 (“the results of between-group statistical comparisons are reported for at least one key outcome”). Item 5 “there was blinding of all subjects” and 6 “there was blinding of all therapists who administered the therapy” were only reaches by two of the includes studies^52^.

**Table 1:** Risk of bias using PEDro scale.

Validity	External item	Internal items								Statistic items		Total score
*Study*	*1*	*2*	*3*	*4*	*5*	*6*	*7*	*8*	*9*	*10*	*11*	
Pereira et al. (2020)	Y	Y	Y	Y	N	N	N	Y	N	Y	Y	6
Mijwel et al. (2018)	Y	Y	Y	Y	N	N	N	Y	Y	Y	Y	7
Christensen et al. (2018)	Y	Y	N	Y	N	N	N	Y	Y	Y	N	5
Adams et al. (2018)	Y	Y	Y	Y	N	N	Y	Y	Y	Y	Y	8
Persoon et al. (2017)	Y	Y	Y	Y	N	N	Y	Y	Y	Y	Y	8
Brunet et al. (2017)	Y	N	N	N	N	N	N	Y	N	Y	Y	3
Van Waart et al. (2017)	Y	Y	Y	Y	N	N	N	Y	N	Y	Y	6
Dunne et al. (2016)	Y	Y	Y	Y	N	Y	Y	Y	N	Y	Y	8
Toohey et al. (2016)	Y	Y	N	Y	N	N	N	Y	N	Y	Y	5
Waked,et al. (2016)	Y	Y	Y	Y	N	N	N	Y	N	Y	Y	6
Schmitt et al. (2016)	Y	Y	Y	Y	N	N	N	Y	N	Y	Y	6
Edvardsen et al. (2015)	Y	Y	Y	Y	N	N	N	Y	Y	Y	Y	7
Martin et al. (2015)	Y	Y	Y	Y	Y	N	N	Y	N	Y	Y	7
Moller et al. (2015)	Y	Y	Y	Y	N	N	Y	N	N	Y	Y	6
Kampshoff et al. (2015)	Y	Y	Y	Y	N	N	Y	N	Y	Y	Y	7
Van Wart et al. (2015)	Y	Y	N	Y	N	N	N	Y	Y	Y	Y	6
Midtgaard et al. (2013)	Y	Y	Y	Y	N	N	N	N	Y	Y	Y	6
Andersen et al. (2013)	Y	Y	Y	Y	N	N	N	N	Y	Y	Y	6
Cormie et al. (2013)	Y	Y	Y	Y	N	N	N	Y	Y	Y	Y	7
Hwang et al. (2012)	Y	Y	Y	Y	N	N	Y	N	N	Y	Y	6
Adamsen et al. (2009)	Y	Y	Y	Y	N	N	N	Y	Y	Y	Y	7

Y: Yes, the item was satisfied in the experimental protocol; N: No, the item was not satisfied in the experimental protocol Items: (1) Eligibility criteria; (2) Random allocation; (3) Concealed allocation; (4) Similarity of the groups at baseline; (4, 7–11) Key outcomes; (5–7) Blinding process; (8) Final measure with 85% of the initial sample size; (9) intent-to-treat analysis; (10) Between-group comparisons report; (11) Point and variability measures.

### Characteristics of the participants

Table 2 shows the meta-analysis participants’ baseline characteristics. The information from the articles included in the systematic review but not in the meta-analysis are presented in Supplementary Table S1 and Table S2. The global sample size of the systematic review was 2457, composed of 1080 participants of HIEG, 385 which participated in LMEG, and 992 from inactive CG and. Participants aged ranged from 27.8 to 72 with a mean of 51.56 in HIEG, 53.35 years in LMEG, and 51.6 in CG Patients' mean BMI ranged from 22.6 to 31 kg/m^2^ and often were not regularly physically active.

**Table 2:** Baseline characteristics of the participants included in the meta-analysis.

Study	Design	Group	Sample size (% of females)	Age (SD)	Cancer type (%)	Treatment	Timing	BMI
Egegaard et al. (2019)	Feasibility Study	CG	n = 7 (71.4%)	65 (4.7)	Non-smallcell lung Cancer (NSCLC)	Chemoradiotherapy	During	24.2 (1.9)
		HIEG	n = 8 (62.5%)	64 (5.8)			During	24.1 (4.4)
Mijwel et al. (2018)	Randomized Clinical Trial	CG	n = 60 (100%)	52.6 (10.2)	Breast cancer	Chemotherapy	During	NR
		HIEG-R	n = 74 (100%)	52.7 (10.3)				NR
		HIEG-A	n = 72 (100%)	54.4 (10.3)				NR
Adams et al. (2018)	Phase 2 Randomized Controlled Trial	CG	n = 28 (0%)	43.3 (9.9)	Testicular cancer	Surgery (96.4%) Radiotherapy (17.9%) Chemotherapy (28.6%)	After	27.9 (4.2)
		HIEG	n = 35 (0%)	44.0 (11.6)		Surgery (88.6%) Radiotherapy (17.1%) Chemotherapy (42.9%)	After	27.2 (5.0)
Van Waart et al. (2017)	Pilot trial	CG	n = 8 (10.6%)	56.7 (10.6)	Colon cancer	Surgery (25%) Radiotherapy (13%) Prescribed chemotherapy (100%)	During	23.5 (3.1)
		HIEG	n = 7 (71%)	57.7(13.2)		Surgery (57%) Prescribed chemotherapy (100%)	During	25.1(4.2)
		LIEG-H	n = 8 (38%)	60.1 (7.3)		Surgery (6%) Prescribed chemotherapy (100%)	During	23.6 (2.1)
Persoon et al. (2017)	Randomized controlled trial	CG	n = 55 (33%)	56	Multiple myeloma (53%) (Non-)Hodgkin lymphoma (47%)	NR	After transplanta-tion	NR
		HIEG	n = 54 (46%)	53.5	Multiple myeloma (54%) (Non-)Hodgkin lymphoma (46%)	NR	After Transplanta-tion	NR
Toohey et al. (2016)	Pilot study	HIEG	n = 8 (100%)	47.25(13.49)	Colon (6.25%) Cervical (6.25%) Melanoma (6.25%) Ovarian (12.5%) Breast (56.25%) Breast and uterine (6.25%) Breast and liver (6.25%)	Surgery (18.75%) Surgery + chemotherapy (12.5%) Surgery + radiation (6.25%) Surgery + chemotherapy + endocrine (12.5%) Surgery + chemotherapy + radiation + endocrine (50%)	After	NR
		MIEG	n = 8 (100%)	55.88 (11.81)			After	NR
Schmitt et al. (2016)	Single arm, non- randomized	HIEG	n = 13 (100%)	53 (8)	Breast (85%) Ovarian (8%) Non-invasive urotelial (8%) Metastases (15%)	Surgery (100%) Chemotherapy (54%) Radiation (69%) Antihormonal (69%)	After	27.0 (5.3)
		LIEG	n = 13 (100%)	54 (9)	Breast (77%) colon (8%) vaginal (8%) Non-Hodgkin0s lymphoma (8%) Metastases (8%)	Surgery (100%) Chemotherapy (69%) Radiation (69%) Antihormonal (54%)	After	26.2 (4.3)
Dunne et al. (2016)	Randomized clinical trial	CG	n = 17 (23.5%)	62	Colorectal liver metastasis	Chemotherapy (60%)	Before	29.7 (4.2)
		HIEG	n = 20 (35%)	61		Chemotherapy (58.82%)	Before	29.7 (4.2)
Kampshoff et al. (2015)	Randomized controlled trial	CG	n = 92 (78%)	54 (10.9)	Breast (63%) Colon (17%) Ovarian (6%) Lymphoma (9%) Cervix (2%) Testicles (4%)	Surgery (88%) Radiation (53%) Surgery + radiation (51%) Immunotherapy (20%) Homonal therapy (47%)	After	NR
		HIEG	n = 91 (80%)	54 (11.0)	Breast (68%) Colon (17%) Ovarian (4%) Lymphoma (10%) Testicles (1%)	Surgery (91%) Radiation (51%) Surgery + radiation (45%) Immunotherapy (18%) Homonal therapy (50%)	After	NR
		LMIEG	n = 95 (82%)	53 (11.3)	Breast (65%) Colon (20%) Ovarian (3%) Lymphoma (9%) Cervix (2%)	Surgery (92%) Radiation (43%) Surgery + radiation (41%) Immunotherapy (26%) Homonal therapy (42%)	After	NR
Martin et al. (2015) c)	Randomised controlled trial	CG	n = 35 (0%)	66.9 (6.6)	Prostate cancer	Surgery (77.14%) Radiation (28.57%) Brachytherapy (11.43%) ADT (20%)	After	28 (3.7)
		HIEG	n = 27 (0%)	65.3 (7)		Surgery (81.48%) Radiation (18.52%) Brachytherapy (11.11%) ADT (11.11%)	After	27.6 (4.1)
		LIEG	n = 25 (0%)	65 (6.3)		Surgery (92%) Radiation (8%) ADT (12%)	After	26.4 (2.8)
Martin et al. (2015) a)	Randomised controlled trial	CG	n = 40(100%)	57.2 (9.8)	Breast cancer	Surgery (100%) Chemotherapy (67%) Radiation (71%) Hormone (98%)	After	26.3 (5.2)
		HIEG	n = 13 (100%)	53.5 (9)		Surgery (100%) Chemotherapy (77%) Radiation (54%) Hormone (85%)	After	27.9 (5.3)
		LIEG	n = 19(100%)	58.2 (9.6)		Surgery (100%) Chemotherapy (63%) Radiation (90%) Hormone (82%)	After	26.6 (4.8)
Van Waart et al. (2015)	Randomized Clinical Trial	CG	n = 77 (100%)	51.6 (8.8)	Breast cancer	Surgery (78%) Radiation (78%)	During	NR
		HIEG	n = 76 (97%)	49.9 (8.4)		Surgery (74%) Radiation (79%)	During	NR
		LIEG-H	n = 77(100%)	50.5 (10.1)		Surgery (81%) Radiation (78%)	During	NR
Møller et al. (2015)	Randomised feasibility study	CG	n = 16 (12.5%)	46.95 (9.19)	Colon and breast cancer	Chemotherapy	During	25.54 (4.9)
		HIEG	n = 15 (7.14%)	57.17 (10.51)	Colon and breast cancer	Chemotherapy	During	24.39 (5.27)
		LIEG	n = 77(100%)	48.49 (8.41)	Colon and breast cancer	Chemotherapy	During	23.8 (2.59)
Edvardsen et al. (2015)	Randomised controlled trial	CG	n = 31 (52%)	65.9 (8.5)	Lung cancer	Surgery (100%) Chemotherapy (29%) Radiation (13%)	After surgey	25.1 (5.2)
		HIEG	n = 30 (57%)	64.4 (9.3)		Surgery (100%) Chemotherpy (30%) Radiation (10%)	After surgey	25.4 (5.1)
Cormie et al. (2013)	Randomised controlled trial	CG	n = 19 (100%)	58.6 (6.7)	Breast cancer	Surgery (89.5%) Chemotherapy (63.2%) Radiotherapy (89.5%) Hormonotherapy (57.9%)	After	28.2(6.0)
		HIEG	n = 22 (100%)	56.1 (8.1)		Surgery (90.9%) Chemotherapy (90.9%) Radiotherapy (77.3%) Hormonotherapy (63.6%)	After	30.8 (6.5)
		LIEG	n = 21 (100%)	57.0 (10.0)		Surgery (100%) Chemotherapy (90.5%) Radiotherapy (81.0%) Hormonotherapy (66.7%)	After	30.4(5.7)
Andersen et al. (2013)	Randomised controlled trial	CG	n = 107 (72%)	47.8 (10.4)	Breast (47.66%) Bowel (14.02%) Ovaries (8.41%) Testicles (6.54%) Oesophagus (0.93%) Brain (1.87%) Cervix (1.87%) Pharynx (0.93%) Pancreas (1.87%) Stomach (0.93%) Hematological (9.43%)	NR	During	NR
		HIEG	n = 106 (79.2%)	47.1 (10.8)	Breast (49.05%) Bowel (13.21%) Ovaries (10.38%) Testicles (6.6%) Oesopagus (0.94%) Brain (0.94%) Cervix (1.88%) Pharynx (1.88%) Pancreas (0.94%) Stomach (0.94%) Hematological (10.38%)	NR	During	NR
Hwang et al. (2012)	Randomised controlled trial	CG	n = 11 (36.4%)	58.5 (8.2)	Lung cancer	Surgery (36.4%) Chemotherapy (45.6%) Radiotherapy (45.5%)	During	23.1 (2.6)
		HIEG	n = 13 (61.5%)	61.0 (6.3)		Surgery (69.3%) Chemotherapy (76.9%) Radiotherapy (61.5%)	During	22.6 (2.4)
Adamsen et al. (2009)	Randomized control trial	CG	n = 134 (70.9%)	47.2 (10.6)	Breast (44.03%) Bowel (12.68%) Ovaries (8.2%) Testicular (6.7%) Oesophagus (2.23%) Brain (2.98%) Cervix (1.5%) Pharynx (0.74%) Pancreas (1.5%) Stomach (1.5%) Hematological malignancies (11.2%)	Chemotherapy	During	NR
		HIEG	n = 135 (74.8%)	47.2 (10.7)	Breast (44.44%) Bowel (13.33%) Ovaries (11.85%) Testicular (5.18%) Oesophagus (1.48%) Brain (0.74%) Cervix (2.96%) Pharynx (1.48%) Pancreas (0.74%) Stomach (0.74%) Hematological malignancies (9.63%)	Chemotherapy	During	NR

CG: control group, HIEG: high-intensity exercise group, HIEG-R: high-intensity resistance exercise group, HIEG-E: high-intensity endurance exercise group, LIEG: low-intensity exercise group, LMIEG: low to moderate exercise group, NR: not reported.

The selected articles involved different types of cancer. Some papers specified the intervention in one type of cancer, such as breast cancer^39,51,53–57^ (being the most common in the studies included), colon cancer^51,58,59^, lung cancer^60–62^ prostate cancer^54^, testicles cancer^63^ or rectal cancer^50^. Other authors designed programmes mixing participants with different kinds of cancer^37,57,64–69^. Moreover, the exercise interventions found could be distinguished by the timing within the cancer pathway: before^59^, during^50,51,53,56–58,60,61,68–71^ or after treatment^54,62,65,66,72,73^.

### Characteristics of the exercise programs

The intervention descriptions are reported in Table 3 (meta-analysis articles) and in Supplementary Table S3. The mean duration of interventions was 12 weeks and the median was 10 weeks. Interventions were three weeks^66^, six weeks^50,51,68,69^, seven weeks^71^, eight weeks^54,56,61^ twelve weeks^39,72–75^, sixteen weeks^53^, eighteen weeks^65^, 36 weeks^55^ and 12 months^62^. HIEG participants trained with a mean frequency of 2.8 times/week, so most of the interventions programs were delivered 3 times/week^37,50,51,54,55,60–63,68,69,75,76^, although in some were 2 days/week^39,53,57,58,65,67,70^ and 5 days/week^71^. All high-intensity interventions were supervised and conducted indoors, except Schmitt et al. (2016), who evaluated the effects of a program performed outside on a paved uphill road^66^. Mean duration was 62.5 min but included sessions of 20 min^71,75^, 20 to 30 min^74^, 30 to 40 min^61,72^, 40 min^50^, 50 min^57,58^, 60 min^39,53,54,60,65^, 70 min^55^, 75 min^66,70^ and 90 min^51,56,68,69^.

**Table 3:** Description of the high-intensity exercise interventions included in the meta-analysis.

Study	Group	Duration	Sessions duration	Weekly frequency	Setting	Exercise description	Intensity progression and control	Attendance
Egegaard et al. (2019)	CG	7 weeks		Daily life		Activity tracker (Garmin®vívosmart®)		
	HIEG	7 weeks	20 min	5 times per week		5 min warm-up HIIT: 1st and 3rd cycle ergometer intervals: 5 × 30 s with 30 s rest2nd cycle ergometer interval: continuous cycling	Moderate-to-high intensity Warm-up: 50–60% (W Peak Power) 1st, 3^rd^ interval 80–95% (W Peak Power) 2^nd^ interval: 80% (W Power Peak) Additional control: HR	Sessions: 90.0% and adherence Simple size: 100%
Mijwel et al. (2018)	CG					Written American College of Sports Medicine exercise recommendations		
	HIEG-R	16 weeks	60 min	2 times/week	Exercise clinic	5 min aerobic warm-up HIIT cycle exercise: 3 × 3 min intervals with 1 min recovery Resistance: 8–12 high-load repetitions of the major muscle groups	Warm-up: 10–12 RPE Resistance: 70%-80% (RM) Aerobic: moderate 13–15 RPE HIIT: intervals at 16–18 RPE	Sessions: 68% Simple size: 88%
	HIEG-A	16 weeks	60 min	2 times/week	Exercise clinic	5 min aerobic warm-up HIIT cycle exercise: 3 × 3 min intervals with 1 min recovery Aerobic: 20 min of cycle ergometer, elliptical ergometer, or treadmill moderate continuous exercise	Warm-up: 10–12 RPE HIIT: intervals at 16–18 RPE From 70% RM to 80% RM	Sessions: 63%
Adams et al. (2018)	CG	12 weeks						
	HIEG	12 weeks	35 min	3 times/week	Supervised	5 min warm-up and cool-down HIIT:4 × 4 min intervals with 3 min active recovery	Warm-up: at ± 5% of the ventilatory threshold Intervals: from 75 to 95% VO2máx Recovery: 5%-10% of the ventilatory threshold	Sessions: 99% Simple size: 100%
Van Waart et al. (2017)	CG					Moderate intensity leisure-time sports		
	HIEG	From the first cycle of chemotherapy to 3 weeks after the last cycle	50 min	2 times/week	Supervised	Resistance: 20 min 6 large muscle groups 2 series of 8 repetitions Cardiovascular MHIT: 30 min + 30 min physically active 5 days/Week	Moderate to high Resistance: 80%RM Aerobic: 80% (predicted maximal workload) Adjustment: 1RM testing was repeated every 3 weeks Additional control: RPE	Sessions: 61%
	LIEG-H		30 min	5 times/week	Home-based	Written individual information	Low intensity: 12–14 RPE Additional control:Activity Diary	NR
Persoon et al. (2017)	CG	18 weeks						
	HIEG	18 weeks	60 min	1st–12th week 2 times/week 13th week 1 time/week	Physiotherapy center	High-intensity resistance: 6 standardized exercise muscles. Week 1–12: 2 series of 10 repetitions HIIT: 2 series of8 min cycling Week 1–8 30 s blocks with 60 s blocks Week 9–12 30 s blocks	Resistance: 65–80% RM Aerobic: 30 s blocks at 65% (maximal short exercise capacity) 60 s blocks at 30% (maximal short exercise capacity) Load adjustment every 4 weeks by a performing of the indirect 1-RM measurements and the steep ramp test	Sessions: 86% Simple size: 92.6%
Toohey et al. (2016)	HIEG	12 weeks	20 min	3 times/week	Supervised	5 min warm-up HIIT: 7 × 30 s intervals with 1 min rest 5 min cool-down	Intervals ≥ 85% (HRmax) From 3 intervals in the first session to 7 intervals in week 5 Additional control: RPE and blood pressure	Sessions: 93.75% Simple size: 100%
	MIEG	12 weeks	30 min	3 times/week	Supervised	5 min warm-up 20 min cycle continuos Aerobic 5 min cool-down	≤ 55% predicted maximal heart rate	NR
Schmitt et al. (2016)	HIEG	3 weeks 8 sessions		3 times/week	Outside (paved up-hill road)	5 min warm-up HIIT: 8 × 1 min intervals walking 2 min active recovery	Warm-up: 70% (HRpeak) Intervals: > 95% (HRpeak)	93% partipants all sessions
	LMIEG	3 weeks	75 min	6 sessions	Outside (paved up-hill road) and Inside	60 min walking 15 min indoor cycling	Cycling: 60% (HRpeak)	
Dunne et al. (2016)	CG	4 weeks						
	HIEG	4 weeks	warm-up + 30 min + cool-down	12 sessions	Clinic	Cycle ergometer exercise Warm-up HIIT: Intervals of high and moderate intensity	High intensity > 90% (VO2 peak) Moderate intensity > 60% (VO2peak)	Sessions: 99% Simple size: 95%
Kampshoff et al. (2015)	CG	12 weeks						
	HIEG	12 weeks	Depending on de week	2 times/week	Supervised	Resistance: six exercise large groups 2 series of 10 repetitions HIIT: 1st–4th week: 2 × 8 min cycling intervals 30 s + 60 s blocks 4th–end:2 × 8 min cycling intervals 30 s + 30 s blocks 5th week-end additional HIIT session: 8 min of cycling intervals 30 s + 30 s blocks and 8 min 3 × 5 min continuous ergometer with 1 min rest	Resistance: 70%-85% (RM) Aerobic: 30 s Interval 65% (MSEC) 60 s Interval:30% (MSEC) Continuous ergometer: 80% (HRR) Every four weeks, the training progress was evaluated utilizing the steep ramp test and RM test and the workload is adjusted accordingly	Sessions: 74% and more than 80% of the sessions Simple size: 92%
	LMIEG	12 weeks		2 times/week	Supervised	Resistance: six exercise large groups 2 series of 10 repetitions Interval aerobic: 1st–4th week: 2 × 8 min cycling intervals 30 s + 60 s blocks 4th–end: 2 × 8 min cycling intervals 30 s + 30 s blocks 5th week-end additional Aerobic session: 8 min of cycling intervals 30 s + 30 s blocks and 8 min 3 × 5 min continuos ergometer with 1 min rest	Resistance: 70%-85% (RM) Aerobic: 30 s Interval 45% maximum short exercise capacity (MSEC) 60 s Interval:30% (MSEC) Continuos ergometer: 40%-50% (HRR)	Sessions: 70%
Martin et al. (2015) c)	CG	8 weeks						
	HIEG	8 weeks	60 min	3 times/week	University clinic	25 min HIT 25 min resistance 10 min static stretching	Aerobic: 75%-80% (VO2 max) Resistance: 65–80% RM Increase 5% VO2 middle of the programme Additional control: HR	Sessions: 90% Simple size: 96%
	LIEG	8 weeks	60 min	3 times/week	University clinic	25 min Aerobic 25 min resistance 10 min static stretching	Aerobic: 60%-65% (VO2 max) Resistance: 50–65% RM Increase 5% VO2 middle of the programme	
Martin et al. (2015) a)	CG	8 weeks						
	HIEG	8 weeks	60 min	3 times/week	University clinic	25 min HIT 25 min resistance 10 min static stretching	Aerobic: Week 1- 4 75% (VO2 max) Week 5–8 80% (VO2max) Resistance: 65–80% RM Increase 5% VO2 middle of the programme Additional control: HR	Sessions: 90% Simple size: 96%
	LIEG	8 weeks	60 min	3 times/week	University clinic	25 min Aerobic 25 min resistance 10 min static stretching	Aerobic: Week 1- 4 60% (VO2 max) Week 5–8 65% (VO2max) Resistance:50–65% RM Increase 5% VO2 middle of the programme	
Van Waart et al. (2015)	CG					Moderate intensity leisure-time sports		
	HIEG	From the first cycle of chemotherapy to 3 weeks after the last cycle	50 min	2 times/week	Supervised	Resistance: 20 min 6 large muscle groups 2 series of 8 repetitions Cardiovascular MHIT: 30 min + 30 min physically active 5 days/Week	Moderate to high Resistance: 80%RM Aerobic: 50%-80% (predicted maximal workload) Adjustment: Resistance: 1 RM testing every 3 weeks Aerobic: Borg Scale, with a threshold of less than 12 for the increase and more than 16 for decrease of intensity Additional control: RPE	NR
	LIEG-H		30 min	5 times/week	Home-based	Written individual information	Low intensity: 12–14 RPE Additional control: Activity Diary	NR
Møller et al. (2015)	CG	12 weeks						
	HIEG	12 weeks	90 min (hiit sessions)	9 h/ week (HIIT and Low-intensity sessions)	Copenhagen University Hospital	High-intensity sessions: 30 min warm-up HIT resistance: 45 min, 3series of 5–8 repetitions HIIT:15 min cardiovascular cool-down (stretching and coordination training) Low- intensity sessions: 30–90 min of body awareness, relaxation or massage	Resistance:70–100% RM- 5.5 METSs Aerobic: 70–250 W, 85–95% (HRmax) 15 METs	Sessions: 74% Simple size: 82%
	LIEG-H	12 weeks			At home	Low/moderate recreational physical activity level of 30 min/day and 10 000 steps/day, five times/week	Podometer data	NR
Edvardsen et al. (2015)	CG	20 weeks						
	HIEG	20 weeks	60 min	3 times/week	Fitness centers	Warm-up HIIT: Interval uphill treadmill walking Resistance 3 series of leg press, leg extension, back extension, seat row, bicep curls, and chest-and-shoulder press	Intervals 80–95% (HRpeak) Resistance: 6–12 RM Increase of Interval intensity and duration based on the patient’s improvement, ability to cope with dyspnoea and feelings of well-being or fatigue on each exercise day Additional control: RPE	Sessions: 88 ± 29% Simple size: 83%
Cormie et al. (2013)	CG	3 months						
	HIEG	3 months	60 min	2 times/week	Supervised	10 min warm-up HIT resistance: 1–4 sets of 6 exercise upper body and 2 lower body 5 min cool-down	Resistance: 75%-85% RM using 10–6 RM Resistance increased 5–10% for the next set and/or training session if participants were able to perform more repetitions than the RM’s Additional control: RPE	NR
	LIEG	3 months	60 min	2 times /week	Supervised	10 min warm-up Resistance: 1–4 sets of 6 exercise upper body and 2 lower body 5 min cool-down	Resistance: 55%-65% RM using 20–15 RM Additional control: RPE	NR
Andersen et al. (2013)	CG							
	HIEG	6 weeks	90 min (hiit sessions)	9 h/ week (HIIT and Low-intensity sessions)	Copenhagen University Hospital	High-intensity sessions: 30 min warm-up HIIT:10 min cycling interval Cool-down (stretching and coordination training) Resistance Low- intensity sessions: 30–90 min of body awareness, relaxation or massage	Intervals: 85–95% (HRpeak)	NR
Hwang et al. (2012)	CG					General exercise instructions and Theraband Elastic Band		
	HIEG	8 weeks	30–40 min	3 times/week	Clinic	Treadmill o cycling ergometer sessions 10 min warm-up HIIT:2–5 min intervals with an active recovery 5 min cool-down	Intervals: 80% (VO2peak) 15–17 RPE Recovery: 60% (V02peak) 11–13 RPE Intensity and duration were adjusted every 1–2 weeks based on the individual’s exercise response Additional control: HR, blood pressure and oxygen saturation	Sessions: 71.2% Simple size: 85%
Adamsen et al. (2009)	CG							
	HIEG	6 weeks	90 min (HIT sessions)	9 h/ week (HIT and Low intensity sessions)	Copenhagen University Hospital	High-intensity sessions: 30 min warm-up HIT resistance 45 min: 3 series of 5–8 repetitions HIIT: 15 min cardiovascular interval training: cool-down (stretching and coordination training) Low- intensity sessions: 30–90 min of body awareness, relaxation or massage	Resistance:70–100% RM- 5.5 METSs Aerobic: 70–250 W, 85–95% (HRmax) 15 METs	Sessions: 70.8% Simple size: 87,4%

CG: control group, HIEG: high-intensity exercise group, LIEG: low-intensity exercise group, LMIEG: low to moderate exercise group, HIT: high-intensity training, HIIT: high-intensity interval training, MHIT: moderate to high intensity training, METs: Metabolic equivalent of task, RM: maximum repetition, HR: heart rate, RPE: the rating of perceived exertion, MSEC: maximum short exercise capacity, VO_2_: oxygen consumption, NR: not reported.

All HIT components included a cardiovascular exercise component, except Cormie et al. (2013), which included resistance training only^39^. In some interventions, HIT was conducted in interval bouts of 30 s^65,71,73^, 1 min^66^ 3 min^53^, 4 min^63^, 5 to 8 min^61^. Others incorporated HIT utilizing continuous aerobic training protocols^51,54,57,68,69^. Across the studies, there were a variety of methodologies used to set high intensity depending on VO_2_: 95% VO_2_ max.^63^, > 90% VO_2_ peak, 80% VO_2_peak^61^, 80% VO_2_max^54^; based on heart rate: > 85% HRmax^75^, 95% HRpeak^51,60,66,68,69^; based on power: 95% Wpeak power^71^; based on Borg’s Rating of perceived exertion scale: 18 of the Borg’s Rating of perceived exertion scale^53^; and based on the maximum short exercise capacity (MSEC)^65,67^, 80% of predicted maximal workload^57,58^. Of equal importance, the prescribed rest during HIT varied from 30 s^71^ to 1 min^53,73,75^, 2 min^66^, or 3 min of active recovery^63^. Most of the interventions supplemented the HIT component either with resistance training^51,53,54,57,58,60,62,65,68–70,73^ Other studies complemented HIT with low-intensity sessions like body awareness, relaxation, or massage^51,68,69^. Data regarding participants' adherence are presented in Table 3 and Supplementary data (Table S3). The mean percentage rate of sessions completed for participants in each group was HIEG 76.7%; LMEG 72.9%; aerobic exercise 82.3%; and resistance exercise 74.0%.

### Health-related quality of life outcomes

According to the EORTC QLQ-C30 scoring manual, the results, including SF-36 and FACT-G questionnaires, were divided into three categories shown in Table 4: Global health status, Functional scales, and Symptom scales^77^. Below are the results of the exercise programmes characteristics showing the interventions needed to achieve higher HRQoL benefits. Tthe supplementary data included explain the description of each HRQoL dimension results with their corresponding figure resume (from Figure S1 to Figure S10), and all the meta-analyses performed are reported in the supplementary data (from Supplementary Figure S11 to Supplementary Figure S61).

**Table 4:**
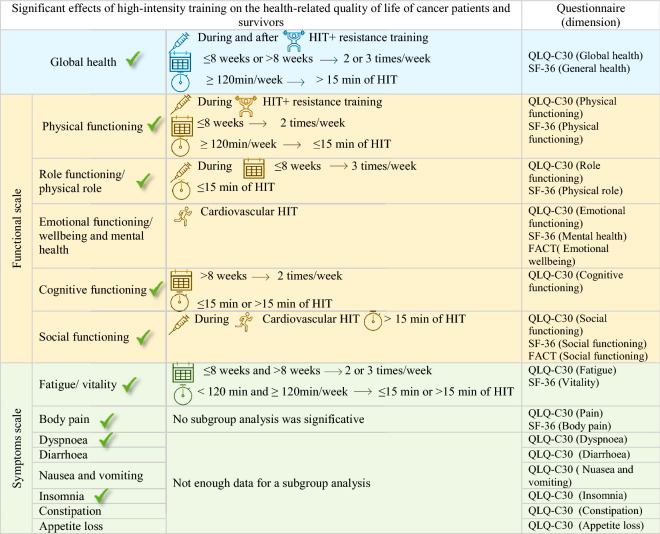
Sum of HRQoL results obtained and the corresponding questionnaires used.

: Significant differences between the control group and the high-intensity exercise group.

No comparison between the high-intensity exercise group and the low-moderate group was significant.

QLQ-C 30: European Organisation for Research and Treatment of Cancer quality of life Questionnaire, SF-36: Short-form 36, FACT-G: Functional Assessment of Cancer Therapy (General).

#### Physical exercise and treatment timing

When the HIEG exercise programs were implemented during cancer treatments, physical functioning (p = 0.0005, with SMD of 0.42 and a 95% CI from 0.18 to 0.66) role functioning (p = 0.0003, with SMD of 0.35 and a 95% CI from 0.16 to 0.54) and social functioning (p = 0.03, with SMD of 0.12 and a 95% CI from 0.01 to 0.23) seemed to improve more than the CG. In contrast, those variables were not significantly improved from after-treatment interventions. Moreover, outcomes of global health dimensions showed similar between-group differences in exercise programs conducted during (p = 0.02, with SMD of 0.22 and a 95% CI from 0.03 to 0.40) and after (p = 0.003, with SMD of 0.30 and a 95% CI from 0.10 to 0.50) cancer treatments.

#### Intervention length

Results showed higher between-group differences (HIEG vs CG) when performing HIT in exercise programs of ≤ 8 weeks duration, including physical function (p = 0.04, with SMD of 0.04 and a 95% CI from 0.01 to 0.45) and role functioning (p = 0.02, with SMD of 0.26 and a 95% CI from 0.04 to 0.49). For HIT programs lasting more than 8 weeks there was no significant between-group differences (physical function p = 0.05, role functioning p = 0.07). However, cognitive functioning reached higher significant differences between the CG and the EG in interventions longer than 8 weeks (p = 0.04, with SMD of 0.20 and a 95% CI from 0.01 to 0.40). All HIT durations showed significant differences between CG and HIEG in the global health dimensions (≤ 8 weeks: p = 0.04; > 8 weeks: p = 0.002) and fatigue (≤ 8 weeks: p = 0.008; > 8 weeks: p = 0.001).

#### Exercising frequency

The physical (p = 0.005, with SMD of 0.37 and a 95%CI from 0.11 to 0.62) and cognitive functioning (p = 0.003, with SMD of 0.25 and a 95% CI from 0.08 to 0.42) dimensions showed significant between-group differences with higher improvements in HIEG *vs* CG in interventions conducted 2 times/week. In comparison, 3 times/week programs did not show significant between-group differences (physical p = 0.09; cognitive p = 0.18). However, patients in the HIEG scored higher than the CG in role functioning with a frequency of 3 times/week (p = 0.04, with SMD of 0.21 and a 95% CI from 0.01 to 0.42), while no significant results were observed in interventions with lower frequency (p = 0.05). All of the reported exercise frequencies showed significant differences between CG and HIEG in global health (2 times/week: p = 0.002; 3 times/week: p = 0.03) and fatigue (2 times/week: p = 0.005; 3 times/week: p = 0.001) dimensions.

#### Minutes of exercise per week

Results showed significant improvements in HIEG compared to CG in global health (p = 0.03, with SMD of 0.18 and a 95% CI from 0.02, to 0.42) and physical functioning (p = 0.006, with SMD of 0.24 and a 95% CI from 0.07 to 0.40) only when patients exercised at least 120 min/week. The fatigue dimension was significantly improved in both shorter (< 120 weekly minutes: [p = 0.01]) and longer bouts per week (≥ 120 weekly minutes [p = 0.0005]).

#### Type of exercise programme

Interventions that combined resistance training and HIT showed better improvements compared to the CG in global health (p = 0.0008, with an SMD of 0.25 and a 95% CI from 0.10 to 0.39) and physical functioning (p = 0.0006, with an SMD of 0.34 and a 95% CI from 0. 15 to 0.53). Whereas programs involving only cardiovascular/aerobic HIT achieved significant between-group differences in emotional (p = 0.007, with SMD of 0.36 and a 95% CI from 0.10 to 0.63) and social functioning (p = 0.03, with SMD of 0.29 and a 95% CI from 0.03 to 0.55).

#### High-intensity training part duration

Patients who participated in programs with components of HIT totalling ≤ 15 min increased their physical (p = 0.003, with an SMD of 0.29 and a 95% CI from 0.10 to 0.48) and role function (p = 0.0004, with an SMD of 0.34 and a 95% CI from 0.15 to 0.54) in contrast to the CG. However, in HIT lasting longer than 15 min, no significant between-group differences were seen in those variables (p = 0.05 and p = 0.27, respectively). Moreover, when the HIT portion lasted more than 15 min, global health (p = 0.001, with an SMD of 0.32 and a 95% CI from 0.13 to 0.51) and social functioning (p = 0.03, with an SMD of 0.17 and a 95% CI from 0.01 to 0.33) seemed to improve more in the HIEG than in the CG. For cognitive (≤ 15 min: p = 0.04; > 15 min: p = 0.02) and fatigue (≤ 15 min: p = 0.0005; > 15 min: p = 0.01) improvements, both longer and shorter HIT durations showed significant between-group differences.

Furthermore, HIEG reported significant improvements compared to a CG in the overall comparison of bodily pain, dyspnea and insomnia (p = 0.02, with an SMD of − 0.18 and a 95% CI from − 0.21 to − 0.02 in pain analysis; p = 0.002 with an SMD of − 0.34 and a 95% CI from − 0.55 to − 0.13 in the dyspnoea results and p = 0.003, with an SMD of − 0.29 and a 95% CI from − 0.47 to − 0.10 in insomnia). There were no significant between-group differences in diarrhea, nausea, constipation, and appetite loss dimensions.

The meta-analysis did not include the global calculation of HRQoL because of the data heterogeneity from the different questionnaires’ measures, despite this there were significant improvements in most of the articles analyzed^54–56,59,62,69,74,75^ as the Supplementary Tables S4 and S5 report.

## Discussion

This study aimed to explore the effect HIT on HRQoL dimensions in cancer patients and survivors. We also aimed to evaluate the optimal characteristics of HIT for dimensions of HRQoL with respect to intervention timing and cancer treatment, mode of exercise, and exercise dose. We found that HIT improves global quality of life, physical functioning, role functioning, social functioning, cognitive functioning, fatigue, pain, dyspnoea, and insomnia, compared to an inactive control group. The inclusion of resistance training seemed critical to improvements in global health and physical functioning. No significant differences were found when the effects of HIT were compared to low to moderate-intensity exercise. Improvements in HRQoL were observed during cancer treatment when training occured for more than eight weeks, with a frequency of 2 days/week, and a volume of at least 120 min/week with the HIT component duration in each session of at least 15 min.

Global health and physical function were the most commonly reported variables studied in exercise and cancer reviews, and findings here suggest that HIT consistently shows improvements in these outcomes compared to an inactive control group^32,33^. Data support positive global health changes with intense exercise^37^, but are contrary to Mugele et al. (2019), who focused solely on High Intensity Interval Training (HIIT) in their systematic review^38^. The broader definition of HIT might explain the data we observed here, but it is clear further studies are required to understand the role of HIT, including HIIT, specifically on HRQoL outcomes in cancer.

The subgroup analysis made regarding an intervention’s timing showed statistically positive effects in the global health dimension, physical functioning, role functioning, and social functioning during cancer treatments. Only the global health dimension showed a positive increase in after-treatment HIT. In line with our findings, the functional variables of HRQoL decrease progressively across chemotherapy^78^. Our data suggest that this decline might be moderated with HIT, particularly regarding depression and anxiety, function^79^ and activities of daily living^80^. Most of the negative side effects of cancer and its treatments are related to reduced physical functioning, reduced mobility due to surgery or chemotherapy^81^, lymphedema^82^, negative body composition changes as sarcopenia^83^, or osteoporosis^84^. Providing opportunities to mitigate these deleterious effects through HIT is highly important since more than half of all cancer patients develop a mobility disability because of the disease and its treatments’ adverse side-effects^85^.

Exercise interventions can and should be an important therapeutic modality prior to the onset of medical treatment^86^. Exercise has been shown to increase baseline physical functioning, reduce treatment-related impediments^30^, and help a patient maintain overall strength during treatment^87^. Post-treatment exercise can help the patient return to baseline and reduce subsequent side-effects^88^. The meta-analysis underlined the important role of resistance training in improving global health and physical function. Incorporating strength training in HIT programs is likely to increase muscle function, reduce the risk of sarcopenia, and reduce the risk of mortality^89^ and treatment toxicity^90^. This has been shown independent of age, cancer stage, or BMI^91^, and is partly explained through an anti-inflammatory response^92^. Further, resistance training may regulate deficiencies in skeletal muscle and adipose tissue known as cachexia^93,94^. However, it should be noted that interventions which included resistance training had lower adherence rates compared to aerobic exercise, which has been reported in other chronic disease patients^95^. Poor adherence might also explain why social and emotional functioning only significantly increased in the aerobic component programs, not in the resistance exercise modalities. To improve adherence, researchers and exercise specialists might wish to adopt a co-production approach, seeking to co-create the specific training strategies with people who have a cancer diagnosis, taking into account what matters most to them^95^.

Cancer and its associated treatments can cause severe side-effects during drug therapy, with pain and fatigue the most common^96^. Fatigue-related to cancer is reported by 70% of cancer patients^97^. The complaint of cancer-related fatigue is associated with immune response dysregulation, inflammation, metabolic and mitochondrial function impairment, neuroendocrine function impairment, and genetic biomarkers^98^; however, with exercise, these parameters can be improved^99^. To decrease fatigue, HIT, as well as other exercise modes, seems to be effective^30^, possibly more so than pharmacological or psychological therapies^100^. Other symptoms like pain, insomnia, and dyspnea also appear to improve via exercise^35^and without aggravating cancer symptoms, although this requires further investigation^101^.

Interventions lasting more than 8 weeks reported greater increases in HRQoL compared to shorter duration programs, which is consistent with a previous review of HIT interventions^37^. Greater improvements across a range of cancer-related outcomes were observed with exercising 3 times/week compared to training 2 times/week, except for role functioning (3 times/week). The American College of Sports Medicine recommends exercising two to three times/week^101^, which agrees with the findings of our study and a previous meta-analysis^37^. Three exercise sessions per week will also make it easier for individual cancer patients to achieve 120 min of weekly exercise, which seems to be important for increasing HRQoL, particularly when each session includes at 15 min of HIT. Some programs have included family members with hospitalized patients^102^.

This article presents valuable information about the role of high-intensity exercise as part of treatment and recovery in cancer, specifically in terms of HRQoL. The data from the systematic review and meta-analysis should be viewed in the light of the following limitations. Only articles written in English or Spanish were included, so not all the available information was analyzed. The intervention description, as well as the subgroup meta-analysis, was undertaken with the published available details. Where a study had incomplete data (e.g. sessions’ duration, HIT minutes, after intervention mean and standard deviation, etc.), data were omitted to the corresponding subgroup calculation. For the meta-analysis procedure, data from at least three articles were needed to make a subgroup analysis. Thus, assessments concerning the cancer type and all the subgroups analysis considering each intervention characteristic were not possible. Therefore, more information could be added with further studies. It must be considered that three of the included articles combined HIT with body awareness, relaxation, or massage interventions, each of which could influence HRQoL.

## Conclusion

This is the first meta-analysis exploring the effects of HIT on the HRQoL of cancer patients and survivors. Data from this systematic review and meta-analysis suggests that HIT as part of exercise therapy for people with a cancer diagnosis can improve global health and provide physical, cognitive, and social functioning benefits compared to controls. In addition, fatigue, bodily pain, dyspnea, and insomnia decreases can be achieved with HIT, all with similar outcomes observed using low-moderate intensity exercise. Dimensions of HRQoL showed the largest positive effects when the programs were delivered as part of cancer treatment and included resistance training. Ultimately, exercise programs may need to be longer than 8 weeks, with a HIT frequency of 2 times/week, and a total duration of at least 120 min/week, including a HIT component of more at least 15 min, to achieve the highest return in HRQoL. However, as it is the first meta-analysis about the effects of HIT in the HRQoL of cancer patients and survivors, further research is required to support our findings.

## Supplementary Information

Supplementary Information.
