# Supplementary material for: Effects of high-intensity training on the quality of life of cancer patients and survivors: a systematic review with meta-analysis
Source: Sci Rep. 2021 Jul 23;11:15089. doi: 10.1038/s41598-021-94476-y (PMC8302720; doi:10.1038/s41598-021-94476-y)

Effects of high-intensity training on the quality of life of cancer patients and survivors: a systematic review with meta-analysis

Lavín-Pérez, Ana Myriam; Collado-Mateo, Daniel; Mayo, Xian; Liguori, Gary; Humphreys, Liam; Copeland, Robert James; Jiménez, Alfonso

SUPPLEMENTARY TABLE S1. Additional characteristics from the meta-analysis participants.

Study	Group	Stage (%)	Physical activity level
Egegaard et al. (2019)	CG	IIIa (28.6%) IIIb (57.1%) IV (14.3%)	NR
	HIEG	IIIa (37.5%) IIIb (50.0%) IV (12.5%)	NR
Mijwel et al. (2018)	CG	I-IIa	MVPA: 8.5 % of wear time SED: 66.6 % of wear time
	HIEG-R		MVPA: 9.6 % of wear time SED: 63.7 % of wear time
	HIEG-A		MVPA: 8.3 ± 2.8 % of wear time SED: 65.6 ± 6.2 % of wear time
Adams et al. (2018)	CG	NR	Aerobic 25% Resistance 35.7%
	HIEG	NR	Aerobic 45.7% Resistance 20%
Van Waart et al. (2017)	CG	II (13%) III (75%) IV (13%)	Not following a formal exercise program (sedentary and active)
	HIEG	II (14%) III (86%) IV (0%)	
	LIEG-H	II (13%) III (75%) IV (13%)	
Persoon et al. (2017)	CG	NR	Sports history (66%)
	HIEG	NR	Sports history (61%)
Mijwel et al. (2017)	CG	I-IIa	MVPA: 68 min/day SED: 548 min/day
	HIEG-R		MVPA: 79. min/day SED:530min/day
	HIEG-A		MVPA: 71 min/day SED: 544. min/day
Toohey et al. (2016)	HIEG	NR	NR
	MIEG	NR	NR
Schmitt et al. (2016)	HIEG	NR	NR
	LIEG	NR	NR
Dunne et al. (2016)	CG	NR	NR
	HIEG	NR	NR
Kampshoff et al. (2015)	CG	I-II (68%) II-IV (32%)	Sport history (54%) Exercise during chemotherapy (11%)
	HIEG	I-II (75%) II-IV (25%)	Sport history (50%) Exercise during chemotherapy (23%)
	LMIEG	I-II (60%) II-IV (40%)	Sport history (65%) Exercise during chemotherapy (22%)

Study	Group	Stage (%)	Physical activity level
Martin et al. (2015) c)	CG	I (3.5%) II (54.28%) III (34.28%)	Total Activity 51.1% Moderate 4.7% Vigorous 2.6%
	HIEG	I (11.1%) II (62.96%) III (25.92%)	Total Activity 32.5.8% Moderate 1.9% Vigorous 0.8%
	LIEG	I (20%) II (68%) III (12%)	Total Activity 30% Moderate 2.7% Vigorous 1.4%
Martin et al. (2015) a)	CG	I (42%) II (50%) III (8%)	Total Activity 35.3% Moderate 3.1% Vigorous 1.6%
	HIEG	I (46%) II (54%)	Total Activity 20% Moderate 1.5% Vigorous 0.8 %
	LIEG	I (42%) II (50%) III (8%)	Total Activity 32.2% Moderate 3.4% Vigorous 0.8%
Van Waart et al. (2015)	CG	I (6%) II (48%) III (46%)	Not following a formal exercise program (sedentary and active)
	HIEG	I (7%) II (42%) III (51%)	
	LIEG-H	I (3%) II (52%) III (45%)	
Møller et al. (2015)	CG	NR	<150 min moderate activity/week (42.85%) >150 min/week (57.14%) <2×20 min strenuous activity / week (100%)
	HIEG	NR	<150 min moderate activity/week (42.85%) >150 min/ week (57.14%) <2×20 min strenuous activity / week (100%)
	LIEG	NR	<150 min moderate activity/week (26.66%) >150 min/week (78.57%) <2×20 min strenuous activity / week (100%)
Edvardsen et al. (2015)	CG	I (48%) II (39%) III (7%) V (3%)	NR
	HIEG	I (60%) II (23%) III (17%)	NR
Cormie et al. (2013)	CG	I (31.6) II (47.3%) III (21.1%)	NR
	HIEG	I (9.1%) II (81.8%) III (9.1%)	NR
	LIEG	I (23.8%) II (47.6%) III (28.6%)	NR
Andersen et al. (2013)	CG	NR	Sedentary (16.3%) For pleasure (25%) Regular exercise (61.5%) Intese exercise (5.8%)
	HIEG	NR	Sedentary (17.3%) For pleasure (56.7%) Regular exercise (23.1%) Intese exercise (2.9%)
Hwang et al. (2012)	CG	II A (9.1%) IV (90.9%)	NR
	HIEG	II A (7.7%) II B (15.4%) IV (76.9%)	NR
Adamsen et al. (2009)	CG	NR	Sedentary (19.8%) For pleasure (38.1%) Regular exercise (37.3%) Intense activity (4.8%)
	HIEG	NR	Sedentary (17.4%) For pleasure (56.8%) Regular (22.7%) Intense physical activity (3.0%)

CG: (inactive) control group: HIEG: high-intensity exercise group; HIEG-R: high-intensity exercise group with resistance component; HIEG-A: high-intensity exercise group with aerobic component; MIEG: moderate intensity exercise group; LIEG: low intensity exercise group; LMIEG: low to intensity exercise group; MVPA: moderate to vigorous physical activity; NR: not reported

SUPPLEMENTARY TABLE S2. Characteristics of the participants included only in the systematic-review.

Study	Design	Group	Sample size (% of females)	Age (SD)	Cancer type (%)	Stage (%)	Treatment	Timing	BMI	Physical activity level
Pereira et al. (2020)	Randomized Control Trial	CG	n=66 (100%)	53 (7)	Breast cancer	II	Surgery (14%) Chemotherapy (60%) Radiation therapy (70%) Hormone therapy (43%)	After	30 (4.2)	Sedentary
		HIEG	n=70 (100%)	55 (5)					31 (3.9)	
		MIEG	n=80 (100%)	51 (4)					29 (5.4)	
Christensen et al. (2018)	Safety and feasibility study	CG	n= 29 (6.89%)	27.8 (5.5)	Adenocarcinoma	0 (3%) I (17%) II (62%) III (17%)	Surgery (79.31%) Chemotherapy (89.65%)	During	27.8 (5.5)	NR
		HIEG	n= 21 (14.28%)	28.4 (5.6)		0 (14%) I (33%) II (38%) III (14%)	Surgery (90.47%) Chemotherapy (95.24%)	During	28.4 (5.6)	NR
Toohey et al. (2018)	Randomized Clinical Trial	CG	n=25(100%)	57(11.5)	Breast (83%), Ovarian (8.5%), Appedix, Cervical (8.5%)	I-II (83%) II-IV (17%)	Surgery (100%) Radiation therapy (83%) Hormone therapy (75%) Chemotherapy, (66%)	After	NR	Sedentary
		HIEG	n= 25(100%)	48 (11.9)	Breast (88%), Cervical (4%), Liver (4%), Esophageal (4%)	I-II (19%) II-IV (21%)	Surgery (88%) Radiation therapy (75%) Hormone therapy (79%) Chemotherapy (82%)	After	NR	Sedentary
		LIEG	n= 25 (100%)	52 (12.4)	Breast (75%), Ovarian (5%), Appedix (5%), Malenoma (5%), Leiomyosarcoma (5%), Unknow primary (5%)	I-II (76%) II-IV (24%)	Surgery (95%) Radiation therapy (62%) Hormone therapy (71%) Chemotherapy (71%)	After	NR	Sedentary
Brunet at al. (2017)	Non-randomized controlled pilot trial	CG	n= 13 (31%)	72	Rectal cancer	≥T2/N+	Chemoradiation (100%)	Before surgery	24.9 (3.9)	NR
		HIEG	n= 22 (36%)	64	Rectal cancer	≥T2/N+	Chemoradiation (100%)	Before surgery	27.4 (5.1)	NR
Waked et al. (2016)	Randomized control trial	CG	n=23 (100%)	48.3(8.9)	Breast cancer	Local (30.4%) Loco-regional (30.4%) Metastasis (39.1%)	Surgery (56.5%) Chemotherapy (34.8%) Radiation (8.7%)	After	NR	NR
		HIEG	n=23 (100%)	49.82 (9.9)		Local (21.7%) Loco-regional (39.1%) Metastasis (39.1%)	Surgery (47.8%) Chemotherapy (39.1%) Radiation (13.0%)	After	NR	NR
Midtgaard et al. (2013)	Randomised controlled trial	CG	n =106 (83%)	46.2 (11.6)	Breast (60.2%) Bowel (5%) Ovaries (2%) Uterus (2%) Testicles (8%)	NR	Surgery (82.07%) Chemotherapy (100%) Radiation (68.86%) Homonotherapy (47.17%)	After	25.1 (3.8)	Sedentary (13%) Walking or cycling for pleasure (60.2%) Regular physical exercise at least 3 h/week (25%)
		HIEG	n= 108 (83.3%)	48.2 (10.1)	Breast (61.1%) Bowel (6%) Ovaries (6%) Uterus (5%) Testicles (3%)	NR	Surgery (77.77%) Chemotherapy (100%) Radiation (60.18%) Homonotherapy (45.37%)	After	24.6 (3.8)	Sedentary (10.2%) Walking or cycling for pleasure (63.9%) Regular physical exercise at least 3 h/week (25.9%)

CG: (inactive) control group; HIEG: high-intensity exercise group; HIEG-R: high-intensity exercise group with resistance training; HIEG-A: high-intensity exercise group with aerobic training; MIEG: moderate intensity exercise group; LIEG: low intensity exercise group; LIEG-H: low intensity exercise group at home; NR: not reported; BMI: Body Mass Index.

SUPPLEMENTARY TABLE S3. Characteristics of the exercise interventions included only in the systematic-review.

Study	Group	Duration	Sessions duration	Weekly frecuency	Setting	Exercise description	Intensity progression and control	Attendance
Pereira et al. (2020)	CG							
	HIEG	36 weeks	70 min	3 times/week	NR	10 min warm-up<br>30 min aerobic: 30 s intervals<br>20 min resistance<br>10 min cool-down	Aerobic: intercalated intervals of 60-80 % (HRmax) and 80-90% (hrmax)<br>Resistance: 60% RM<br>Additional control: RPE	
	MIEG	36 weeks	70 min	3 times/week	NR	10 min warm-up<br>30 min aerobic<br>20 min resistance<br>10 min cool-down	Aerobic: 60-80 % (HRmax)<br>Resistance: 60% RM<br><br>Additional control: RPE	
Christensen et al. (2018)	CG	3-20 weeks						
	HIEG	3-20 weeks	75 min	2 times/week	Centre for Physical Activity Research,	10 min cycle warm-up<br>Aerobic:4x4 min intervals with 3 min active recovery between/ Resistance: four exercises major muscle groups: 3 series of 8-12 repetitions	Intervals: high intensity<br>Recovery: low intensity	68.7%
Toohey et al. (2018)	CG	12 weeks						Compliance: 92%
	HIEG	12 weeks	20-30 min	3 times/week	Supervised	5 min warm-up<br>7x30s intervals with 1min rest<br>5 min cool-down	Intervals ≥85% (HRmax)<br>From 3 intervals in the first session to 7 intervals in week 5<br>Additional control: Blood pressure	
	MIEG	12 weeks	30 min	3 times/week	Supervised	5 min warm-up<br>20 min cycle continuous Aerobic<br>5 min cool-down	≤ 55% predicted maximal heart rate	
Brunet at al. (2017)								
	HIEG	6 weeks	30 min (first 3 sessions) 40 min	3 times/week	Hospital	5 min warm-up<br>3 min braked cycle ergometer intervals with 2 min intervals<br>5 min cool-down	3 min Intervals: moderate intensity (80% of oxygen uptake at lactate threshold)<br>2 min intervals: vigorous intensity (50% of the difference in work rates between peak oxygen uptake and oxygen uptake at lactate threshold)	96% (5.0)
Waked et al. (2016)	CG	8 weeks						
	HIEG	8 weeks	90 min	3 times/week	National Cancer Institute	30 min warm-up: dynamic exercises<br>45 min resistance: 6 exercise 3 series 5-8 rep<br>15 min aerobic cycling	Resistance: 70-100% RM (5.5 METs)<br>Aerobic: 70-250 W 85-95% (HRmax) (15 METs)	
Midtgaard et al. (2013)	CG	12 months						
	HIEG	12 months	90 min	3h/week<br>1 time/week	Supervised	Aerobic:<br>30s-6 min cycle ergometers intervals with a revorery ratio of 1:2 3:1<br>Resistance: 3 series of 8-10 repetitions	Intervals: 30 s (maximun intensity) 6 min 90-95% (HRmáx)<br>Resistance: 70%- 90% (RM)<br>Resistance increased when 12 repetitions could be done	66.6%

CG: (inactive) control group: HIEG: high-intensity exercise group; HIEG-R: high-intensity exercise group with resistance component; HIEG-A: high-intensity exercise group with aerobic component; MIEG: moderate intensity exercise group; W: watios: PP: power peak; RM: repetition maximum; METs: Metabolic equivalent; RPE: rate of perceived exertion; HR: heart rate; CPET: Cardiopulmonary exercise test; NR. Not reported.

SUPPLEMENTARY TABLE S4. HRQoL outcomes not included in the meta-analysis because not having enough data to compare.

	Study	Group	Variable	Mean pre	SD pre	Mean post	SD post	p value
EORTC QLQ- C30	Pereira et al. (2020)	CG	Global QoL	64.5	12.9	63.3	13.4	
		HIEG		58.6	17	125	11.8	SDBG p=0.002
		MIEG		60.7	16.7	68.7	13.6	SDBG p<0.001
	Mijwel et al (2017)	HIAG	Insomnia	31.85	25.58	27.51	28.92	NSDIG
		HIAG	Appetite loss	24.5	26.7	19.53	24.97	NSDIG
		HIAG	Diarrhoea	13.15	22.87	17.74	26.42	NSDIG
		CG	Financial difficulties,	20.11	31.95	18.67	32.53	NSDIG
		HIRG		21.73	31.75	25.45	34.71	SDIG p<0.05
		HIAG		16.54	26.85	23.19	33.54	
	Persoon et al (2017)	CG	Disease symptomsd	19.8	14.6	18.8	17.1	NSDIG ;NSDBG
		EG		16.6	13.1	18.7	12.8	NSDIG;NSDBG
		CG	Side effects	21.9	11.8	12.1	8.9	NSDIG;NSDBG
		EG		21.5	9.7	10.6	8.1	NSDIG;NSDBG
	Schmitt et al. (2016)	HIEG	Insomnia	48.8	29.4	38.5	26.9	SDIG p=0.02 NSDBG
		MIEG		61.5	26.9	43.5	25.2	SDIG p=0.01 NSDBG
		HIEG	Appetite loss	7.7	20	2.5	9.2	NSDIG ; NSDBG
		MIEG		20.5	25.6	7.6	14.5	SDIG p=0.04 NSDBG
		HIEG	Diarrhoea	5.1	12.4	5.1	12.4	NSDIG
		MIEG		33.3	33.4	15.3	29.2	NSDBG
		HIEG	Financial difficulties	28.2	42.7	25.6	33.8	NSDIG NSDBG
		MIEG		38.5	35.7	40.5	38.1	NSDIG NSDBG
	Moller et al (2015) (a)	CG	Financial difficulties	30.3	34.82	25.93	40.06	NR
		HIEG		15.15	22.92	14.81	29.4	NR
		LIEG		30.3	34.82	25.93	40.06	NR
	Moller et al (2015) (b)	CG	Financial difficulties	0	0	0	0	NR
		HIEG		41.67	50	25	31.91	NR
		LIEG		0	0	11.11	19.25	NR
	Adamsen (2009)	CG	Financial difficulties	14.3	27.7	13	25.9	NSDBG
		EG		11.1	21.5	10.8	19.3	NSDBG
SF 36	Dunne (2016)	CG	Overall QoL	71	200	71	220	NSDIG
		EG		65	230	77	180	SDIG p=0.002 SDBG p=0.028
	Adamsen (2009)	CG	Mental component	46.9	10.2	47.3	10	
		HIEG		44.2	8.4	47.4	6.7	SDBG p=0.004
	Adamsen (2009)	CG	Physical component	44.3	8.3	45.1	8.5	
		HIEG		46.5	9.7	50.5	9.4	SDBG p=0.02
	Moller (2015) (a)	CG	Role emotional	70.5	19.3	66.2	20.6	NR
		HIEG		76	21.6	75.1	20.4	NR
		LMIEG		69.7	43.35	70.37	42.31	NR
	Moller (2015) Prostate cacer	CG	Role emotional	66.67	33.33	16.67	23.57	NR
		HIEG		11.11	19.25	33.33	47.14	NR
		LMIEG		0	0	22.22	19.25	NR
FACT G	Andersen (2013)	CG	General score	78.84	13.97	81.45	13.33	
		HIEG		81.14	14.51	84.41	12.88	NSDBG p=0.21
		CG	Physical wellbeing	19.77	5.44	20.59	5.19	
		HIEG		20.64	5.41	21.92	4.59	NSDBG p=0.13
		CG	Functional wellbeing	17.85	5.81	18.67	5.1	
		HIEG		18.93	4.97	19.92	4.88	NSDBG p=0.26
	Toohey et al. (2016)	HIEG	General score	77.63	13.59	89.50	6.82	SDBG p<0.05
		MIEG		81.25	9.45	85.88	7.38	
		HIEG	Physical wellbeing	22.63	5.93	24.75	1.39	NSDIG
		MIEG		21.63	3.16	23.75	2.25	NSDIG
		HIEG		18.13	5.94	22.63	3.34	SDIG p<0.05

		MIEG	Functional wellbeing	20.88	3.94	23.13	3.04	NSDIG
	Martin et al (2015) Breast cancer	CG	General score	83.8	13.5	91.1	11.6	SDIG p=0.044
		HIEG		83.8	13.2	90.9	9.3	
		LIEG		81.8	15.5	84	16.8	
		CG	Physical wellbeing	22.5	4.2	24.7	2.8	SDIG p=0.019
		HIEG		21.7	5.6	24.3	3	
		LIEG		21.1	4.4	22.3	4.5	
		CG	Functional wellbeing	20.9	4.4	23.8	3.7	NR
		HIEG		20.7	4.4	23	3.2	NR
		LIEG		20.8	5	21.4	4.8	NR
		CG	FACT-B	107.6	18.5	116.2	16.9	NR
		HIEG		106.8	17.2	107.6	18.5	SDIG (CG) p=0.014
		LIEG		104.1	19.2	116.9	12.8	
	Martin et al (2015) Prostate cancer	CG	General score	87.3	12.7	90.1	9.6	SDIG p=0.044
		HIEG		84.2	11.8	86	12	
		LIEG		82.5	16.4	85.7	14.5	
		CG	Physical wellbeing	23.7	4.2	24	3.3	SDIG p=0.019
		HIEG		22.9	3.6	23.9	3.9	NR
		LIEG		22.6	4.1	24.1	3.6	NR
		CG	Functional wellbeing	22.7	5.4	24.2	3.1	NR
		HIEG		21.3	4.2	21.9	4.3	NR
		LIEG		19.4	6.5	21	5.2	NR
		CG	FACT-P	122.2	17.4	126.0	14.9	NR
		HIEG		117.2	15.4	120.5	17.1	NSDIG
		LIEG		113.9	22.4	121.5	18.8	
	Egegaard (2019)	CG	Physical well-being	19.2	8.3	22.0	4.3	NSDBG
		HIEG		21.4	6.4	20.9	5.6	
		CG	Functional well-being	17.4	7.1	17.4	4.7	
		HIEG		17.6	5.4	18.0	5.5	NSDBG
		CG	Global score	80.2	16.4	83.8	12.9	
		HIEG		79.1	12.7	81.0	12.5	NSDBG
		CG	FACT- L	99.6	19.0	106.6	14.3	
		HIEG		96.6	14.8	99.0	16.2	NSDBG
FACT -B	Waked (2016)	CG	Global score	80.43	10.90	82.86	12.34	NSDIG
		HIEG		78.13	12.14	112.1	11.08	SDIG p<0.001 SDBG p<0.001

CG: (Inactive) control group: HIEG: High-intensity exercise group; HIEG-A: High-intensity exercise group with aerobic component; MIEG: moderate intensity exercise group; SDIG: Significant difference inside group; SDBG: Significant difference between groups; NSDIG: Not significant difference inside group; NSDBG: Not significant difference between groups, NR. Not reported.

SUPPLEMENTARY TABLE S5. HRQoL outcomes not included in the meta-analysis because not reporting the necessary statistic data.

	Study	Group	Outcome mesure	Statistic results	p value
EORTC-QLQ-C30	Midtgaard (2013)	CG	Global quality of life	Mean before 67.16 CI: 62.65 - 71.52<br>Mean after: 77.44 CI:72.74 - 81.82	SDIG p<0.001
		HIEG		Mean before 67.21 CI: 62.70 - 71.56<br>Mean after: 81.71 CI: 77.16 - 85.85	SDIG p<0.001NSDBG
		CG	Physical functioning	Mean before 67.21 CI: 86.85 - 91.71<br>Mean after: 81.71 CI: 91.19 - 95.61	SDIG p<0.001
		HIEG		Mean before 90.97 CI: 88.54 - 93.13<br>Mean after: 95.94 CI: 93.97.57	SDIG p<0.001 NSDBG
		CG	Role functioning	Mean before 82.51 CI: 77.05 - 87.37<br>Mean after: 90.93 CI: 85.87 - 94.96	SDIG p<0.001
		HIEG		Mean before 79.82 CI: 74.10 - 84.99<br>Mean after: 95.35 CI: 91.24 - 98.20	SDIG p<0.01 NSDBG
		CG	Emotional functioning	Mean before 84.13 CI: 76.96 - 85.17<br>Mean after: 88.59 CI: 84.47 - 92.15	SDIG p<0.01
		HIEG		Mean before 81.24 CI: 80.09 - 87.79<br>Mean after: 77.44 CI: 86.55 - 93.91	SDIG p<0.001 NSDBG
		CG	Cognitive functioning	Mean before 85.43 CI: 80.99 - 89.38<br>Mean after: 90.55 CI: 78.65 - 88.58	NSDIG
		HIEG		Mean before 89.14 CI: 85.17 - 92.56	SDIG p<0.05 NSDBG

				Mean after: 93.29 CI: 80.99 - 89.38	
		CG	Social functioning	Mean before 86.84 CI: 82.20 -90.88<br>Mean after: 94.65 CI: 90.77- 97.51	SDIG p<0.001
		HIEG		Mean before 86.55 CI: 81.88 - 90.63<br>Mean after: 96.3 CI: 92.90 - 98.71	SDIG p<0.01 NSDBG
		CG	Fatigue	Mean before: 34.56 CI: 29.46 - 39.85<br>Mean after: 22.68 CI: 17.62 - 28.18	SDIG p<0.001
		HIEG		Mean before:38.50 CI: 33.26 - 43.87<br>Mean after: 21.88 CI: 16.74 - 27.49	SDIG p<0.01 NSDBG
		CG	Nausea and vomiting	Mean before 1.03 CI: 0.48 - 1.80<br>Mean after: 0.54 CI: 0.12 - 1.26	NSDIG
		HIEG		Mean before 1.06 CI: 0.49 - 1.83<br>Mean after: 0.19 CI: 0.00 - 0.71	SDIG p<0.05 NSDBG
		CG	Pain	Mean before 11.16 CI: 7.53 - 15.40<br>Mean after: 10.38 CI: 6.41 - 15.19	NSDIG
		HIEG		Mean before: 10 CI: 6.58 - 14.05<br>Mean after: 6.4 CI: 3.24 - 10.54	NSDIG; NSDBG
		CG	Dyspnoea	Mean before 8.51 CI: 5.61 - 11.93<br>Mean after: 2.01 CI: 0.57 - 4.30	SDIG p<0.001 NSDBG
		HIEG		Mean before 4.2 CI: 2.23 - 6.77<br>Mean after: 1.57 CI: 0.32 - 3.75	SDIG p<0.05
		CG	Diarrhoea	Mean before 4.83 CI: 2.45 - 7.97<br>Mean after: 2.06 CI: 0.45 -4.81	NSDIG
		HIEG		Mean before: 3.43 CI: 1.47 - 6.17<br>Mean after: 0.83 CI: 0.01- 2.89	SDIG p<0.05 NSDBG
		CG	Constipation	Mean before 2.22 CI: 0.91 - 4.09<br>Mean after: 0.92 CI: 0.11 - 2.51	NSDIG
		HIEG		Mean before 2.98 CI: 1.42 - 5.09<br>Mean after: 0.45 CI: 0.00 - 1.73	SDIG p<0.01 NSDBG
		CG	Appetite loss	Mean before: 0.93 CI: 0.29 - 1.94<br>Mean after: 0.80 CI: 0.15 - 1.97	NSDIG
		HIEG		Mean before: 1.75 CI: 0.80 - 3.06<br>Mean after: 0.03 CI: 1.33 - 0.49	SDIG p<0.001 NSDBG
		CG	Insomnia	Mean before 23.82 CI: 16.72 - 32.54<br>Mean after: 18.02 CI: 11.10 - 26.20	SDIG p<0.001 NSDBG
		HIEG		Mean before 22.07 CI: 15.36 - 29.62<br>Mean after: 18.65 CI: 11.39 - 27.21	NSDIG
		CG	Financial difficulties	Mean before 9.84 CI: 5.35 - 15.49<br>Mean after: 8.79 CI: 4.17 - 14.90	NSDIG
		HIEG		Mean before 4.55 CI: 1.67 - 8.75<br>Mean after: 0.98 CI: 0.00 - 3.85	SDIG p<0.05 NSDBG
SF-36	Midtgaard 2013	CG	Psysical functioning	Mean before: 87.86 CI: 85.10 - 90.38<br>Mean after: 93.71 CI: 91.37 - 95.70	SDIG p<0.01
		HIEG		Mean before 4.55 CI: 1.67 - 8.75<br>Mean after: 95.44 CI: 93.29 - 97.20	SDIG p<0.001 NSDBG
		CG	Role Physical	Mean before 35.16 CI: 24.87 - 46.21<br>Mean after: 73.80 CI: 61.72 - 84.28	SDIG p<0.01
		HIEG		Mean before 31.71 CI: 21.74 - 42.61<br>Mean after: 80.03 CI: 68.10 - 89.73	SDIG p<0.001 NSDBG
		CG	Bodily pain	Mean before: 80.43 CI: 75.05 - 85.29<br>Mean after: 84.35 CI: 78.67 - 89.30	NSDIG
		HIEG		Mean before: 83.89 CI: 78.89 - 88.34<br>Mean after: 89.81 CI: 84.85 - 94.01	SDIG p<0.05 NSDBG
		CG	General health perceptions	Mean before: 68.51 - 77.86 CI: 61.08 - 69.61<br>Mean after: 71.01 CI: 66.40 - 75.41	SDIG p<0.001
		HIEG		Mean before: 69.02 CI: 64.77 - 73.11<br>Mean after: 73.32 CI: 68.51 - 77.86	SDIG p<0.001 NSDBG
		CG	Vitality	Mean before: 54.76 CI: 50.39 - 59.10<br>Mean after: 66.54 CI: 61.80 - 71.11	SDIG p<0.001
		HIEG		Mean before: 54.23 CI: 49.83 - 58.59<br>Mean after: 71.81 CI: 67.00 - 76.38	SDIG p<0.001 NSDBG
		CG	Social functioning	Mean before: 61.42 CI: 58.31 - 64.50<br>Mean after: 69.20 CI: 65.80 - 72.49	SDIG p<0.001
		HIEG		Mean before: 64.97 CI: 61.89 - 67.98<br>Mean after: 73.4 CI: 69.98 - 76.81	SDIG p<0.001 NSDBG
		CG	Role emotional	Mean before: 64.49 CI: 53.80 - 74.49<br>Mean after: 69.20 CI: 65.80 - 72.49	SDIG p<0.001
		HIEG		Mean before: 71.46 CI: 61.16 - 80.75<br>Mean after: 93.1 CI: 84.98 - 98.32	SDIG p<0.001 NSDBG
		CG	Mental health	Mean before: 75.26 CI: 72.06 - 78.33	SDIG p<0.05

				Mean after: 80.29 CI: 76.94 - 83.45	
		HIEG		Mean before 74.73 CI: 71.49 - 77.84<br>Mean after: 82.48 CI: 79.07 - 85.65	SDIG p<0.001 NSDBG
		CG	Physical component scale	Mean before: 45.81 CI: 44.33 - 47.29<br>Mean after: 49.38 CI: 47.73 - 51.02	SDIG p<0.001
		HIEG		Mean before: 46.68 CI: 45.19 - 52.26<br>Mean after: 50.50 CI: 48.74 - 52.26	SDIG p<0.001 NSDBG
		CG	Mental component scale	Mean before 44.9873 CI: 43.13 - 46.85<br>Mean after: 48.70 CI: 46.60 - 50.80	SDIG p<0.01
		HIEG		Mean before: 45.3077 CI: 43.43 - 47.19<br>Mean after: 50.71 CI: 48.45 - 52.98	SDIG p<0.001 NSDBG
FACT-G	Toohey et al. (2018)	CG	Physical wellbeing	Mean after: 21.5 Mean difference: -0.3 CI: -1.09-0.51	
		HIEG		Mean after: 25 Mean difference: 0.89 CI: 0.30-1.48	SDBG (CG) p=0.02
		MIEG		Mean after: 22.48 Mean difference: 0.61 CI: -0.001-1.23	SDBG (CG) p=0.02
		CG	Social wellbeing	Mean after: 22.33 Mean difference: 0.2 CI: -0.85-0.75	
		HIEG		Mean after: 22.04 Mean difference: 0.35 CI: -0.22-0.92	NSDBG (CG) p=0.057<br>NSDBG (MIEG) p=0.057
		MIEG		Mean after: 22.04 Mean difference: 0.2 CI: -0.40-0.80	
		CG	Emotional wellbeing	Mean after: 17.92 Mean difference: -0.11 CI: -0.92-0.69	
		HIEG		Mean after: 19.42 Mean difference: 1.04 CI: 0.43-1.64	SDBG (CG) p<0.01<br>SDBG (MIEG) p<0.01
		MIEG		Mean after: 17.57 Mean difference: 0.15 CI: -0.45-0.76	
		CG	Functional wellbeing	Mean after: 19.58 Mean difference: -0.02 CI: -0.82-0.78	
		HIEG		Mean after: 23.17 Mean difference: 0.96 CI: 0.37-1.56	SDBG (CG) p=0.02<br>SDBG (MIEG) p=0.02
		MIEG		Mean after: 21.29 Mean difference: 0.64 CI: 0.02-1.26	
		CG	Overall quality of life	Mean difference: -0.15 CI: -0.95-0.65	
		HIEG		Mean difference: 1.11 CI: 0.50- 1.72	SDBG (CG) p<0.01<br>SDBG (MIEG) p<0.01
		MIEG		Mean difference: 0.57 CI: -0.00-1.20	

CG: (inactive) control group: HIEG: high-intensity exercise group; HIEG-A: high-intensity exercise group with aerobic component; MIEG: moderate intensity exercise group; SDIG: Significant difference inside group; SDBG: Significant difference between groups; NSDIG: not significant difference inside group; NSDBG: not significant difference between groups; NR: not reported; CI: confident interval.

High Intensity Exercise Programmes Effects in Each Health-Related Quality of Life Dimension

Global health status is presented in Figure S1. HIEG presented a higher global health status in comparison with CG (p=0.0002, with SMD of 0.25 and a 95% CI from 0.12 to 0.39). Differences between HIEG and LMEG were not significant (p=0.12). Improvements in Global health status were observed during (p=0.02, with SMD of 0.22 and a 95% ci from 0.03 to 0.40) and after (p=0.003, with SMD of 0.30 and a 95% CI from 0.10 to 0.50) cancer treatment. Regarding the type of interventions, only the following significant results could be reported: 1) only those programmes with resistance component achieved significant results (p=0.0008, with SMD of 0.25 and a 95% CI from 0.10 to 0.39); 2) interventions with a duration of eight weeks or less (p=0.04, with SMD of 0.23 and a 95% CI from 0.01 to 0.45), as well as longer ones (p=0.002, with SMD of 0.27 and a 95% CI from 0.10 to 0.45), are effective; 3) a frequency of 2 days/week (p=0.002, with a SMD of 0.26 and a 95% CI from 0.10 to 0.42) and also 3 days/week (p=0.03, with a SMD of 0.22 and a 95% CI from 0.02 to 0.42); 4) interventions should involve 120 minutes of exercise or more (p=0.03, with SMD of 0.18 and a 95% CI from 0.02, to 0.42); and, 5) the part of the session of high-intensity should be longer than 15 minutes (p=0.001, with a SMD of 0.32 and a 95% CI from 0.13 to 0.51), less than 15 minutes was reported as p=0.05.

Supplementary Figure S1. Meta-analysis results of the Global health dimension.

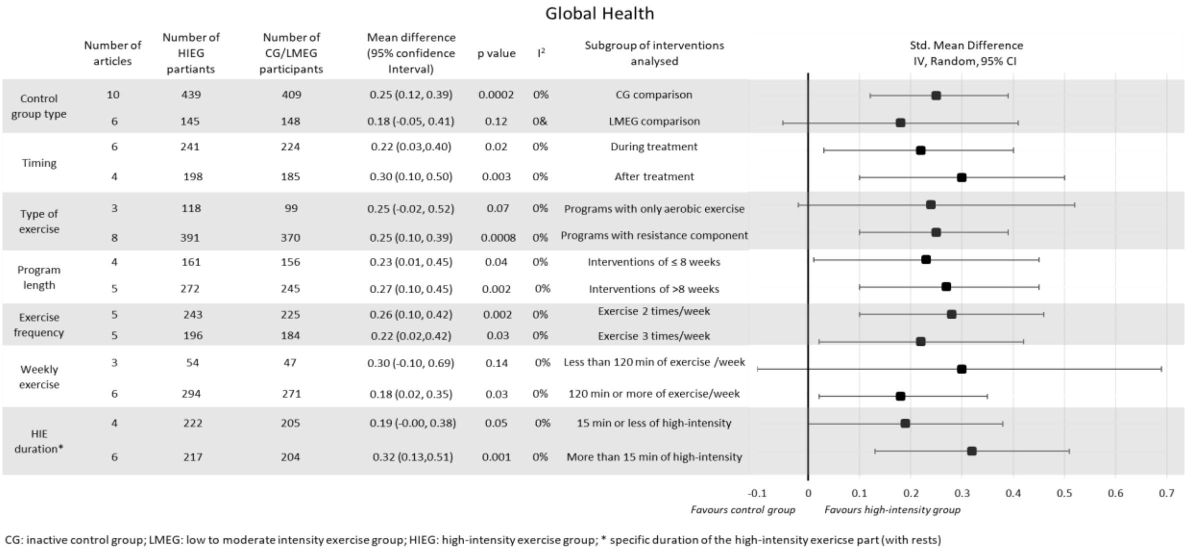

Functional scale

Regarding physical functioning (Figure S2), HIEG significantly improved the physical component when compared to the CG (p=0.0009, with SMD of 0.30 and a 95% CI from 0.12 to 0.49) Differences between HIEG and LMEG were not significant (p=0.91). Concerning cancer treatment timing, increases were only reached in programmes developed during treatment (p=0.0005, with SMD of 0.42 and a 95% CI from 0.18 to 0.66) but not after treatment (p=0.22). For type of exercise, programmes with any kind of resistance training increased physical functioning significantly (p=0.0006, with SMD of 0.34 and a 95% CI from 0. 15 to 0.53). In contrast, interventions with only cardiovascular training did not improve physical function (p=0.17). The significant physical functioning improvements were dependent on the training duration: interventions of 8 weeks or less (p=0.04, with SMD of 0.04 and a 95% CI from 0.01 to 0.45), programmes with a frequency of 2 days/week (p=0.005, with SMD of 0.37 and a 95%CI from 0.11 to 0.62) and at least 120 minutes of exercise/week (p=0.006, with SMD of 0.24 and a 95% CI from 0.07 to 0.40), and work-out with a high-intensity part of the session of 15 minutes or less (p=0.003, with a SMD of 0.29 and a 95% CI from 0.10 to 0.48).

Supplementary Figure S2. Meta-analysis results of the Physical functioning dimension.

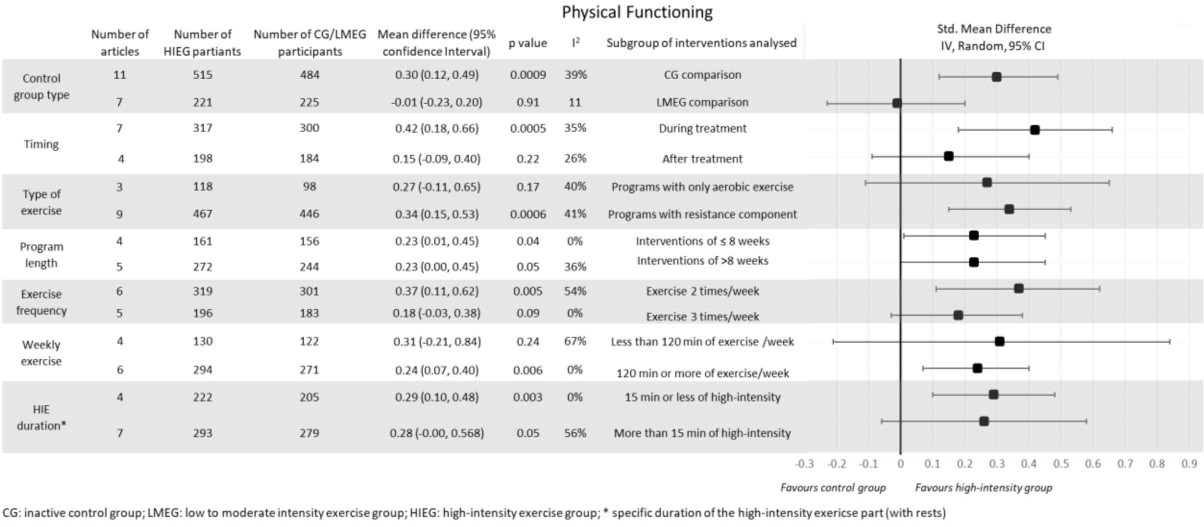

Considering the role functioning, or physical role, the HIEG showed significant enhancements versus CG (p=0.001, with SMD of 0.23 and a 95% CI from 0.09 to 0.37). Differences between HIEG and LMEG were not significant (p=0.15) (Figure S3). As for the CG and HIEG comparison, programmes with interventions implemented during the cancer treatment had significant benefits (p=0.0003, with SMD of 0.35 and a 95% CI from 0.16 to 0.54), however, programmes that were initiated after treatment did not (p=0.37), and only significant improvements were found in programmes of 8 weeks or less (P=0.02, with SMD of 0.26 and a 95% CI from 0.04 to 0.49), with a frequency of 3 times/week (P=0.04, with SMD of 0.21 and a 95% CI from 0.01 to 0.42) and having a high-intensity part of the session of 15 minutes or less (p=0.0004, with SMD of 0.34 and a 95% CI from 0.15 to 0.54).

Supplementary Figure S3. Meta-analysis results of the Role functioning/Physical role dimension.

Role Functioning/ Physical Role

	Number of articles	Number of HIEG partiants	Number of CG/LMEG participants	Mean difference (95% confidence Interval)	p value	I^2	Subgroup of interventions analysed
Control group type	9	433	400	0.23 (0.09, 0.37)	0.001	0%	CG comparison
	5	139	141	0.35 (-0.12, 0.83)	0.15	57%	LMEG comparison
Timing	5	235	216	0.35 (0.16, 0.54)	0.0003	0%	During treatment
	4	198	184	0.09 (-0.11, 0.29)	0.37	0%	After treatment
Program length	4	161	156	0.26 (0.04, 0.49)	0.02	0%	Interventions of ≤ 8 weeks
	5	272	244	0.21 (-0.02, 0.44)	0.07	26%	Interventions of >8 weeks
Exercise frequency	4	237	217	0.26 (-0.00, 0.52)	0.05	44%	Exercise 2 times/week
	5	196	183	0.21 (0.01, 0.42)	0.04	0%	Exercise 3 times/week
HIE duration*	4	222	205	0.34 (0.15, 0.54)	0.0004	0%	15 min or less of high-intensity
	5	211	195	0.11 (-0.08, 0.31)	0.27	0%	More than 15 min of high-intensity

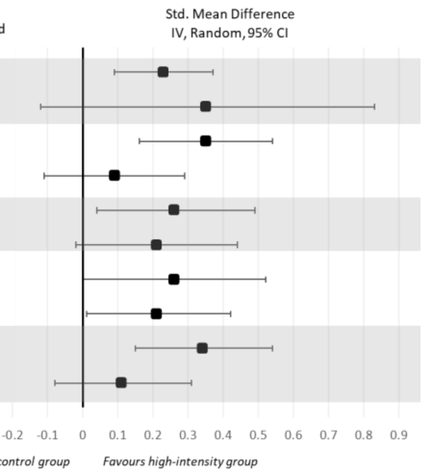

CG: inactive control group; LMEG: low to moderate intensity exercise group; HIEG: high-intensity exercise group; * specific duration of the high-intensity exericse part (with rests)

No significant effects were noted for emotional wellbeing or mental health when comparing HIEG results with CG or with LMEG (Figure S4) as well as in the cancer treatment timing subgroup analysis. However, exercise programmes with only cardiovascular components showed positive significant differences between groups (p=0.007, with SMD of 0.36 and a 95% CI from 0.10 to 0.63) which did not occur when resistance training was used exclusively or when added (p=0.14). No statistical differences were found in the combination of programmes according to the duration characteristics analysis done.

Supplementary Figure S4. Meta-analysis results of the Emotional functioning/ Wellbeing and Mental health

Emotional Functioning/ Wellbeing and Mental Health

	Number of articles	Number of HIEG partiants	Number of CG/LMEG participants	Mean difference (95% confidence Interval)	p value	I^2	Intervention subgroup analysed
Control group type	13	585	560	0.11 (-0.11, 0.22)	0.07	0%	CG comparison
	8	185	191	0.02 (-0.24, 0.29)	0.86	23%	LMEG comparison
Timing	8	349	328	0.09 (-0.6, 0.24)	0.23	0%	During treatment
	6	236	232	0.13 (-0.05, 0.31)	0.16	0%	After treatment
Type of exercise	4	128	103	0.36 (0.10, 0.63)	0.007	0%	Programs with only aerobic exercise
	10	529	517	0.09 (-0.03, 0.21)	0.14	0%	Programs with resistance component
Program length	8	313	316	0.06 (-0.10, 0.21)	0.48	0%	Interventions of ≤ 8 weeks
	5	272	244	0.17 (-0.00,0.34)	0.05	0%	Interventions of >8 weeks
Exercise frequency	4	237	217	0.15 (-0.04, 0.33)	0.12	0%	Exercise 2 times/week
	8	349	338	0.09 (-0.06, 0.24)	0.24	0%	Exercise 3 times/week
Weekly exercise	3	56	43	0.29 (-0.11, 0.69)	0.15	0%	Less than 120 min of exercise /week
	9	438	426	0.05 (-0.09, 0.18)	0.5	0%	120 min or more of exercise/week
HIE duration*	6	336	317	0.08 (-0.07, 0.23)	0.31	0%	15 min or less of high-intensity
	7	249	243	0.15 (-0.03, 0.33)	0.11	0%	More than 15 min of high-intensity

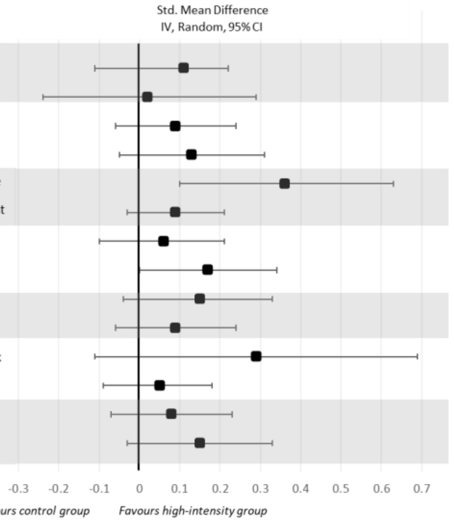

CG: inactive control group; LMEG: low to moderate intensity exercise group; HIEG: high-intensity exercise group; * specific duration of the high-intensity exericse part (with rests)

Statistical improvements were found in cognitive functioning_in the overall analysis of HIEG versus CG (p=0.001, with SMD of 0.22 and a 95% CI from -0.01 to 0.39) but not in the HIEG and LMEG comparison (p=0.07) (Figure S5). When considering intervention duration, statistical increases were found in programmes of more than 8 weeks (p=0.04, with SMD of 0.20 and a 95% CI from 0.01 to 0.40), with an exercise frequency of 2 times/week (p=0.003, with SMD of 0.25 and a 95% CI from 0.08 to 0.42) and both, sessions with a high- intensity part of 15 minutes or less (p=0.04, with SMD of 0.20 and a 95% CI of 0.10 and a 95% CI from 0.01 to 0.39) and sessions with a longer duration of this part (p=0.02, with SMD of 0.23 and a 95% CI from 0.04 to 0.41).

Supplementary Figure S5. Meta-analysis results of the Cognitive functioning dimension.

Cognitive Functioning

	Number of articles	Number of HIEG partiants	Number of CG/LMEG participants	Mean difference (95% confidence Interval)	p value	I^2	Subgroup of interventions analysed
Control group type	8	452	431	0.22 (0.08, 0.35)	0.001	0%	CG comparison
	5	193	197	0.19 (-0.01, 0.39)	0.07	0%	LMEG comparison
Program length	4	161	156	0.15 (-0.07, 0.37)	0.18	0%	Interventions of ≤ 8 weeks
	3	215	198	0.20 (0.01, 0.40)	0.04	0%	Interventions of >8 weeks
Exercise frequency	4	291	275	0.25 (0.08, 0.42)	0.003	0%	Exercise 2 times/week
	4	161	156	0.15 (-0.07, 0.37)	0.18	0%	Exercise 3 times/week
HIE duration*	4	222	205	0.20 (0.01, 0.39)	0.04	0%	15 min or less of high-intensity
	4	230	226	0.23 (0.04, 0.41)	0.02	0%	More than 15 min of high-intensity

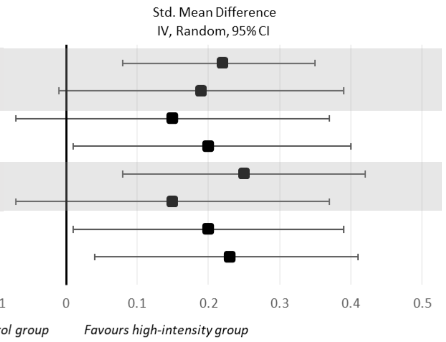

CG: inactive control group; LMEG: low to moderate intensity exercise group; HIEG: high-intensity exercise group; * specific duration of the high-intensity exericse part (with rests)

Regarding social functioning, there were statistically significant improvements in the HIEG compared to the inactive CG (p=0.03, with SMD of 0.12 and a 95% CI from 0.01 to 0.23), but not in contrast to LMEG (p=0.62) (Figure S6). Only those interventions carried out during the cancer treatment had significant increases (p=0.03, with SMD of 0.16 and a 95% CI from 0.01 to 0.32). Moreover, only the interventions involving just aerobic exercise reported significant enhancements in social functioning (p=0.03, with SMD of 0.29 and a 95% CI from 0.03 to0.55). In terms of groups created based on their duration characteristics, there were no statistically significant increases in any of the analyses, except programmes with a high-intensity training component of more than 15 minutes duration (p=0.03, with SMD of 0.17 and a 95% CI from 0.01 to 0.33).

Supplementary Figure S6. Meta-analysis results of the Social functioning dimension.

Social Functioning

	Number of articles	Number of HIEG partiants	Number of CG/LMEG participants	Mean difference (95% confidence Interval)	p value	I^2	Subgroup of interventions analysed
Control group type	14	661	637	0.12 (0.01, 0.23)	0.03	0%	CG comparison
	9	261	268	0.05 (-0.15, 0.24)	0.62	12%	LMEG comparison
Timing	8	354	325	0.16 (0.01, 0.32)	0.03	0%	During treatment
	6	236	232	0.15 (-0.03, 0.34)	0.10	0%	After treatment
Type of exercise	4	130	103	0.29 (0.03, 0.55)	0.03	0%	Programs with only aerobic exercise
	11	605	594	0.11 (-0.00, 0.22)	0.06	0%	Programs with resistance component
Program length	8	313	316	0.08 (-0.07, 0.24)	0.29	0%	Interventions of ≤ 8 weeks
	5	272	244	0.14 (-0.03, 0.31)	0.11	0%	Interventions of >8 weeks
Exercise frequency	5	313	294	0.14 (-0.02, 0.30)	0.10	0%	Exercise 2 times/week
	8	340	338	0.12 (-0.04, 0.27)	0.14	0%	Exercise 3 times/week
Weekly exercise	4	132	120	0.23 (-0.01, 0.48)	0.07	0%	Less than 120 min of exercise/week
	9	438	436	0.07 (-0.07, 0.20)	0.33	11%	120 min or more of exercise/week
HIE duration*	6	336	317	0.08 (-0.08, 0.23)	0.33	0%	15 min or less of high-intensity
	8	325	320	0.17 (0.01, 0.33)	0.03	0%	More than 15 min of high-intensity

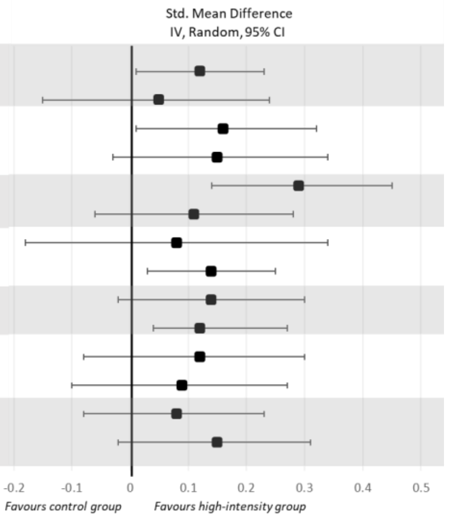

CG: inactive control group; LMEG: low to moderate intensity exercise group; HIEG: high-intensity exercise group; * specific duration of the high-intensity exericse part (with rests)

Symptom scale

As for fatigue or vitality outcomes, statistical improvements were achieved when contrasting HIEG and CG outcomes (p<0.0001, with SMD of -0.32 and a 95% CI from -0.47 to -0.18) but not with the comparison to LMEG (p=0.62) (Figure S7). All the sub-analysis related to the exercise intervention characteristic reported a significant difference between HIEG and CG. In the sub-analysis of all the comparisons related to the intervention's type of exercise and duration, statistical differences between HIEG and CG fatigue were shown. Bodily pain, dyspnea and insomnia, reported significant improvement in the overall comparison of HIEG and CG only (p=0.02, with SMD of -0.18 and a 95% CI from -0.21 to -0.02 in pain analysis; p=0.002 with SMD of -0.34 and a 95% CI from -0.55 to -0.13 in the dyspnoea results and p=0.003, with SMD of -0.29 and a 95% CI from -0.47 to -0.10 in insomnia) (Figures S8 and S9). Thus, in diarrhoea, nausea, constipation and appetite loss significant increases after exercise interventions were not achieved in any of the analyses realized (Figures S9 and S10)

Supplementary Figure S7. Meta-analysis results of the Fatigue/ Vitality dimension.

Fatigue/ Vitality

	Number of articles	Number of HIEG partiants	Number of CG/LMEG participants	Mean difference (95% confidence Interval)	p value	I^2	Subgroup of interventions analysed
Control group type	8	368	340	-0.32 (-0.47, -0.18)	<0.0001	0%	CG comparison
	5	124	118	-0.17 (-0.83, 0.50)	0.62	70%	LMEG comparison
Program length	4	161	156	-0.30 (-0.52, -0.08)	0.008	0%	Interventions of ≤ 8 weeks
	3	131	107	-0.43 (-0.69, -0.17)	0.001	0%	Interventions of >8 weeks
Exercise frequency	3	172	156	-0.32 (-0:54, -0.10)	0.005	0%	Exercise 2 times/week
	5	196	184	-0.33 (-0.53, -0.13)	0.001	0%	Exercise 3 times/week
Weekly exercise	3	124	116	-0.32 (-0.58, -0.06)	0.01	0%	Less than 120 min of exercise/week
	5	244	224	-0.33 (-0.51, -0.14)	0.0005	0%	120 min or more of exercise/week
HIE duration*	4	222	205	-0.34 (-0.53, -0.15)	0.0005	0%	15 min or less of high-intensity
	4	146	135	-0.30 (-0.54, -0.07)	0.01	0%	More than 15 min of high-intensity

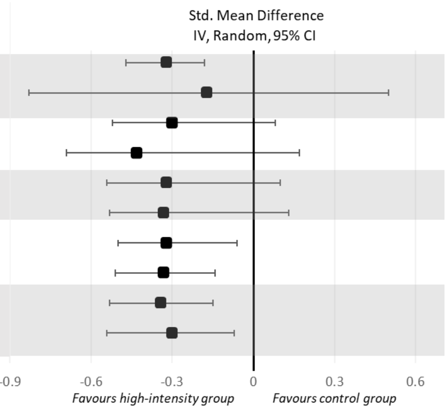

CG: inactive control group; LMEG: low to moderate intensity exercise group; HIEG: high-intensity exercise group; * specific duration of the high-intensity exericse part (with rests)

Supplementary Figure S8. Meta-analysis results of the Bodily Pain dimension.

Bodily Pain

	Number of articles	Number of HIEG partiants	Number of CG/LMEG participants	Mean difference (95% confidence Interval)	p value	I^2	Subgroup of interventions analysed
Control group type	7	355	328	-0.18 (-0.34, -0.02)	0.02	4%	CG comparison
	5	124	123	0.04 (-0.21, 0.29)	0.73	0%	LMEG comparison
Program length	3	148	145	-0.13 (-0.36, 0.10)	0.28	0%	Interventions of ≤ 8 weeks
	3	131	106	-0.05 (-0.36, 0.26)	0.75	27%	Interventions of >8 weeks
Exercise frequency	3	172	156	-0.22 (-0.56, 0.13)	0.23	55%	Exercise 2 times/week
	4	183	172	-0.10 (-0.31, 0.11)	0.36	0%	Exercise 3 times/week
HIE duration*	4	222	205	-0.17 (-0.36, 0.02)	0.09	0%	15 min or less of high-intensity
	3	133	123	-0.09 (-0.55, 0.37)	0.70	65%	More than 15 min of high-intensity

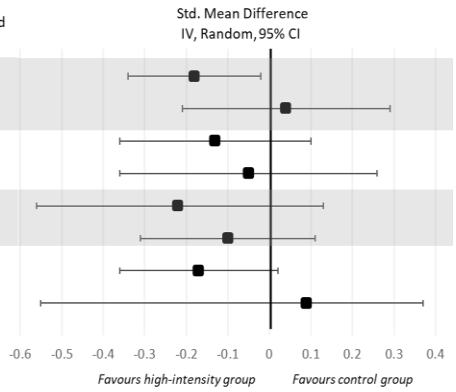

CG: inactive control group; LMEG: low to moderate intensity exercise group; HIEG: high-intensity exercise group; * specific duration of the high-intensity exericse part (with rests)

Supplementary Figure S9. Meta-analysis results of the Dyspnoea, Diarrhoea and Nausea and vomiting dimensions.

Dyspnoea

	Number of articles	Number of HIEG partiants	Number of CG/LMEG participants	Mean difference (95% confidence Interval)	p value	I^2	Subgroup of interventions analysed
Control group type	6	265	251	-0.34 (-0.55, -0.13)	0.002	14%	CG comparison
	4	100	97	-0.18 (-0.46, 0.11)	0.22	0%	LMEG comparison

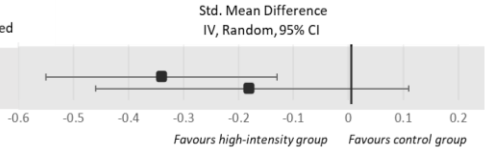

Diarrhoea

	Number of articles	Number of HIEG partiants	Number of CG/LMEG participants	Mean difference (95% confidence Interval)	p value	I^2	Subgroup of interventions analysed
Control group type	4	222	205	0.14 (-0.05, 0.33)	0.16	0%	CG comparison
	4	100	97	-0.11 (-0.44, 0.22)	0.51	0%	LMEG comparison

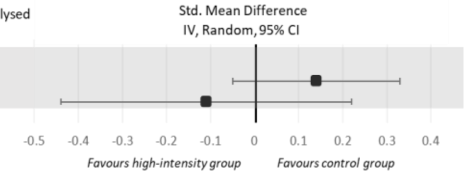

Nausea and vomiting

	Number of articles	Number of HIEG partiants	Number of CG/LMEG participants	Mean difference (95% confidence Interval)	p value	I^2	Subgroup of interventions analysed
Control group type	5	298	282	-0.20 (-0.40, 0.01)	0.06	22%	CG comparison

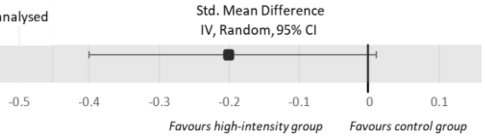

CG: inactive control group; LMEG: low to moderate intensity exercise group; HIEG: high-intensity exercise group.

Supplementary Figure S10. Meta-analysis results of the Insomnia, Constipation and Appetite loss dimensions.

Insomnia

Number of articles	Number of HIEG partiants	Number of CG/LMEG participants	Mean difference (95% confidence Interval)	p value	I²	Subgroup of interventions analysed
4	222	218	-0.29 (-0.47, -0.10)	0.003	0%	Overall comparison
4	100	97	-0.24 (-0.52, 0.04)	0.09	0%	LMEG comparison

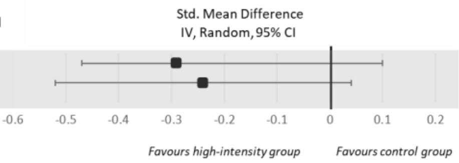

Constipation

Number of articles	Number of HIEG partiants	Number of CG/LMEG participants	Mean difference (95% confidence Interval)	p value	I²	Subgroup of interventions analysed
5	276	254	-0.26 (-0.63, 0.10)	0.15	67%	Overall comparison

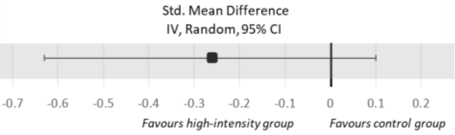

Appetite loss

Number of articles	Number of HIEG partiants	Number of CG/LMEG participants	Mean difference (95% confidence Interval)	p value	I²	Subgroup of interventions analysed
4	222	205	-0.16 (-0.35, 0.03)	0.1	0%	Overall comparison
4	100	97	-0.27 (-0.55, 0.01)	0.06	0%	LMEG comparison

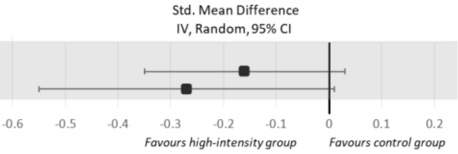

CG: inactive control group; LMEG: low to moderate intensity exercise group; HIEG: high-intensity exercise group.

META-ANALYSIS RESULTS

Supplementary Figure S11: Effects in the global health dimension of the comparisons between high-intensity training group and control group, and high- intensity training group and low- intensity exercise group.

Global health dimension: Comparison between high-intensity group and control group

Study or Subgroup	High-Intensity group Mean	SD	Total	Control group Mean	SD	Total	Weight	Std. Mean Difference IV, Random, 95% CI
Adams 2018	52.2	6.8	35	50.9	8.3	28	7.4%	0.17 [-0.33, 0.67]
Adamsen 2009	67.2	20.3	135	63.3	22.4	134	32.1%	0.18 [-0.06, 0.42]
Cormie 2013	50.4	8.2	22	47	12.3	19	4.8%	0.32 [-0.29, 0.94]
Hwang 2012	78.2	16.1	13	65.2	15.3	11	2.6%	0.80 [-0.04, 1.64]
Kampshoff 2015	82	13.6	91	75.3	15.4	91	21.2%	0.46 [0.16, 0.75]
Mijwel 2017	63.68	19.11	74	59.91	19.03	60	15.8%	0.20 [-0.14, 0.54]
Moller 2015 (a)	63	17.24	9	59.26	20.6	9	2.1%	0.19 [-0.74, 1.11]
Moller 2015 (b)	62.5	34.36	4	45.83	17.68	2	0.6%	0.43 [-1.32, 2.18]
Persoon 2017	75	18.4	50	73.4	18.4	47	11.6%	0.09 [-0.31, 0.48]
Van Wart 2017	73.8	15.5	6	72.6	15.7	8	1.6%	0.07 [-0.99, 1.13]
**Total (95% CI)**			**439**			**409**	**100.0%**	**0.25 [0.12, 0.39]**

Heterogeneity: Tau² = 0.00; Chi² = 4.94, df = 9 (P = 0.84); I² = 0%

Test for overall effect: Z = 3.67 (P = 0.0002)

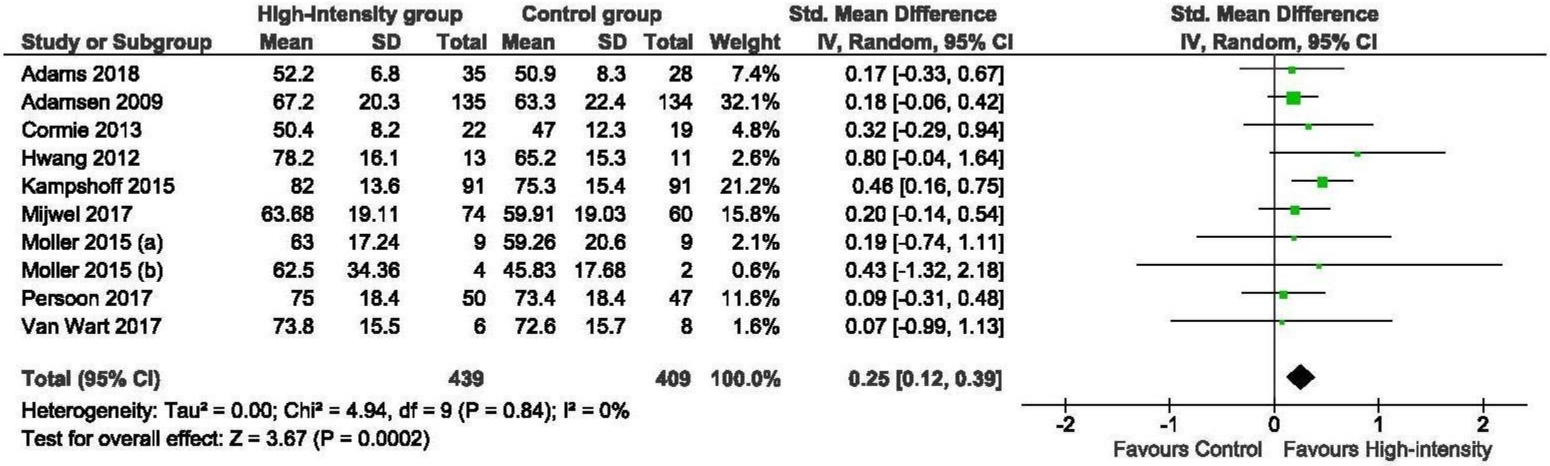

Global health dimension: Comparison between high-intensity group and low-moderate intensity group

Study or Subgroup	High-Intensity group Mean	SD	Total	Active group Mean	SD	Total	Weight	Std. Mean Difference IV, Random, 95% CI
Cormie 2013	50.4	8.2	22	50.8	10	21	14.9%	-0.04 [-0.64, 0.55]
Kampshoff 2015	82	13.6	91	79.7	16.1	95	64.2%	0.15 [-0.13, 0.44]
Moller 2015 (a)	63	17.2	9	60.19	18.99	9	6.2%	0.15 [-0.78, 1.07]
Moller 2015 (b)	62.5	34.36	4	47.22	26.79	3	2.3%	0.41 [-1.12, 1.94]
Schmitt 2016	73	11.2	13	58.3	18.6	13	8.0%	0.93 [0.11, 1.74]
Van Wart 2017	73.8	15.5	6	75	11.8	7	4.5%	-0.08 [-1.17, 1.01]
**Total (95% CI)**			**145**			**148**	**100.0%**	**0.18 [-0.05, 0.41]**

Heterogeneity: Tau² = 0.00; Chi² = 4.10, df = 5 (P = 0.54); I² = 0%

Test for overall effect: Z = 1.54 (P = 0.12)

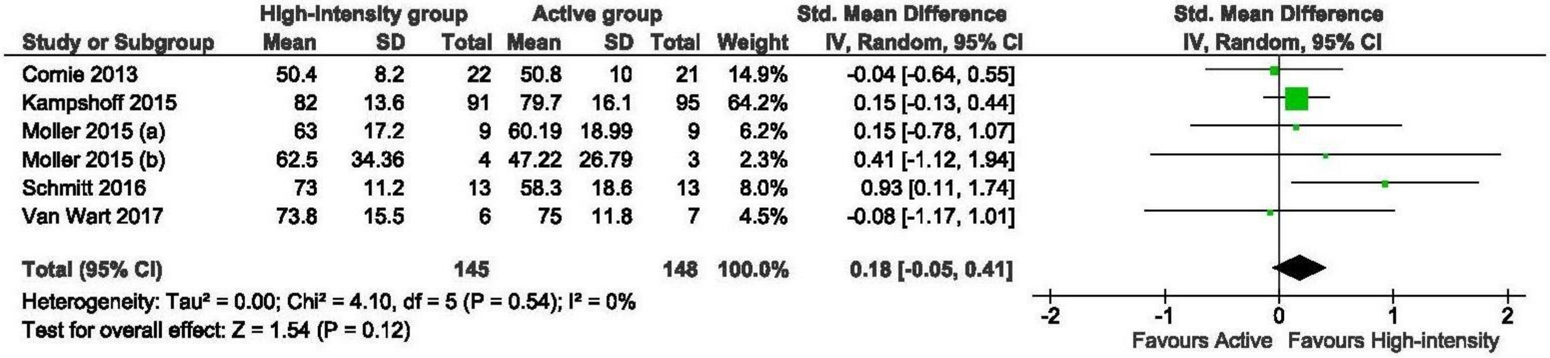

Supplementary Figure S12: Effects of high-intensity training in global health according to the treatment timing.

Global health dimension: During treatment

Study or Subgroup	High-Intensity group Mean	SD	Total	Control group Mean	SD	Total	Weight	Std. Mean Difference IV, Random, 95% CI
Adamsen 2009	67.2	20.3	135	63.3	22.4	134	58.5%	0.18 [-0.06, 0.42]
Hwang 2012	78.2	16.1	13	65.2	15.3	11	4.8%	0.80 [-0.04, 1.64]
Mijwel 2017	63.68	19.11	74	59.91	19.03	60	28.8%	0.20 [-0.14, 0.54]
Moller 2015 (a)	63	17.24	9	59.26	20.6	9	3.9%	0.19 [-0.74, 1.11]
Moller 2015 (b)	62.5	34.36	4	45.83	17.68	2	1.1%	0.43 [-1.32, 2.18]
Van Wart 2017	73.8	15.5	6	72.6	15.7	8	3.0%	0.07 [-0.99, 1.13]
**Total (95% CI)**			**241**			**224**	**100.0%**	**0.22 [0.03, 0.40]**

Heterogeneity: Tau² = 0.00; Chi² = 2.06, df = 5 (P = 0.84); I² = 0%
Test for overall effect: Z = 2.30 (P = 0.02)

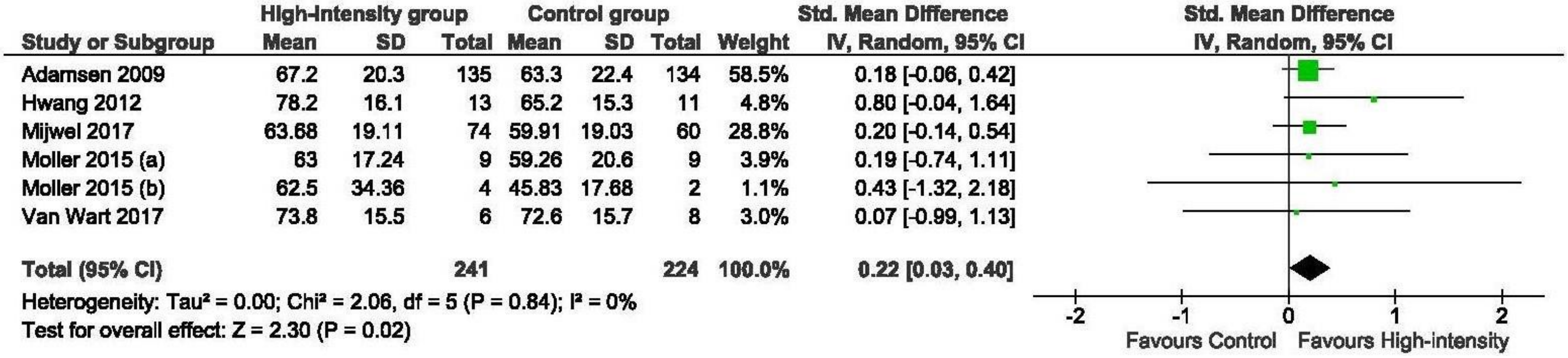

Global health dimension: After treatment

Study or Subgroup	High-Intensity group Mean	SD	Total	Control group Mean	SD	Total	Weight	Std. Mean Difference IV, Random, 95% CI
Adams 2018	52.2	6.8	35	50.9	8.3	28	16.5%	0.17 [-0.33, 0.67]
Cormie 2013	50.4	8.2	22	47	12.3	19	10.7%	0.32 [-0.29, 0.94]
Kampshoff 2015	82	13.6	91	75.3	15.4	91	47.1%	0.46 [0.16, 0.75]
Persoon 2017	75	18.4	50	73.4	18.4	47	25.7%	0.09 [-0.31, 0.48]
**Total (95% CI)**			**198**			**185**	**100.0%**	**0.30 [0.10, 0.50]**

Heterogeneity: Tau² = 0.00; Chi² = 2.49, df = 3 (P = 0.48); I² = 0%
Test for overall effect: Z = 2.92 (P = 0.003)

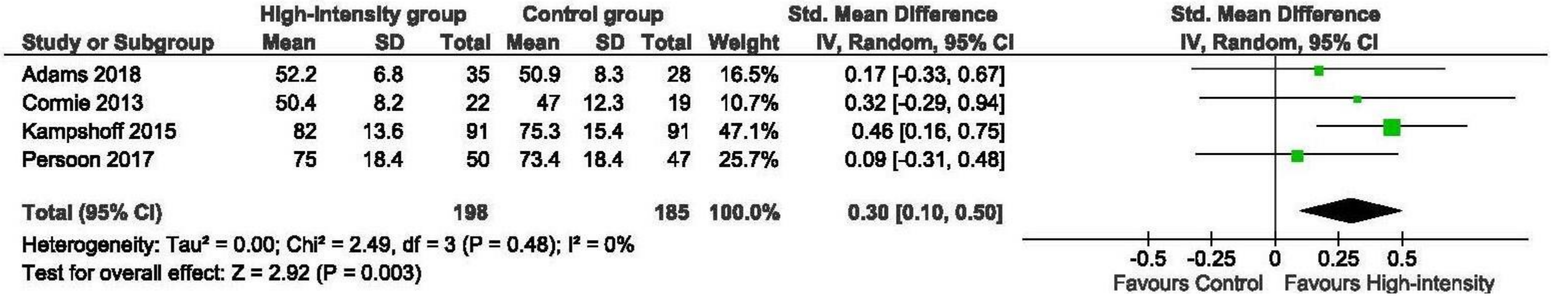

Supplementary Figure S13: Effects of the type pf exercise in global health dimension.

Global health dimension: High-intensity cardiovascular training

Study or Subgroup	High-Intensity group Mean	SD	Total	Control group Mean	SD	Total	Weight	Std. Mean Difference IV, Random, 95% CI
Adams 2018	52.2	6.8	35	50.9	8.3	28	28.7%	0.17 [-0.33, 0.67]
Hwang 2012	78.2	16.1	13	65.2	15.3	11	10.1%	0.80 [-0.04, 1.64]
Mijwel 2017	63.68	19.11	74	59.91	19.03	60	61.2%	0.20 [-0.14, 0.54]
**Total (95% CI)**			**122**			**99**	**100.0%**	**0.25 [-0.02, 0.52]**

Heterogeneity: Tau² = 0.00; Chi² = 1.82, df = 2 (P = 0.40); I² = 0%
Test for overall effect: Z = 1.83 (P = 0.07)

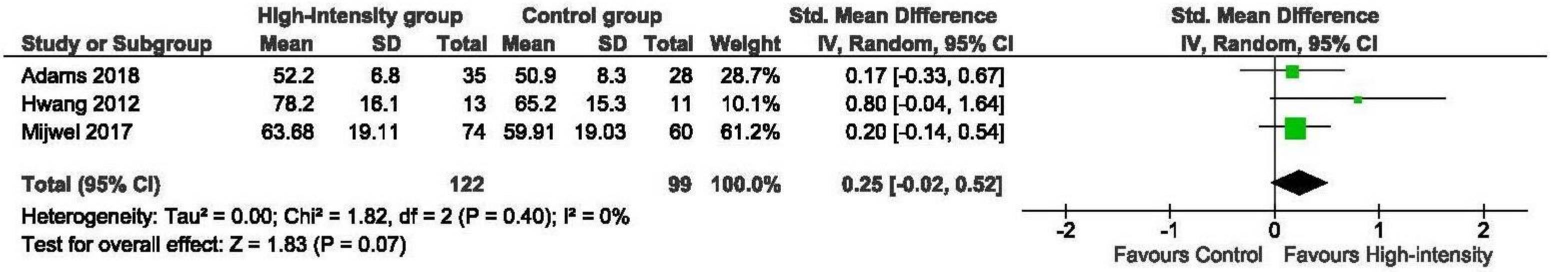

Global health dimension: High-intensity cardiovascular training + Resistance training

Study or Subgroup	High-Intensity group Mean	SD	Total	Control group Mean	SD	Total	Weight	Std. Mean Difference IV, Random, 95% CI
Adamsen 2009	67.2	20.3	135	63.3	22.4	134	35.7%	0.18 [-0.06, 0.42]
Cormie 2013	50.4	8.2	22	47	12.3	19	5.4%	0.32 [-0.29, 0.94]
Kampshoff 2015	82	13.6	91	75.3	15.4	91	23.6%	0.46 [0.16, 0.75]
Mijwel 2017	63.68	19.11	74	59.91	19.03	60	17.6%	0.20 [-0.14, 0.54]
Moller 2015 (a)	63	17.24	9	59.26	20.6	9	2.4%	0.19 [-0.74, 1.11]
Moller 2015 (b)	62.5	34.36	4	45.83	17.68	2	0.7%	0.43 [-1.32, 2.18]
Persoon 2017	75	18.4	50	73.4	18.4	47	12.9%	0.09 [-0.31, 0.48]
Van Wart 2017	73.8	15.5	6	72.6	15.7	8	1.8%	0.07 [-0.99, 1.13]
**Total (95% CI)**			**391**			**370**	**100.0%**	**0.25 [0.10, 0.39]**

Heterogeneity: Tau² = 0.00; Chi² = 3.21, df = 7 (P = 0.86); I² = 0%
Test for overall effect: Z = 3.36 (P = 0.0008)

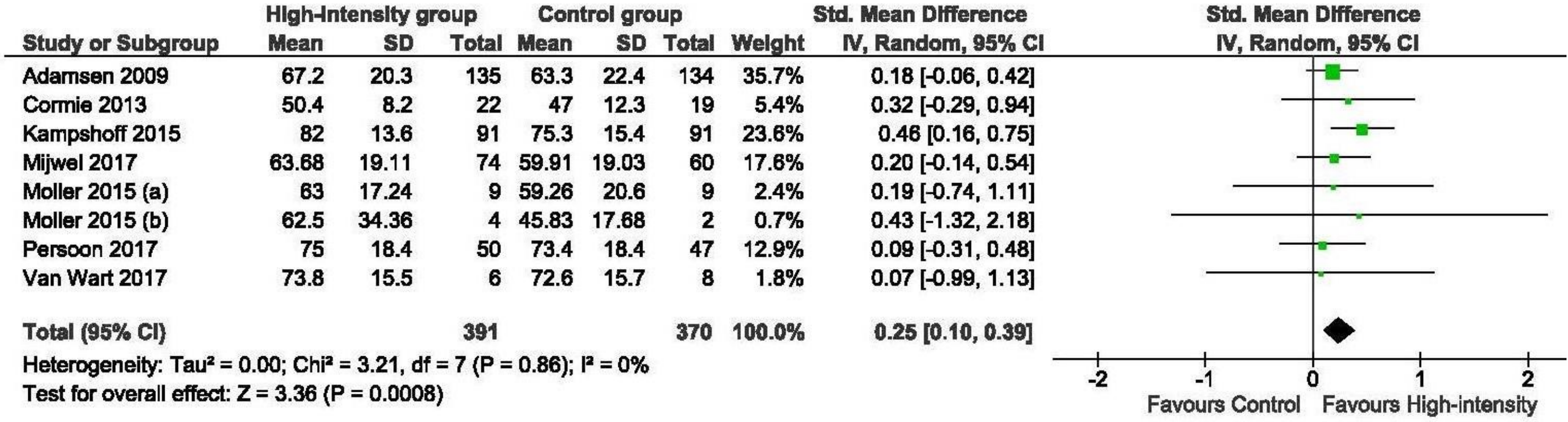

Supplementary Figure S14: Effects of the exercise programs length in global health dimension.

Global health dimension: Eight weeks of exercise program or less

Study or Subgroup	High-intensity group Mean	SD	Total	Control group Mean	SD	Total	Weight	Std. Mean Difference IV, Random, 95% CI
Adamsen 2009	67.2	20.3	135	63.3	22.4	134	85.7%	0.18 [-0.06, 0.42]
Hwang 2012	78.2	16.1	13	65.2	15.3	11	7.0%	0.80 [-0.04, 1.64]
Moller 2015 (a)	63	17.24	9	59.26	20.6	9	5.7%	0.19 [-0.74, 1.11]
Moller 2015 (b)	62.5	34.36	4	45.83	17.68	2	1.6%	0.43 [-1.32, 2.18]
**Total (95% CI)**			**161**			**156**	**100.0%**	**0.23 [0.01, 0.45]**

Heterogeneity: Tau² = 0.00; Chi² = 1.97, df = 3 (P = 0.58); I² = 0%
Test for overall effect: Z = 2.03 (P = 0.04)

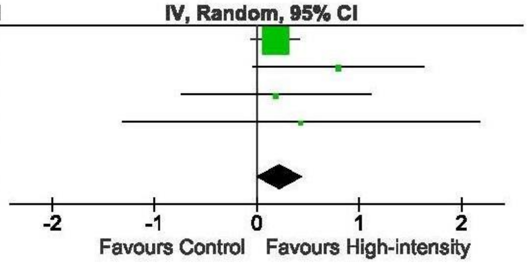

Global health dimension: More than eight weeks of exercise program

Study or Subgroup	High-intensity group Mean	SD	Total	Control group Mean	SD	Total	Weight	Std. Mean Difference IV, Random, 95% CI
Adams 2018	52.2	6.8	35	50.9	8.3	28	12.2%	0.17 [-0.33, 0.67]
Cormie 2013	50.4	8.2	22	47	12.3	19	7.9%	0.32 [-0.29, 0.94]
Kampshoff 2015	82	13.6	91	75.3	15.4	91	34.9%	0.46 [0.16, 0.75]
Mijwel 2017	63.68	19.11	74	59.91	19.03	60	26.0%	0.20 [-0.14, 0.54]
Persoon 2017	75	18.4	50	73.4	18.4	47	19.1%	0.09 [-0.31, 0.48]
**Total (95% CI)**			**272**			**245**	**100.0%**	**0.27 [0.10, 0.45]**

Heterogeneity: Tau² = 0.00; Chi² = 2.76, df = 4 (P = 0.60); I² = 0%
Test for overall effect: Z = 3.09 (P = 0.002)

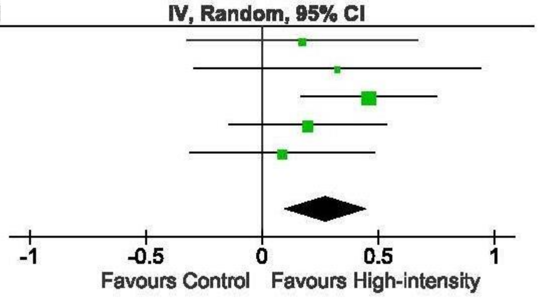

Supplementary Figure S15: Effects of the weekly exercise frequency in global health dimension

Global health dimension: 2 times per week

Study or Subgroup	High-intensity group Mean	SD	Total	Control group Mean	SD	Total	Weight	Std. Mean Difference IV, Random, 95% CI
Adamsen 2009	67.2	20.3	135	63.3	22.4	134	45.0%	0.18 [-0.06, 0.42]
Cormie 2013	50.4	8.2	22	47	12.3	19	6.7%	0.32 [-0.29, 0.94]
Kampshoff 2015	82	13.6	91	75.3	15.4	91	29.7%	0.46 [0.16, 0.75]
Persoon 2017	75	18.4	50	73.4	18.4	47	16.2%	0.09 [-0.31, 0.48]
Van Wart 2017	73.8	15.5	6	72.6	15.7	8	2.3%	0.07 [-0.99, 1.13]
**Total (95% CI)**			**304**			**299**	**100.0%**	**0.26 [0.10, 0.42]**

Heterogeneity: Tau² = 0.00; Chi² = 3.06, df = 4 (P = 0.55); I² = 0%
Test for overall effect: Z = 3.12 (P = 0.002)

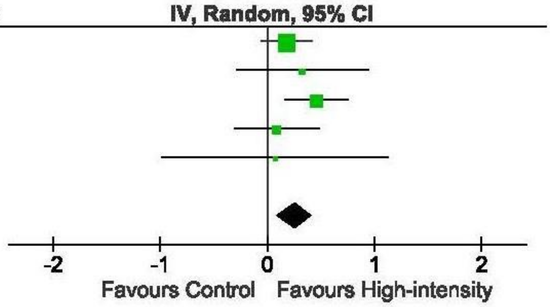

Global health dimension: 3 times per week

Study or Subgroup	High-intensity group Mean	SD	Total	Control group Mean	SD	Total	Weight	Std. Mean Difference IV, Random, 95% CI
Adams 2018	52.2	6.8	35	50.9	8.3	28	16.5%	0.17 [-0.33, 0.67]
Adamsen 2009	67.2	20.3	135	63.3	22.4	134	71.5%	0.18 [-0.06, 0.42]
Hwang 2012	78.2	16.1	13	65.2	15.3	11	5.8%	0.80 [-0.04, 1.64]
Moller 2015 (a)	63	17.24	9	59.26	20.6	9	4.8%	0.19 [-0.74, 1.11]
Moller 2015 (b)	62.5	34.36	4	45.83	17.68	2	1.3%	0.43 [-1.32, 2.18]
**Total (95% CI)**			**196**			**184**	**100.0%**	**0.22 [0.02, 0.42]**

Heterogeneity: Tau² = 0.00; Chi² = 2.01, df = 4 (P = 0.73); I² = 0%
Test for overall effect: Z = 2.12 (P = 0.03)

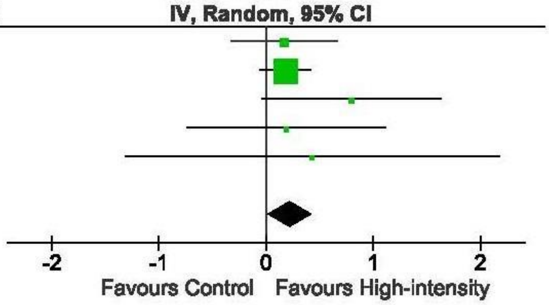

Supplementary Figure S16: Effects of training minutes per week in global health dimension.

Global health dimension: Less than 120 minutes of exercise per weeks

Study or Subgroup	High-intensity group Mean	SD	Total	Control group Mean	SD	Total	Weight	Std. Mean Difference IV, Random, 95% CI
Adams 2018	52.2	6.8	35	50.9	8.3	28	63.6%	0.17 [-0.33, 0.67]
Hwang 2012	78.2	16.1	13	65.2	15.3	11	22.3%	0.80 [-0.04, 1.64]
Van Wart 2017	73.8	15.5	6	72.6	15.7	8	14.1%	0.07 [-0.99, 1.13]
Total (95% CI)			54			47	100.0%	0.30 [-0.10, 0.69]

Heterogeneity: Tau² = 0.00; Chi² = 1.78, df = 2 (P = 0.41); I² = 0%
Test for overall effect: Z = 1.47 (P = 0.14)

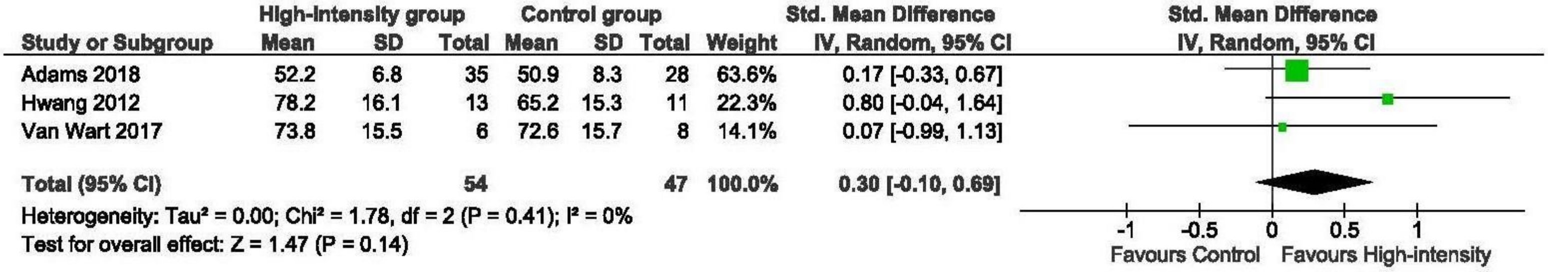

Global health dimension:120 minutes or more or exercise per weeks

Study or Subgroup	High-intensity group Mean	SD	Total	Control group Mean	SD	Total	Weight	Std. Mean Difference IV, Random, 95% CI
Adamsen 2009	67.2	20.3	135	63.3	22.4	134	47.9%	0.18 [-0.06, 0.42]
Cormie 2013	50.4	8.2	22	47	12.3	19	7.2%	0.32 [-0.29, 0.94]
Mijwel 2017	63.68	19.11	74	59.91	19.03	60	23.6%	0.20 [-0.14, 0.54]
Moller 2015 (a)	63	17.24	9	59.26	20.6	9	3.2%	0.19 [-0.74, 1.11]
Moller 2015 (b)	62.5	34.36	4	45.83	17.68	2	0.9%	0.43 [-1.32, 2.18]
Persoon 2017	75	18.4	50	73.4	18.4	47	17.3%	0.09 [-0.31, 0.48]
Total (95% CI)			294			271	100.0%	0.18 [0.02, 0.35]

Heterogeneity: Tau² = 0.00; Chi² = 0.51, df = 5 (P = 0.99); I² = 0%
Test for overall effect: Z = 2.15 (P = 0.03)

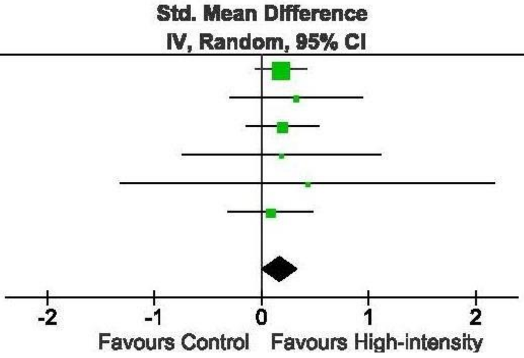

Supplementary Figure S17: Effects of the high-intensity training part duration in global health dimension.

Global health: 15 minutes or les of high-intensity training

Study or Subgroup	High-intensity group Mean	SD	Total	Control group Mean	SD	Total	Weight	Std. Mean Difference IV, Random, 95% CI
Adamsen 2009	67.2	20.3	135	63.3	22.4	134	63.4%	0.18 [-0.06, 0.42]
Mijwel 2017	63.68	19.11	74	59.91	19.03	60	31.2%	0.20 [-0.14, 0.54]
Moller 2015 (a)	63	17.24	9	59.26	20.6	9	4.2%	0.19 [-0.74, 1.11]
Moller 2015 (b)	62.5	34.36	4	45.83	17.68	2	1.2%	0.43 [-1.32, 2.18]
Total (95% CI)			222			205	100.0%	0.19 [-0.00, 0.38]

Heterogeneity: Tau² = 0.00; Chi² = 0.08, df = 3 (P = 0.99); I² = 0%
Test for overall effect: Z = 1.95 (P = 0.05)

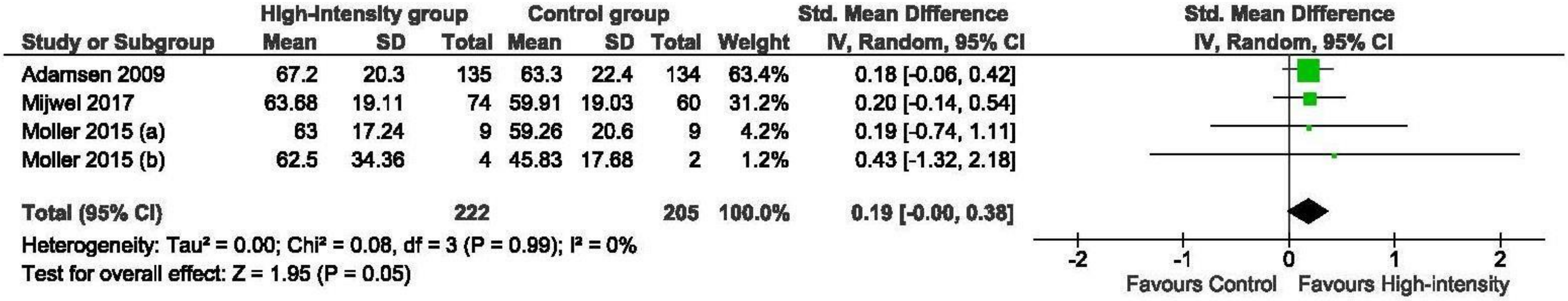

Global health: More than 15 minutes of high-intensity training

Study or Subgroup	High-intensity group Mean	SD	Total	Control group Mean	SD	Total	Weight	Std. Mean Difference IV, Random, 95% CI
Adams 2018	52.2	6.8	35	50.9	8.3	28	15.1%	0.17 [-0.33, 0.67]
Cormie 2013	50.4	8.2	22	47	12.3	19	9.8%	0.32 [-0.29, 0.94]
Hwang 2012	78.2	16.1	13	65.2	15.3	11	5.3%	0.80 [-0.04, 1.64]
Kampshoff 2015	82	13.6	91	75.3	15.4	91	43.0%	0.46 [0.16, 0.75]
Persoon 2017	75	18.4	50	73.4	18.4	47	23.5%	0.09 [-0.31, 0.48]
Van Wart 2017	73.8	15.5	6	72.6	15.7	8	3.3%	0.07 [-0.99, 1.13]
Total (95% CI)			217			204	100.0%	0.32 [0.13, 0.51]

Heterogeneity: Tau² = 0.00; Chi² = 3.98, df = 5 (P = 0.55); I² = 0%
Test for overall effect: Z = 3.25 (P = 0.001)

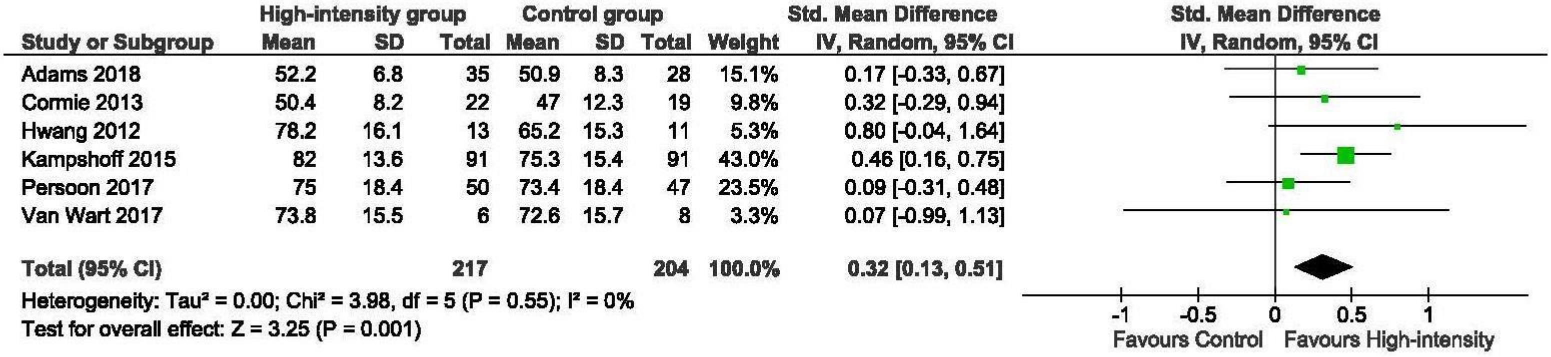

Supplementary Figure S18: Effects in the physical functioning dimension of the comparisons between high-intensity training group and control group, and high-intensity training group and low- intensity exercise group.

Physical functioning dimension: Comparison between high-intensity group and control group.

Study or Subgroup	High-intensity group Mean	SD	Total	Control group Mean	SD	Total	Weight	Std. Mean Difference IV, Random, 95% CI
Adams 2018	54.4	4.4	35	54.8	3.7	27	8.7%	-0.10 [-0.60, 0.41]
Adamsen 2009	89	12.4	135	86.4	14.5	134	18.4%	0.19 [-0.05, 0.43]
Cornie 2013	48.5	7.8	22	45.4	9.7	19	6.5%	0.35 [-0.27, 0.97]
Hwang 2012	92.3	6.6	13	87.9	11.9	11	4.1%	0.45 [-0.36, 1.27]
Kampshoff 2015	88.1	9.5	91	84.1	13.1	91	15.8%	0.35 [0.06, 0.64]
Mijwel 2017	85.88	15.87	74	77.49	19.65	60	13.6%	0.47 [0.13, 0.82]
Moller 2015 (a)	91.11	6.67	9	86.67	6.67	9	3.2%	0.63 [-0.32, 1.59]
Moller 2015 (b)	75	28.48	4	80	0.01	2	1.1%	-0.16 [-1.87, 1.54]
Persoon 2017	83.1	19.1	50	84.1	15.3	47	11.7%	-0.06 [-0.46, 0.34]
Van Wart 2015	80.3	14.1	76	68.1	17.6	76	14.3%	0.76 [0.43, 1.09]
Van Wart 2017	75.2	16.2	6	77.1	15.3	8	2.6%	-0.11 [-1.17, 0.95]
**Total (95% CI)**			**515**			**484**	**100.0%**	**0.30 [0.12, 0.49]**

Heterogeneity: Tau² = 0.03; Chi² = 16.30, df = 10 (P = 0.09); I² = 39%
Test for overall effect: Z = 3.32 (P = 0.0009)

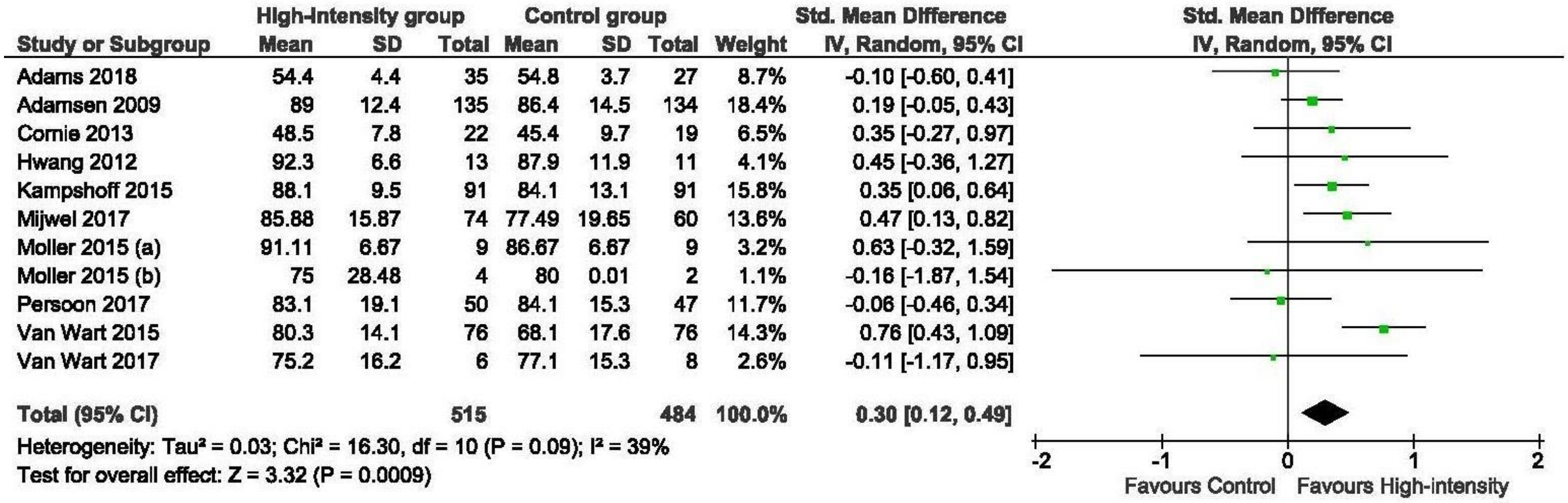

Physical functioning dimension: Comparison between high-intensity group and low-moderate intensity group

Study or Subgroup	High-intensity group Mean	SD	Total	Active group Mean	SD	Total	Weight	Std. Mean Difference IV, Random, 95% CI
Cornie 2013	48.5	7.8	22	49.2	5.8	21	11.6%	-0.10 [-0.70, 0.50]
Kampshoff 2015	88.1	9.5	91	89.6	10.2	95	37.9%	-0.15 [-0.44, 0.14]
Moller 2015 (a)	91.11	6.67	9	90.37	7.54	9	5.2%	0.10 [-0.83, 1.02]
Moller 2015 (b)	75	28.48	4	77.78	13.88	3	2.0%	-0.10 [-1.60, 1.40]
Schmitt 2016	87.2	11	13	81.5	11.3	13	7.1%	0.50 [-0.29, 1.28]
Van Wart 2015	80.3	14.1	76	77.8	17.2	77	33.0%	0.16 [-0.16, 0.48]
Van Wart 2017	75.2	16.2	6	91.4	12	7	3.1%	-1.07 [-2.27, 0.13]
**Total (95% CI)**			**221**			**225**	**100.0%**	**-0.01 [-0.23, 0.20]**

Heterogeneity: Tau² = 0.01; Chi² = 6.78, df = 6 (P = 0.34); I² = 11%
Test for overall effect: Z = 0.11 (P = 0.91)

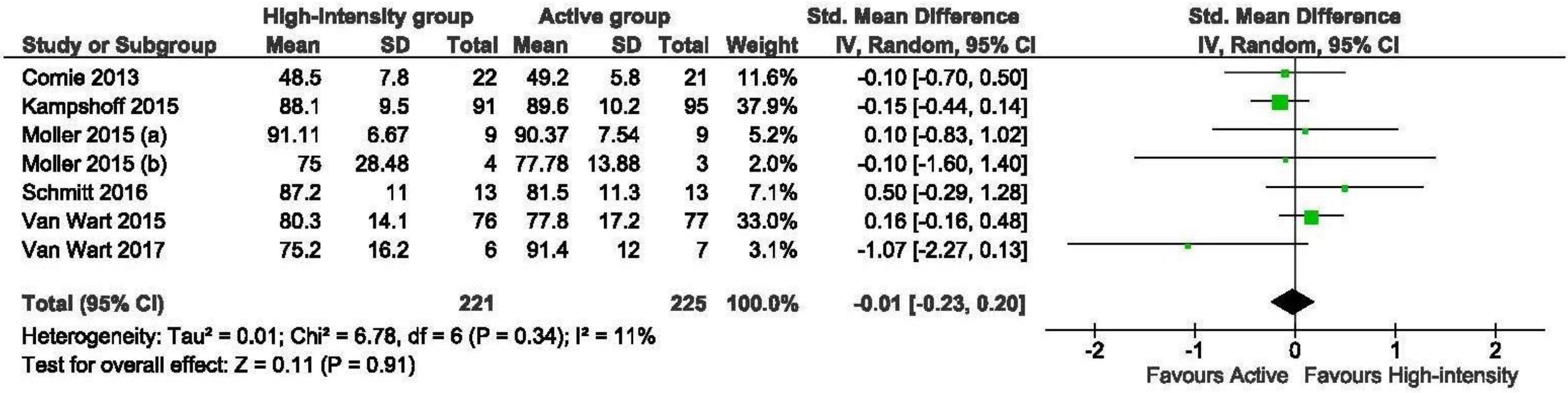

Supplementary Figure S19: Effects of high-intensity training in physical functioning according to the treatment timing

Physical functioning dimension: During treatment

Study or Subgroup	High-intensity Mean	SD						CI
Adamsen 2009	89	12.4	135	86.4	14.5	134	32.1%	0.19 [-0.05, 0.43]
Hwang 2012	92.3	6.6	13	87.9	11.9	11	7.3%	0.45 [-0.36, 1.27]
Mijwel 2017	85.88	15.87	74	77.49	19.65	60	23.8%	0.47 [0.13, 0.82]
Moller 2015 (a)	91.11	6.67	9	86.67	6.67	9	5.5%	0.63 [-0.32, 1.59]
Moller 2015 (b)	75	28.48	4	80	0.01	2	1.9%	-0.16 [-1.87, 1.54]
Van Wart 2015	80.3	14.1	76	68.1	17.6	76	24.9%	0.76 [0.43, 1.09]
Van Wart 2017	75.2	16.2	6	77.1	15.3	8	4.6%	-0.11 [-1.17, 0.95]
**Total (95% CI)**			**317**			**300**	**100.0%**	**0.42 [0.18, 0.66]**

Heterogeneity: Tau² = 0.03; Chi² = 9.24, df = 6 (P = 0.16); I² = 35%
Test for overall effect: Z = 3.47 (P = 0.0005)

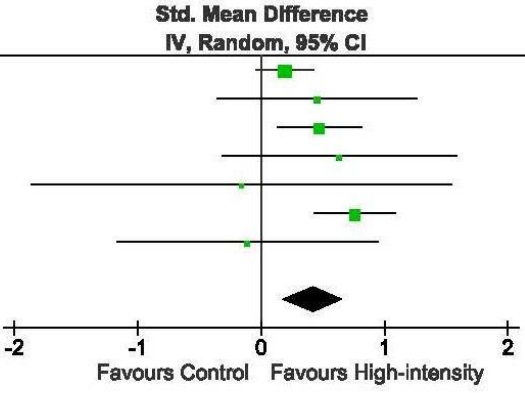

Physical functioning dimension: After treatment

Study or Subgroup	High-intensity group Mean	SD	Total	Control group Mean	SD	Total	Weight	Std. Mean Difference IV, Random, 95% CI
Adams 2018	54.4	4.4	35	54.8	3.7	27	19.1%	-0.10 [-0.60, 0.41]
Cornie 2013	48.5	7.8	22	45.4	9.7	19	13.5%	0.35 [-0.27, 0.97]
Kampshoff 2015	88.1	9.5	91	84.1	13.1	91	40.2%	0.35 [0.06, 0.64]
Persoon 2017	83.1	19.1	50	84.1	15.3	47	27.1%	-0.06 [-0.46, 0.34]
**Total (95% CI)**			**198**			**184**	**100.0%**	**0.15 [-0.09, 0.40]**

Heterogeneity: Tau² = 0.02; Chi² = 4.07, df = 3 (P = 0.25); I² = 26%
Test for overall effect: Z = 1.22 (P = 0.22)

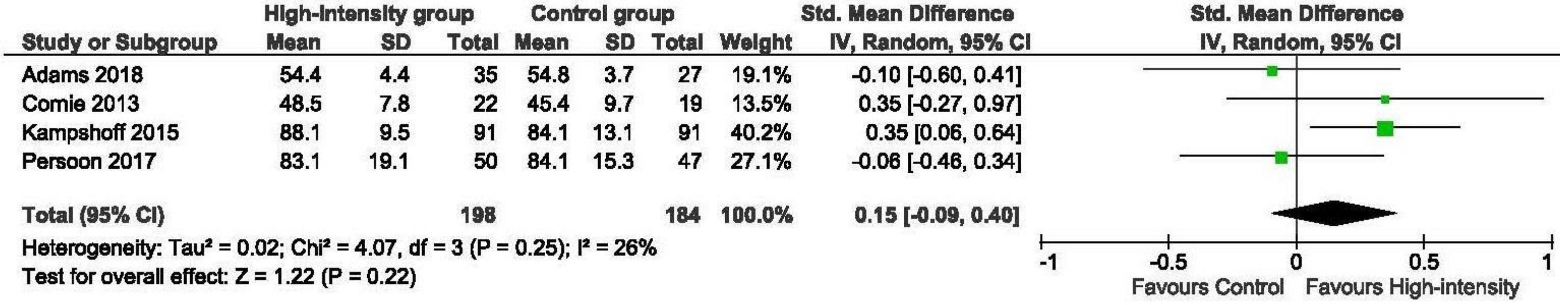

Supplementary Figure S20: Effects of the type of exercise in physical functioning dimension.

Physical functioning: High-intensity cardiovascular training

Study or Subgroup	High-intensity group Mean	SD	Total	Control group Mean	SD	Total	Weight	Std. Mean Difference IV, Random, 95% CI
Adams 2018	54.4	4.4	35	54.8	3.7	27	34.0%	-0.10 [-0.60, 0.41]
Hwang 2012	92.3	6.6	13	87.9	11.9	11	17.3%	0.45 [-0.36, 1.27]
Mijwel 2017	85.37	14.49	70	77.49	19.65	60	48.7%	0.46 [0.11, 0.81]
Total (95% CI)			118			98	100.0%	0.27 [-0.11, 0.65]

Heterogeneity: Tau² = 0.05; Chi² = 3.32, df = 2 (P = 0.19); I² = 40%
Test for overall effect: Z = 1.38 (P = 0.17)

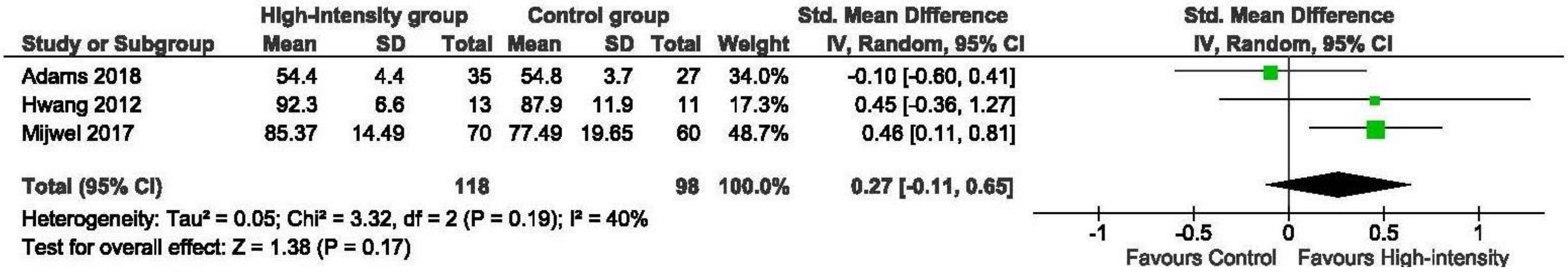

Physical functioning: High-intensity cardiovascular training + Resistance training

Study or Subgroup	High-intensity group Mean	SD	Total	Control group Mean	SD	Total	Weight	Std. Mean Difference IV, Random, 95% CI
Adamsen 2009	89	12.4	135	86.4	14.5	134	21.1%	0.19 [-0.05, 0.43]
Cornie 2013	48.5	7.8	22	45.4	9.7	19	7.4%	0.35 [-0.27, 0.97]
Kampshoff 2015	88.1	9.5	91	84.1	13.1	91	18.2%	0.35 [0.06, 0.64]
Mijwel 2017	85.88	15.87	74	77.49	19.65	60	15.6%	0.47 [0.13, 0.82]
Moller 2015 (a)	91.11	6.67	9	86.67	6.67	9	3.6%	0.63 [-0.32, 1.59]
Moller 2015 (b)	75	28.48	4	80	0.01	2	1.2%	-0.16 [-1.87, 1.54]
Persoon 2017	83.1	19.1	50	84.1	15.3	47	13.4%	-0.06 [-0.46, 0.34]
Van Wart 2015	80.3	14.1	76	68.1	17.6	76	16.4%	0.76 [0.43, 1.09]
Van Wart 2017	75.2	16.2	6	77.1	15.3	8	3.0%	-0.11 [-1.17, 0.95]
Total (95% CI)			467			446	100.0%	0.34 [0.15, 0.53]

Heterogeneity: Tau² = 0.03; Chi² = 13.52, df = 8 (P = 0.10); I² = 41%
Test for overall effect: Z = 3.44 (P = 0.0006)

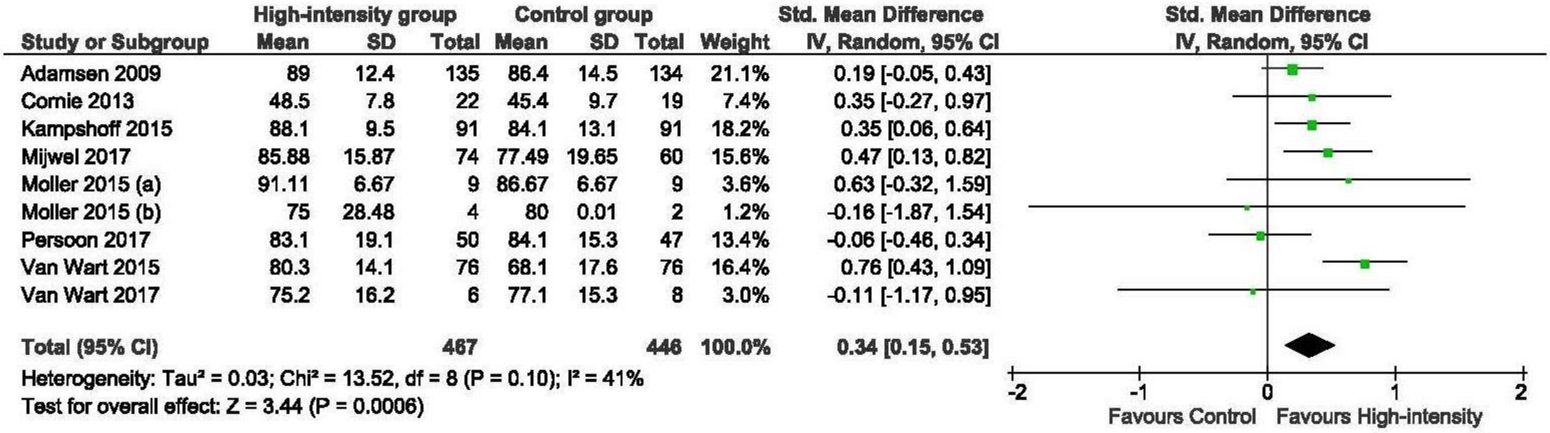

Supplementary Figure S21: Effects of the exercise programs length physical functioning dimension.

Physical functioning dimension: Eight weeks of exercise program or less

Study or Subgroup	High-intensity group Mean	SD	Total	Control group Mean	SD	Total	Weight	Std. Mean Difference IV, Random, 95% CI
Adams 2018	54.4	4.4	35	54.8	3.7	27	15.0%	-0.10 [-0.60, 0.41]
Cornie 2013	48.5	7.8	22	45.4	9.7	19	10.8%	0.35 [-0.27, 0.97]
Kampshoff 2015	88.1	9.5	91	84.1	13.1	91	29.1%	0.35 [0.06, 0.64]
Mijwel 2017	85.88	15.87	74	77.49	19.65	60	24.5%	0.47 [0.13, 0.82]
Persoon 2017	83.1	19.1	50	84.1	15.3	47	20.6%	-0.06 [-0.46, 0.34]
Total (95% CI)			272			244	100.0%	0.23 [0.00, 0.45]

Heterogeneity: Tau² = 0.02; Chi² = 6.23, df = 4 (P = 0.18); I² = 36%
Test for overall effect: Z = 1.98 (P = 0.05)

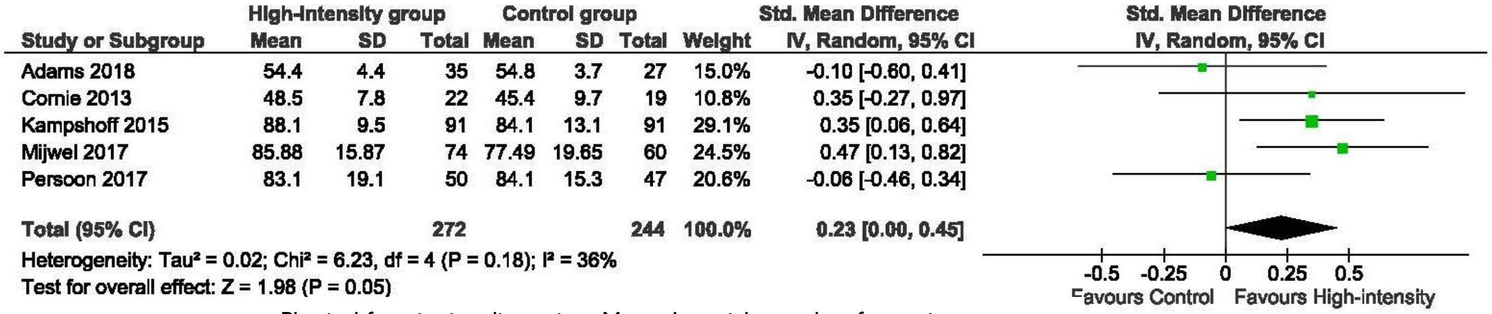

Physical functioning dimension: More than eight weeks of exercise program

Study or Subgroup	High-intensity group Mean	SD	Total	Control group Mean	SD	Total	Weight	Std. Mean Difference IV, Random, 95% CI
Adamsen 2009	89	12.4	135	86.4	14.5	134	85.5%	0.19 [-0.05, 0.43]
Hwang 2012	92.3	6.6	13	87.9	11.9	11	7.4%	0.45 [-0.36, 1.27]
Moller 2015 (a)	91.11	6.67	9	86.67	6.67	9	5.4%	0.63 [-0.32, 1.59]
Moller 2015 (b)	75	28.48	4	80	0.01	2	1.7%	-0.16 [-1.87, 1.54]
Total (95% CI)			161			156	100.0%	0.23 [0.01, 0.45]

Heterogeneity: Tau² = 0.00; Chi² = 1.28, df = 3 (P = 0.74); I² = 0%
Test for overall effect: Z = 2.03 (P = 0.04)

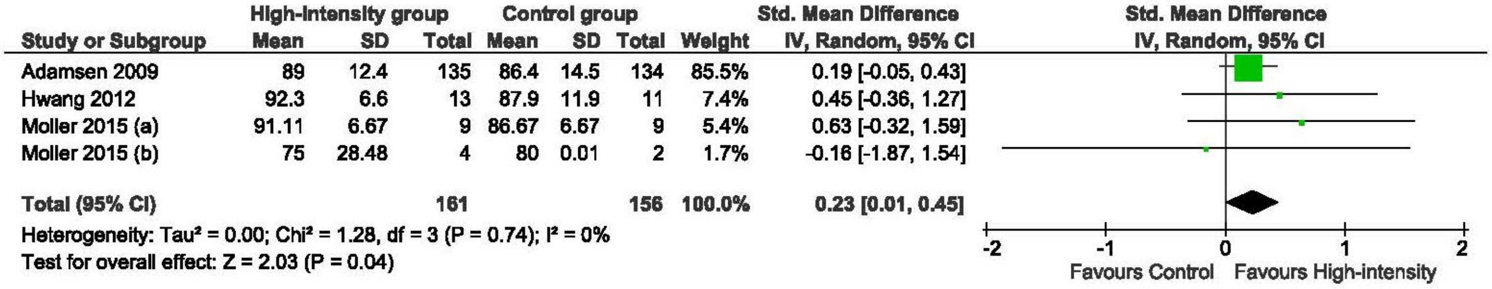

Supplementary Figure S22: Effects of the weekly exercise frequency in physical functioning dimension

Physical functioning dimension: 2 times per week

Study or Subgroup	High-intensity group Mean	SD	Total	Control group Mean	SD	Total	Weight	Std. Mean Difference IV, Random, 95% CI
Cormie 2013	48.5	7.8	22	45.4	9.7	19	11.3%	0.35 [-0.27, 0.97]
Kampshoff 2015	88.1	9.5	91	84.1	13.1	91	23.2%	0.35 [0.06, 0.64]
Mijwel 2017	85.88	15.87	74	77.49	19.65	60	20.7%	0.47 [0.13, 0.82]
Persoon 2017	83.1	19.1	50	84.1	15.3	47	18.4%	-0.06 [-0.46, 0.34]
Van Wart 2015	80.3	14.1	76	68.1	17.6	76	21.5%	0.76 [0.43, 1.09]
Van Wart 2017	75.2	16.2	6	77.1	15.3	8	4.9%	-0.11 [-1.17, 0.95]
**Total (95% CI)**			**319**			**301**	**100.0%**	**0.37 [0.11, 0.62]**

Heterogeneity: Tau² = 0.05; Chi² = 10.89, df = 5 (P = 0.05); I² = 54%
Test for overall effect: Z = 2.81 (P = 0.005)

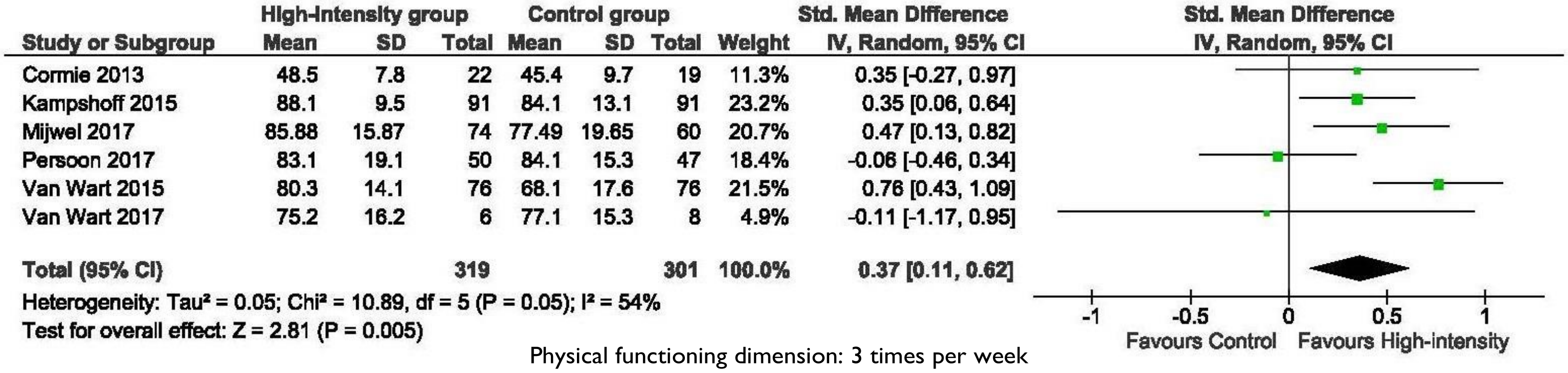

Physical functioning dimension: 3 times per week

Study or Subgroup	High-intensity group Mean	SD	Total	Control group Mean	SD	Total	Weight	Std. Mean Difference IV, Random, 95% CI
Adams 2018	54.4	4.4	35	54.8	3.7	27	16.3%	-0.10 [-0.60, 0.41]
Adamsen 2009	89	12.4	135	86.4	14.5	134	71.6%	0.19 [-0.05, 0.43]
Hwang 2012	92.3	6.6	13	87.9	11.9	11	6.2%	0.45 [-0.36, 1.27]
Moller 2015 (a)	91.11	6.67	9	86.67	6.67	9	4.5%	0.63 [-0.32, 1.59]
Moller 2015 (b)	75	28.48	4	80	0.01	2	1.4%	-0.16 [-1.87, 1.54]
**Total (95% CI)**			**196**			**183**	**100.0%**	**0.18 [-0.03, 0.38]**

Heterogeneity: Tau² = 0.00; Chi² = 2.62, df = 4 (P = 0.62); I² = 0%
Test for overall effect: Z = 1.71 (P = 0.09)

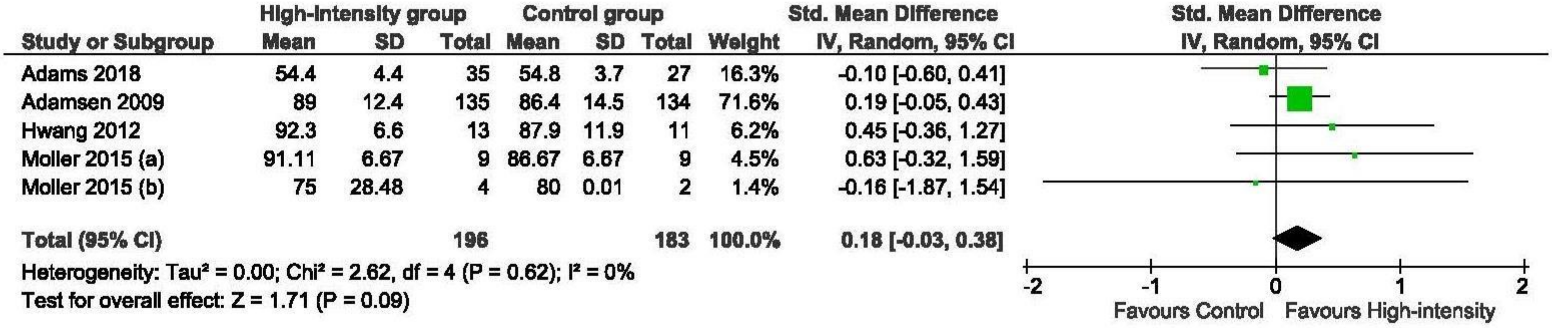

Supplementary Figure S23: Effects of training minutes per week in physical functioning dimension.

Physical fuctioning dimension:Less than 120 minutes of exercise per weeks

Study or Subgroup	High-intensity group Mean	SD	Total	Control group Mean	SD	Total	Weight	Std. Mean Difference IV, Random, 95% CI
Adamsen 2009	89	12.4	135	86.4	14.5	134	48.0%	0.19 [-0.05, 0.43]
Cormie 2013	48.5	7.8	22	45.4	9.7	19	7.3%	0.35 [-0.27, 0.97]
Mijwel 2017	85.88	15.87	74	77.49	19.65	60	23.2%	0.47 [0.13, 0.82]
Moller 2015 (a)	91.11	6.67	9	86.67	6.67	9	3.1%	0.63 [-0.32, 1.59]
Moller 2015 (b)	75	28.48	4	80	0.01	2	1.0%	-0.16 [-1.87, 1.54]
Persoon 2017	83.1	19.1	50	84.1	15.3	47	17.5%	-0.06 [-0.46, 0.34]
**Total (95% CI)**			**294**			**271**	**100.0%**	**0.24 [0.07, 0.40]**

Heterogeneity: Tau² = 0.00; Chi² = 5.01, df = 5 (P = 0.41); I² = 0%
Test for overall effect: Z = 2.76 (P = 0.006)

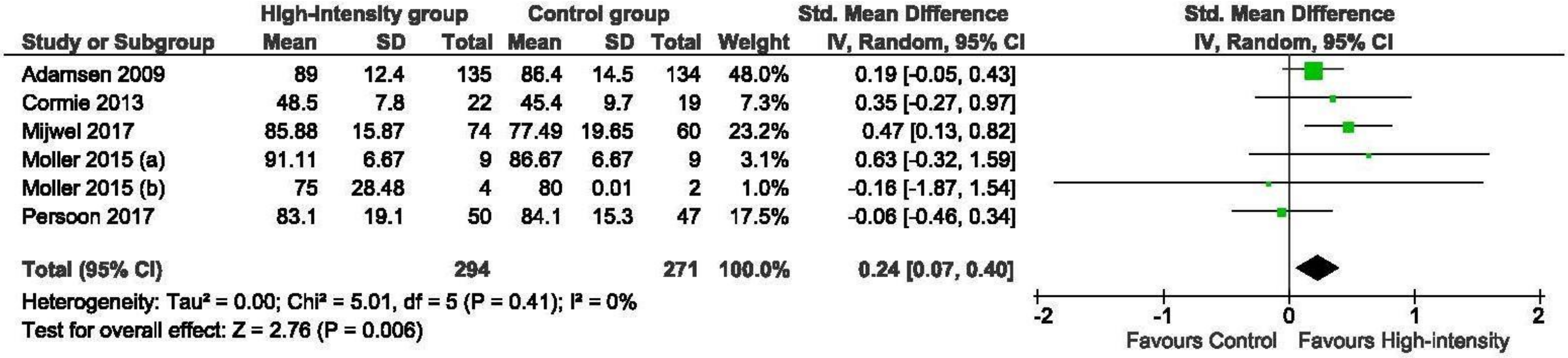

Physical functioning dimension: 120 minutes or more or exercise per weeks

Study or Subgroup	High-intensity group Mean	SD	Total	Control group Mean	SD	Total	Weight	Std. Mean Difference IV, Random, 95% CI
Adams 2018	54.4	4.4	35	54.8	3.7	27	29.5%	-0.10 [-0.60, 0.41]
Hwang 2012	92.3	6.6	13	87.9	11.9	11	20.4%	0.45 [-0.36, 1.27]
Van Wart 2015	80.3	14.1	76	68.1	17.6	76	34.9%	0.76 [0.43, 1.09]
Van Wart 2017	75.2	16.2	6	77.1	15.3	8	15.2%	-0.11 [-1.17, 0.95]
**Total (95% CI)**			**130**			**122**	**100.0%**	**0.31 [-0.21, 0.84]**

Heterogeneity: Tau² = 0.18; Chi² = 9.04, df = 3 (P = 0.03); I² = 67%
Test for overall effect: Z = 1.17 (P = 0.24)

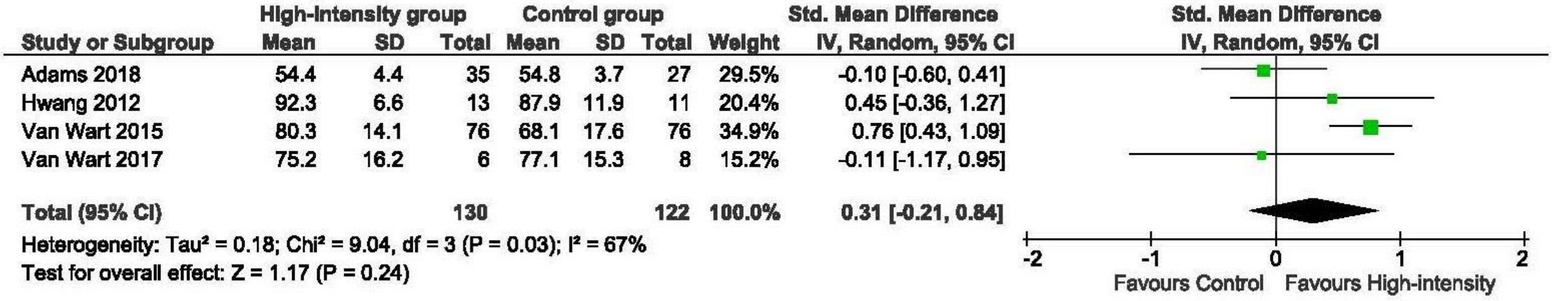

Supplementary Figure S24: Effects of the high-intensity training part duration in physical functioning dimension

Physical functioning dimension: 15 minutes or les of high-intensity training

	High-intensity group			Control group				Std. Mean Difference
Study or Subgroup	Mean	SD	Total	Mean	SD	Total	Weight	IV, Random, 95% CI
Adamsen 2009	89	12.4	135	86.4	14.5	134	63.9%	0.19 [-0.05, 0.43]
Mijwel 2017	85.88	15.87	74	77.49	19.65	60	30.8%	0.47 [0.13, 0.82]
Moller 2015 (a)	91.11	6.67	9	86.67	6.67	9	4.0%	0.63 [-0.32, 1.59]
Moller 2015 (b)	75	28.48	4	80	0.01	2	1.3%	-0.16 [-1.87, 1.54]
**Total (95% CI)**			**222**			**205**	**100.0%**	**0.29 [0.10, 0.48]**

Heterogeneity: Tau² = 0.00; Chi² = 2.48, df = 3 (P = 0.48); I² = 0%
Test for overall effect: Z = 2.99 (P = 0.003)

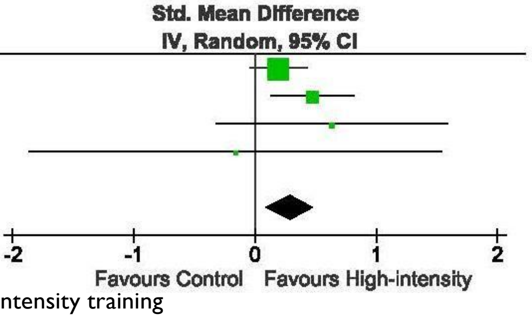

Physical functioning dimension: More than 15 minutes of high-intensity training

	High-intensity group			Control group				Std. Mean Difference
Study or Subgroup	Mean	SD	Total	Mean	SD	Total	Weight	IV, Random, 95% CI
Adams 2018	54.4	4.4	35	54.8	3.7	27	14.7%	-0.10 [-0.60, 0.41]
Cormie 2013	48.5	7.8	22	45.4	9.7	19	11.8%	0.35 [-0.27, 0.97]
Hwang 2012	92.3	6.6	13	87.9	11.9	11	8.3%	0.45 [-0.36, 1.27]
Kampshoff 2015	88.1	9.5	91	84.1	13.1	91	21.5%	0.35 [0.06, 0.64]
Persoon 2017	83.1	19.1	50	84.1	15.3	47	17.9%	-0.06 [-0.46, 0.34]
Van Wart 2015	80.3	14.1	76	68.1	17.6	76	20.2%	0.76 [0.43, 1.09]
Van Wart 2017	75.2	16.2	6	77.1	15.3	8	5.5%	-0.11 [-1.17, 0.95]
**Total (95% CI)**			**293**			**279**	**100.0%**	**0.28 [-0.00, 0.56]**

Heterogeneity: Tau² = 0.07; Chi² = 13.75, df = 6 (P = 0.03); I² = 56%
Test for overall effect: Z = 1.95 (P = 0.05)

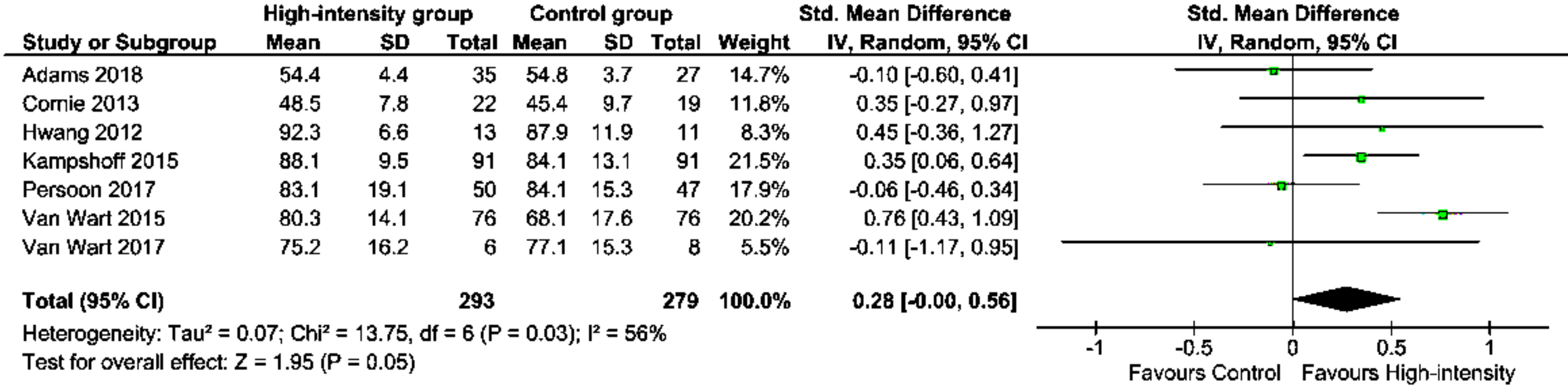

Supplementary Figure S25: Effects in the role functioning dimension of the comparisons between high-intensity training group and control group, and high-intensity training group and low- intensity exercise group.

Role functioning dimension: Comparison between high-intensity group and control group

	High-intensity group			Control group				Std. Mean Difference
Study or Subgroup	Mean	SD	Total	Mean	SD	Total	Weight	IV, Random, 95% CI
Adams 2018	53.4	7.2	35	53.7	6	27	7.4%	-0.04 [-0.55, 0.46]
Adamsen 2009	74.8	26.3	135	68.9	26.5	134	32.6%	0.22 [-0.02, 0.46]
Cormie 2013	49.8	7.8	22	48.1	10.5	19	5.0%	0.18 [-0.43, 0.80]
Hwang 2012	96.1	7.3	13	90.9	15.6	11	2.8%	0.42 [-0.39, 1.24]
Kampshoff 2015	82.5	21.4	91	82	21.4	91	22.2%	0.02 [-0.27, 0.31]
Mijwel 2017	71.59	27.25	74	54.84	33.03	60	15.6%	0.56 [0.21, 0.90]
Moller 2015 (a)	75.93	16.9	9	61.11	23.57	9	2.0%	0.69 [-0.27, 1.65]
Moller 2015 (b)	66.67	45.13	4	50	23.57	2	0.6%	0.33 [-1.40, 2.05]
Persoon 2017	81	23.8	50	73.4	31.8	47	11.7%	0.27 [-0.13, 0.67]
**Total (95% CI)**			**433**			**400**	**100.0%**	**0.23 [0.09, 0.37]**

Heterogeneity: Tau² = 0.00; Chi² = 7.65, df = 8 (P = 0.47); I² = 0%
Test for overall effect: Z = 3.29 (P = 0.001)

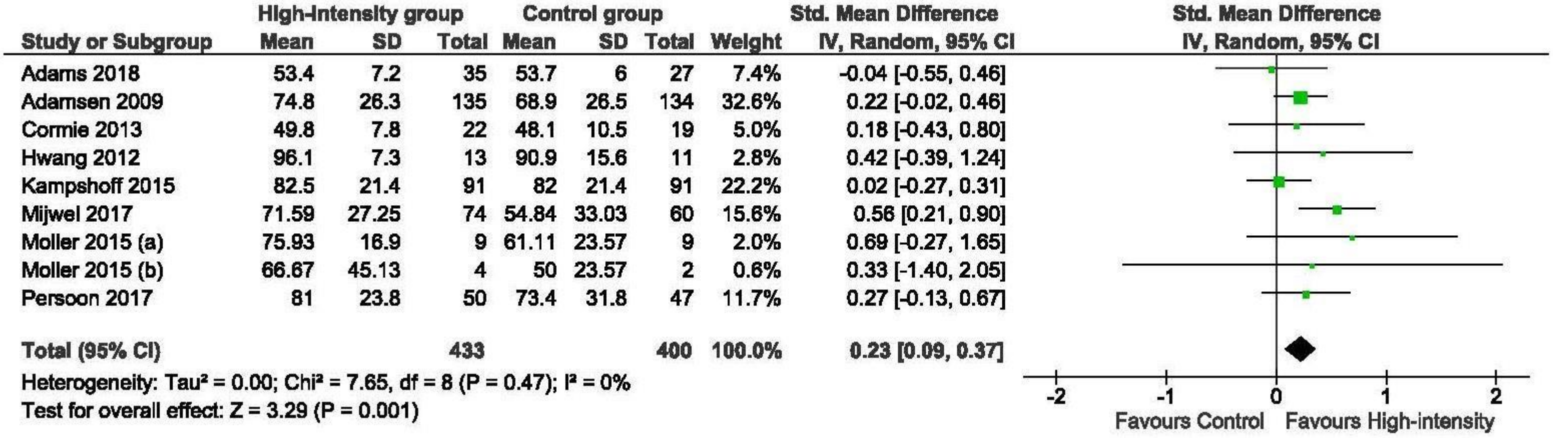

Role functioning dimension: Comparison between high-intensity group and low-moderate intensity group

	Experimental			Active group				Std. Mean Difference
Study or Subgroup	Mean	SD	Total	Mean	SD	Total	Weight	IV, Random, 95% CI
Cormie 2013	49.8	7.8	22	51.5	8	21	24.0%	-0.21 [-0.81, 0.39]
Kampshoff 2015	96.1	12.3	91	83.5	21.1	95	33.7%	0.72 [0.43, 1.02]
Moller 2015 (a)	75.93	16.9	9	72.22	33.33	9	15.8%	0.13 [-0.79, 1.06]
Moller 2015 (b)	66.67	45.13	4	72.22	25.46	3	8.0%	-0.12 [-1.62, 1.38]
Schmitt 2016	88.5	17.1	13	71.9	22	13	18.4%	0.82 [0.01, 1.62]
**Total (95% CI)**			**139**			**141**	**100.0%**	**0.35 [-0.12, 0.83]**

Heterogeneity: Tau² = 0.15; Chi² = 9.39, df = 4 (P = 0.05); I² = 57%
Test for overall effect: Z = 1.45 (P = 0.15)

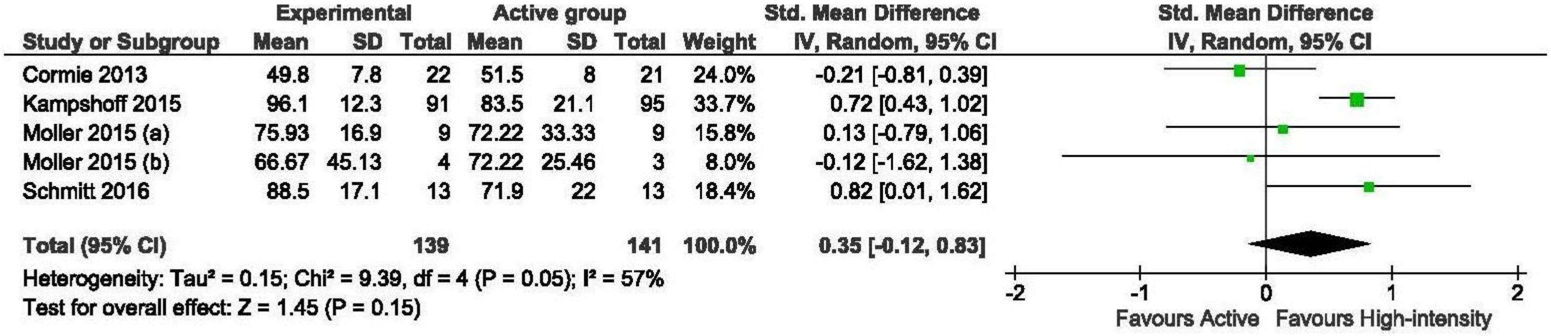

Supplementary Figure S26: Effects of high-intensity training in role functioning according to the treatment timing.

Role functioning dimension: During treatment

	High-Intensity group			Contro group				Std. Mean Difference
Study or Subgroup	Mean	SD	Total	Mean	SD	Total	Weight	IV, Random, 95% CI
Adams 2018	53.4	7.2	35	53.7	6	27	16.1%	-0.04 [-0.55, 0.46]
Cormie 2013	49.8	7.8	22	48.1	10.5	19	10.7%	0.18 [-0.43, 0.80]
Kampshoff 2015	82.5	21.4	91	82	21.4	91	48.0%	0.02 [-0.27, 0.31]
Persoon 2017	81	23.8	50	73.4	31.8	47	25.3%	0.27 [-0.13, 0.67]
**Total (95% CI)**			**198**			**184**	**100.0%**	**0.09 [-0.11, 0.29]**

Heterogeneity: Tau² = 0.00; Chi² = 1.34, df = 3 (P = 0.72); I² = 0%
Test for overall effect: Z = 0.89 (P = 0.37)

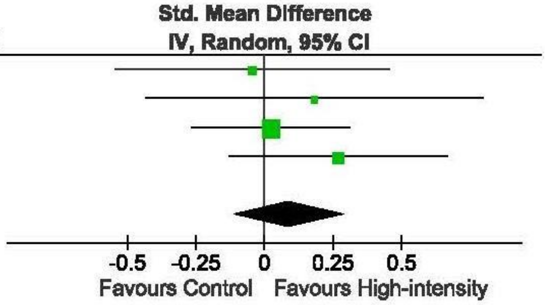

Role functioning dimension: After treatment

	High-Intensity group			Control group				Std. Mean Difference
Study or Subgroup	Mean	SD	Total	Mean	SD	Total	Weight	IV, Random, 95% CI
Adamsen 2009	74.8	26.3	135	68.9	26.5	134	60.8%	0.22 [-0.02, 0.46]
Hwang 2012	96.1	7.3	13	90.9	15.6	11	5.3%	0.42 [-0.39, 1.24]
Mijwel 2017	71.59	27.25	74	54.84	33.03	60	29.0%	0.56 [0.21, 0.90]
Moller 2015 (a)	75.93	16.9	9	61.11	23.57	9	3.8%	0.69 [-0.27, 1.65]
Moller 2015 (b)	66.67	45.13	4	50	23.57	2	1.2%	0.33 [-1.40, 2.05]
**Total (95% CI)**			**235**			**216**	**100.0%**	**0.35 [0.16, 0.54]**

Heterogeneity: Tau² = 0.00; Chi² = 2.94, df = 4 (P = 0.57); I² = 0%
Test for overall effect: Z = 3.66 (P = 0.0003)

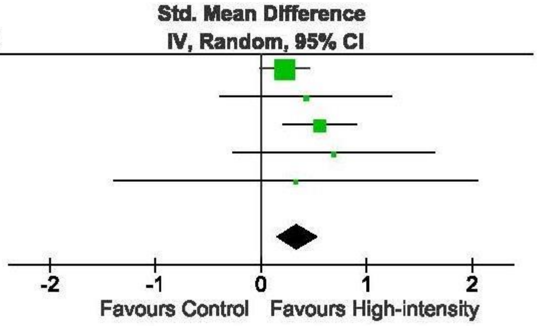

Supplementary Figure S27: Effects of the exercise programs length in role functioning dimension.

Role functioning dimension: Eight weeks of exercise program or less

	High-Intensity group			Control group				Std. Mean Difference
Study or Subgroup	Mean	SD	Total	Mean	SD	Total	Weight	IV, Random, 95% CI
Adamsen 2009	74.8	26.3	135	68.9	26.5	134	85.6%	0.22 [-0.02, 0.46]
Hwang 2012	96.1	7.3	13	90.9	15.6	11	7.4%	0.42 [-0.39, 1.24]
Moller 2015 (a)	75.93	16.9	9	61.11	23.57	9	5.4%	0.69 [-0.27, 1.65]
Moller 2015 (b)	66.67	45.13	4	50	23.57	2	1.7%	0.33 [-1.40, 2.05]
**Total (95% CI)**			**161**			**156**	**100.0%**	**0.26 [0.04, 0.49]**

Heterogeneity: Tau² = 0.00; Chi² = 1.02, df = 3 (P = 0.80); I² = 0%
Test for overall effect: Z = 2.34 (P = 0.02)

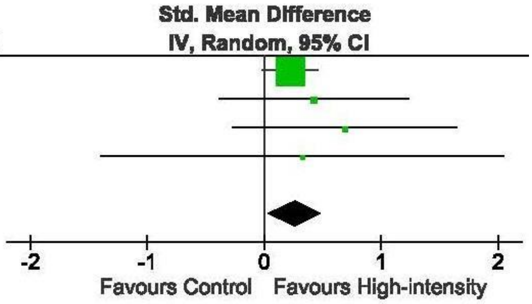

Role functioning dimension: More than eight weeks of exercise program

	High-Intensity group			Control group				Std. Mean Difference
Study or Subgroup	Mean	SD	Total	Mean	SD	Total	Weight	IV, Random, 95% CI
Adams 2018	53.4	7.2	35	53.7	6	27	15.2%	-0.04 [-0.55, 0.46]
Cormie 2013	49.8	7.8	22	48.1	10.5	19	11.2%	0.18 [-0.43, 0.80]
Kampshoff 2015	82.5	21.4	91	82	21.4	91	29.0%	0.02 [-0.27, 0.31]
Mijwel 2017	71.59	27.25	74	54.84	33.03	60	24.2%	0.56 [0.21, 0.90]
Persoon 2017	81	23.8	50	73.4	31.8	47	20.5%	0.27 [-0.13, 0.67]
**Total (95% CI)**			**272**			**244**	**100.0%**	**0.21 [-0.02, 0.44]**

Heterogeneity: Tau² = 0.03; Chi² = 6.47, df = 4 (P = 0.17); I² = 38%
Test for overall effect: Z = 1.78 (P = 0.07)

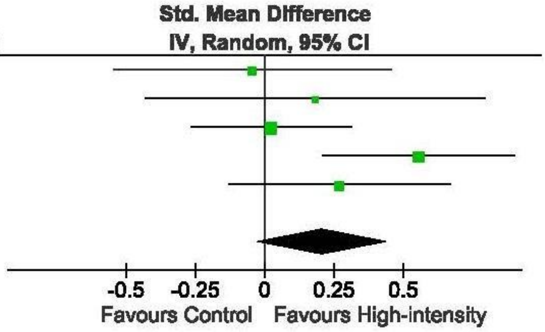

Supplementary Figure S28: Effects of the weekly exercise frequency in role functioning dimension.

Role functioning dimension: 2 times per week

	High-intensity group			Control group				Std. Mean Difference
Study or Subgroup	Mean	SD	Total	Mean	SD	Total	Weight	IV, Random, 95% CI
Cormie 2013	49.8	7.8	22	48.1	10.5	19	13.6%	0.18 [-0.43, 0.80]
Kampshoff 2015	82.5	21.4	91	82	21.4	91	33.5%	0.02 [-0.27, 0.31]
Mijwel 2017	71.59	27.25	74	54.84	33.03	60	28.4%	0.56 [0.21, 0.90]
Persoon 2017	81	23.8	50	73.4	31.8	47	24.4%	0.27 [-0.13, 0.67]
**Total (95% CI)**			**237**			**217**	**100.0%**	**0.26 [-0.00, 0.52]**

Heterogeneity: Tau² = 0.03; Chi² = 5.37, df = 3 (P = 0.15); I² = 44%
Test for overall effect: Z = 1.93 (P = 0.05)

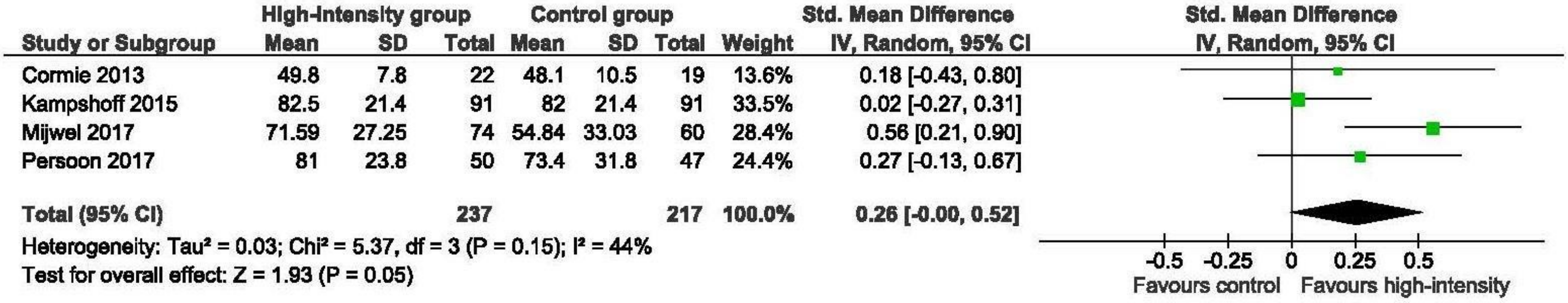

Role functioning dimension: 3 times per week

	High-intensity group			Control group				Std. Mean Difference
Study or Subgroup	Mean	SD	Total	Mean	SD	Total	Weight	IV, Random, 95% CI
Adams 2018	53.4	7.2	35	53.7	6	27	16.3%	-0.04 [-0.55, 0.46]
Adamsen 2009	74.8	26.3	135	68.9	26.5	134	71.6%	0.22 [-0.02, 0.46]
Hwang 2012	96.1	7.3	13	90.9	15.6	11	6.2%	0.42 [-0.39, 1.24]
Moller 2015 (a)	75.93	16.9	9	61.11	23.57	9	4.5%	0.69 [-0.27, 1.65]
Moller 2015 (b)	66.67	45.13	4	50	23.57	2	1.4%	0.33 [-1.40, 2.05]
**Total (95% CI)**			**196**			**183**	**100.0%**	**0.21 [0.01, 0.42]**

Heterogeneity: Tau² = 0.00; Chi² = 2.24, df = 4 (P = 0.69); I² = 0%
Test for overall effect: Z = 2.07 (P = 0.04)

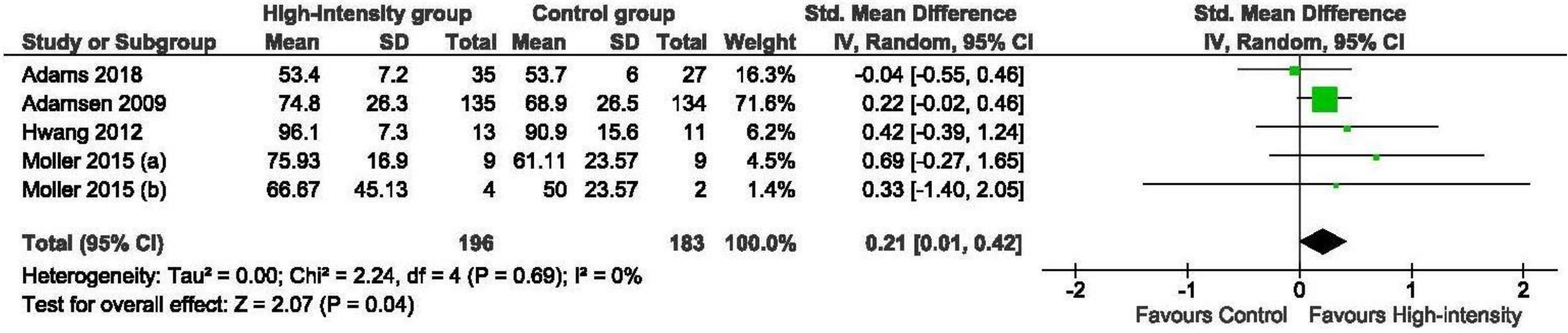

Supplementary Figure S29: Effects of training minutes per week in role functioning dimension.

Role functioning dimension: 15 minutes or less of high-intensity training

Study or Subgroup	Mean	SD	Total	Mean	SD	Total	Weight	IV, Random, 95% CI
Adamsen 2009	74.8	26.3	135	68.9	26.5	134	64.1%	0.22 [-0.02, 0.46]
Mijwel 2017	71.59	27.25	74	54.84	33.03	60	30.6%	0.56 [0.21, 0.90]
Moller 2015 (a)	75.93	16.9	9	61.11	23.57	9	4.0%	0.69 [-0.27, 1.65]
Moller 2015 (b)	66.67	45.13	4	50	23.57	2	1.2%	0.33 [-1.40, 2.05]
**Total (95% CI)**			**222**			**205**	**100.0%**	**0.34 [0.15, 0.54]**

Heterogeneity: Tau² = 0.00; Chi² = 2.91, df = 3 (P = 0.41); I² = 0%
Test for overall effect: Z = 3.52 (P = 0.0004)

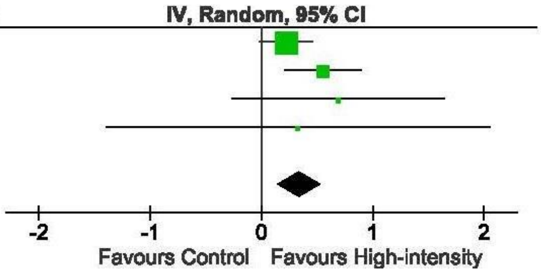

Role functioning dimension: More than 15 minutes of high-intensity trai

	High-intensity group			Control group				Std. Mean Difference
Study or Subgroup	Mean	SD	Total	Mean	SD	Total	Weight	IV, Random, 95% CI
Adams 2018	53.4	7.2	35	53.7	6	27	15.1%	-0.04 [-0.55, 0.46]
Cormie 2013	49.8	7.8	22	48.1	10.5	19	10.1%	0.18 [-0.43, 0.80]
Hwang 2012	96.1	7.3	13	90.9	15.6	11	5.8%	0.42 [-0.39, 1.24]
Kampshoff 2015	82.5	21.4	91	82	21.4	91	45.2%	0.02 [-0.27, 0.31]
Persoon 2017	81	23.8	50	73.4	31.8	47	23.8%	0.27 [-0.13, 0.67]
**Total (95% CI)**			**211**			**195**	**100.0%**	**0.11 [-0.08, 0.31]**

Heterogeneity: Tau² = 0.00; Chi² = 1.94, df = 4 (P = 0.75); I² = 0%
Test for overall effect: Z = 1.11 (P = 0.27)

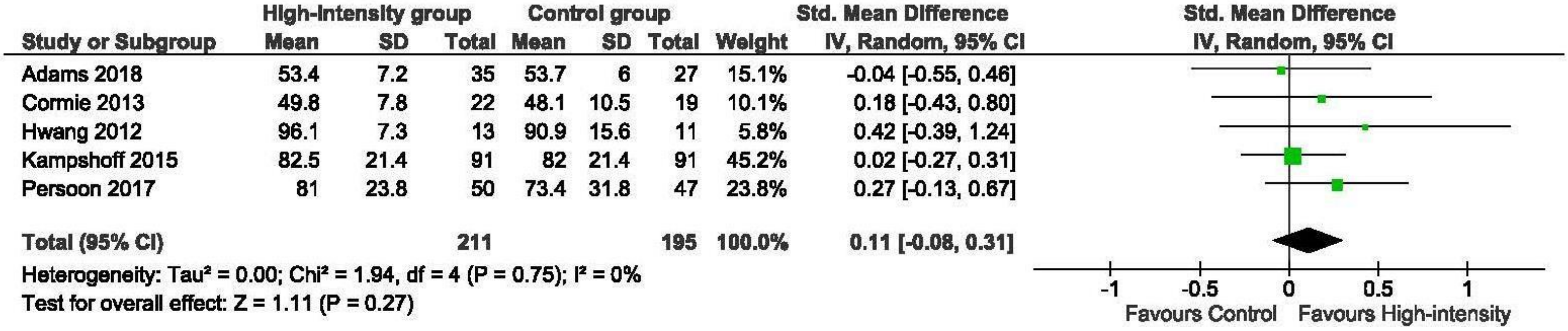

Supplementary Figure S30: Effects of high-intensity exercise in emotional functioning according to the treatment timing.

Emotional functioning dimension: Comparison between high-intensity group and control group.

Study or Subgroup	High-intensity group Mean	SD	Total	Control group Mean	SD	Total	Weight	Std. Mean Difference IV, Random, 95% CI
Adams 2018	53.4	5.2	35	51	8.3	27	5.3%	0.35 [-0.15, 0.86]
Adamsen 2009	81.3	17.2	135	80.6	17.8	134	23.8%	0.04 [-0.20, 0.28]
Andersen 2013	19.25	3.76	106	18.91	3.73	107	18.8%	0.09 [-0.18, 0.36]
Cormie 2013	47.8	11.2	22	48.4	12.6	19	3.6%	-0.05 [-0.66, 0.56]
Egegaard 2019	18.9	3.8	8	20.2	3.1	5	1.1%	-0.34 [-1.47, 0.79]
Hwang 2012	91.7	12.3	13	85.6	13	11	2.0%	0.47 [-0.35, 1.28]
Kampshoff 2015	88.3	15.3	91	83.3	17.5	91	15.9%	0.30 [0.01, 0.60]
Martin 2015 (a)	19.6	2.6	12	20.6	3.2	23	2.8%	-0.32 [-1.03, 0.38]
Martin 2015 (c)	20	3.5	26	20.3	2.8	25	4.5%	-0.09 [-0.64, 0.46]
Mijwel 2017	72.24	22.23	74	70.1	25.6	60	11.7%	0.09 [-0.25, 0.43]
Moller 2015 (a)	87.04	16.72	9	74.07	20.17	9	1.5%	0.67 [-0.29, 1.62]
Moller 2015 (b)	66.67	24.53	4	54.17	5.89	2	0.4%	0.47 [-1.29, 2.22]
Persoon 2017	86.2	16.3	50	86	14.7	47	8.6%	0.01 [-0.39, 0.41]
**Total (95% CI)**			**585**			**560**	**100.0%**	**0.11 [-0.01, 0.22]**

Heterogeneity: Tau² = 0.00; Chi² = 8.21, df = 12 (P = 0.77); I² = 0%
Test for overall effect: Z = 1.81 (P = 0.07)

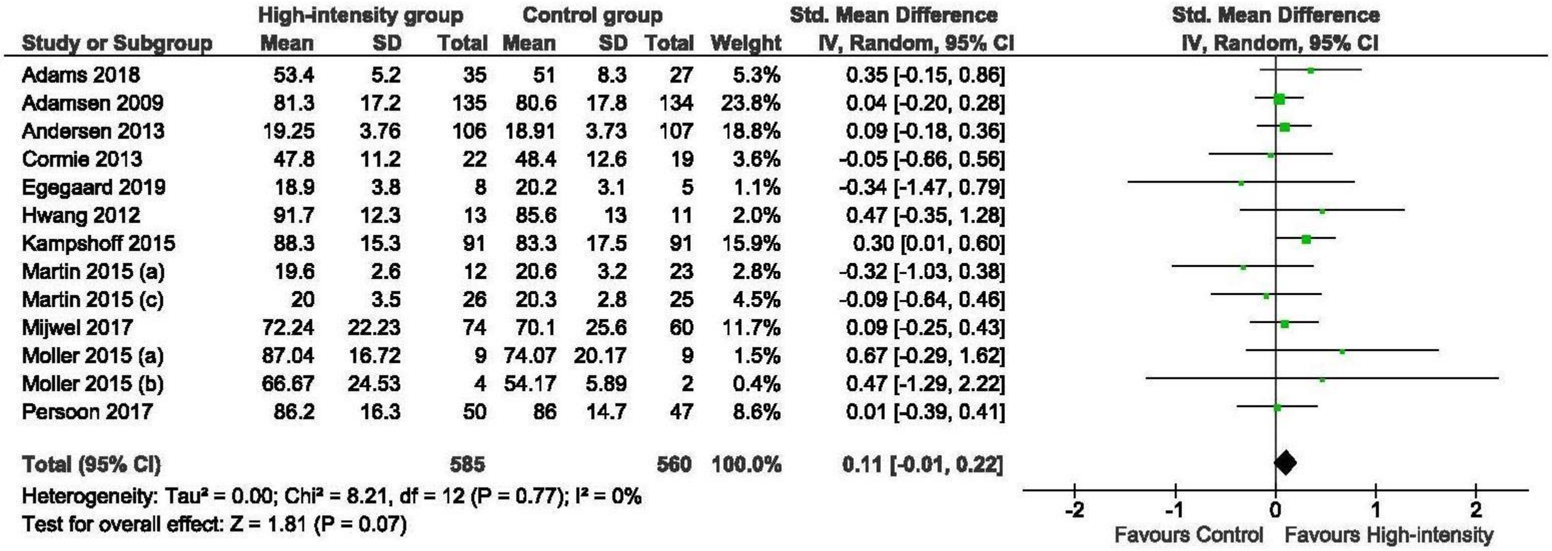

Emotional functioning dimension: Comparison between high-intensity group and low-moderate intensity group

Study or Subgroup	High-intensity group Mean	SD	Total	Active group Mean	SD	Total	Weight	Std. Mean Difference IV, Random, 95% CI
Cormie 2013	47.8	11.2	22	54.4	7.8	21	13.8%	-0.67 [-1.28, -0.05]
Kampshoff 2015	88.3	15.3	91	84	17.3	95	33.9%	0.26 [-0.03, 0.55]
Martin 2015 (a)	19.6	2.6	12	19.6	4	18	10.6%	0.00 [-0.73, 0.73]
Martin 2015 (c)	20	3.5	26	20.3	3.7	24	16.1%	-0.08 [-0.64, 0.47]
Moller 2015 (a)	87.04	16.72	9	82.41	19.3	9	7.0%	0.24 [-0.68, 1.17]
Moller 2015 (b)	66.67	24.53	4	77.78	4.81	3	2.8%	-0.49 [-2.03, 1.06]
Schmitt 2016	71.9	18.7	13	71.2	25.9	13	9.7%	0.03 [-0.74, 0.80]
Toohey 2016	19.38	2.62	8	17.75	3.01	8	6.1%	0.55 [-0.46, 1.55]
**Total (95% CI)**			**185**			**191**	**100.0%**	**0.02 [-0.24, 0.29]**

Heterogeneity: Tau² = 0.03; Chi² = 9.05, df = 7 (P = 0.25); I² = 23%
Test for overall effect: Z = 0.17 (P = 0.86)

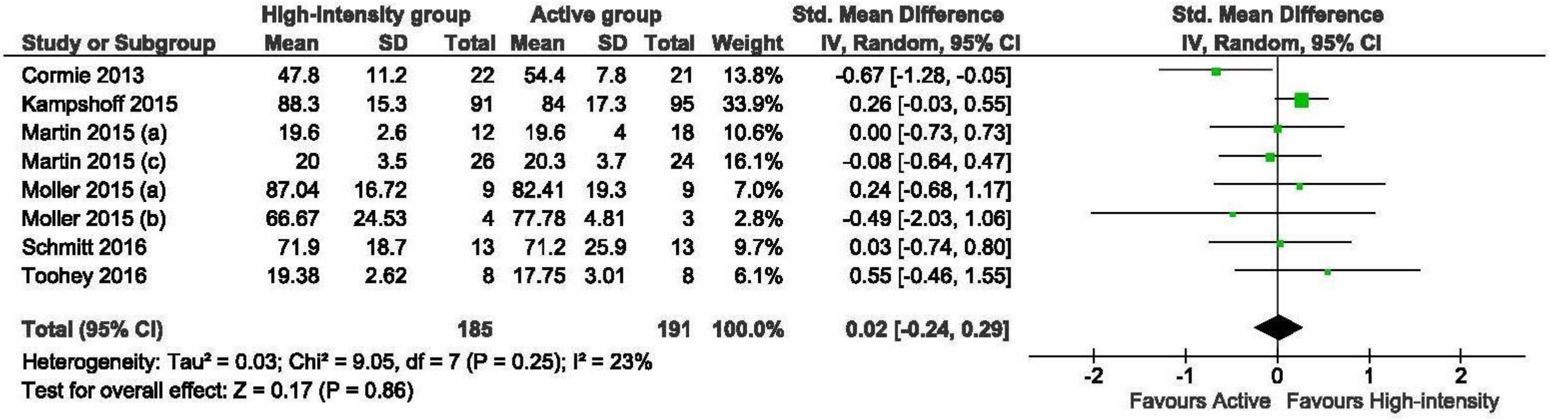

Supplementary Figure S31: Effects of high-intensity training in global health according to the treatment timing.

Emotional functioning dimension: During treatment

Study or Subgroup	High-intensity group Mean	SD	Total	Control group Mean	SD	Total	Weight	Std. Mean Difference IV, Random, 95% CI
Adamsen 2009	81.3	17.2	135	80.6	17.8	134	40.1%	0.04 [-0.20, 0.28]
Andersen 2013	19.25	3.76	106	18.91	3.73	107	31.7%	0.09 [-0.18, 0.36]
Egegaard 2019	18.9	3.8	8	20.2	3.1	5	1.8%	-0.34 [-1.47, 0.79]
Hwang 2012	91.7	12.3	13	85.6	13	11	3.4%	0.47 [-0.35, 1.28]
Mijwel 2017	72.24	22.23	74	70.1	25.6	60	19.7%	0.09 [-0.25, 0.43]
Moller 2015 (a)	87.04	16.72	9	74.07	20.17	9	2.5%	0.67 [-0.29, 1.62]
Moller 2015 (b)	66.67	24.53	4	54.17	5.89	2	0.7%	0.47 [-1.29, 2.22]
**Total (95% CI)**			**349**			**328**	**100.0%**	**0.09 [-0.06, 0.24]**

Heterogeneity: Tau² = 0.00; Chi² = 3.12, df = 6 (P = 0.79); I² = 0%
Test for overall effect: Z = 1.20 (P = 0.23)

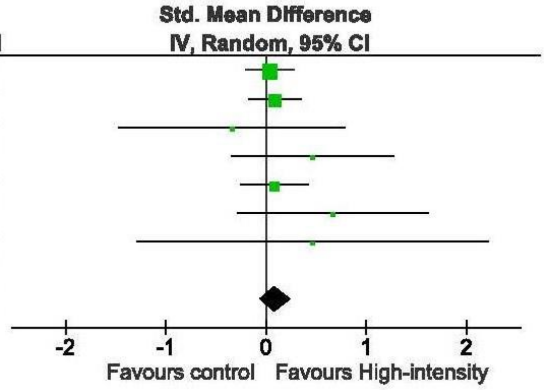

Emotional functioning dimension: After treatment

Study or Subgroup	High-intensity group Mean	SD	Total	Control group Mean	SD	Total	Weight	Std. Mean Difference IV, Random, 95% CI
Adams 2018	53.4	5.2	35	51	8.3	27	13.1%	0.35 [-0.15, 0.86]
Cormie 2013	47.8	11.2	22	48.4	12.6	19	8.9%	-0.05 [-0.66, 0.56]
Kampshoff 2015	88.3	15.3	91	83.3	17.5	91	39.1%	0.30 [0.01, 0.60]
Martin 2015 (a)	19.6	2.6	12	20.6	3.2	23	6.8%	-0.32 [-1.03, 0.38]
Martin 2015 (c)	20	3.5	26	20.3	2.8	25	11.1%	-0.09 [-0.64, 0.46]
Persoon 2017	86.2	16.3	50	86	14.7	47	21.1%	0.01 [-0.39, 0.41]
**Total (95% CI)**			**236**			**232**	**100.0%**	**0.13 [-0.05, 0.31]**

Heterogeneity: Tau² = 0.00; Chi² = 4.99, df = 5 (P = 0.42); I² = 0%
Test for overall effect: Z = 1.40 (P = 0.16)

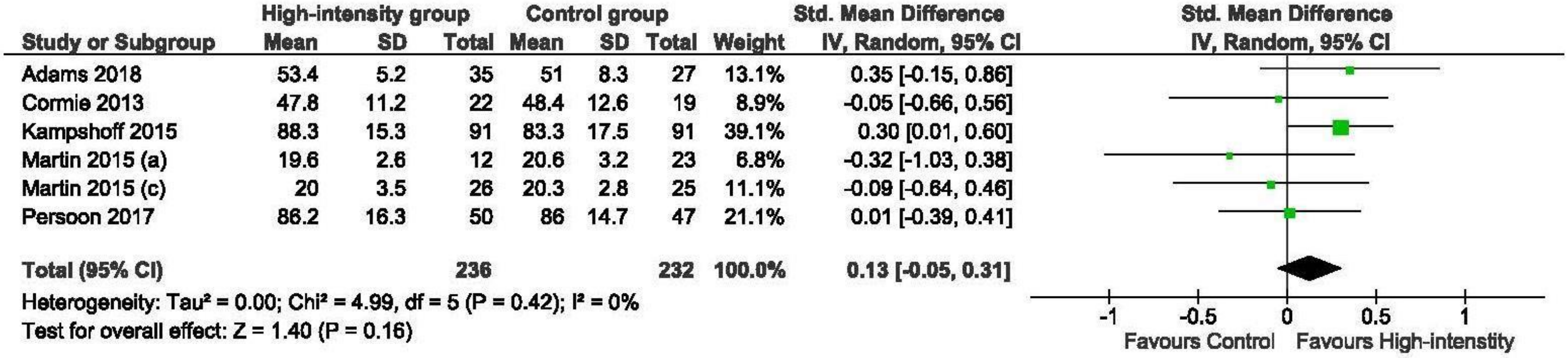

Supplementary Figure S32: Effects of the type pf exercise in global health dimension.

Emotional functioning dimension: High-intensity cardiovascular training

	High-intensity group			Control group				Std. Mean Difference
Study or Subgroup	Mean	SD	Total	Mean	SD	Total	Weight	IV, Random, 95% CI
Adams 2018	53.4	5.2	35	51	8.3	27	27.1%	0.35 [-0.15, 0.86]
Egegaard 2019	18.9	3.8	8	20.2	3.1	5	5.4%	-0.34 [-1.47, 0.79]
Hwang 2012	91.7	12.3	13	85.6	13	11	10.4%	0.47 [-0.35, 1.28]
Mijwel 2017	78.91	15.84	70	70.1	25.6	60	57.1%	0.42 [0.07, 0.77]
**Total (95% CI)**			**126**			**103**	**100.0%**	**0.36 [0.10, 0.63]**

Heterogeneity: Tau² = 0.00; Chi² = 1.65, df = 3 (P = 0.65); I² = 0%
Test for overall effect: Z = 2.71 (P = 0.007)

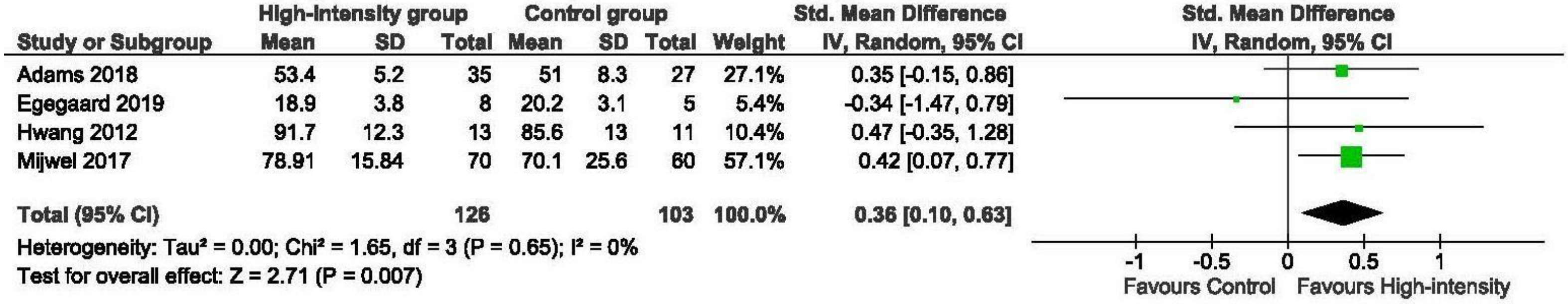

Emotional functioning dimension: High-intensity cardiovascular training + Resistance training

	High-intensity group			Control group				Std. Mean Difference
Study or Subgroup	Mean	SD	Total	Mean	SD	Total	Weight	IV, Random, 95% CI
Adamsen 2009	81.3	17.2	135	80.6	17.8	134	26.0%	0.04 [-0.20, 0.28]
Andersen 2013	19.25	3.76	106	18.91	3.73	107	20.5%	0.09 [-0.18, 0.36]
Cormie 2013	47.8	11.2	22	48.4	12.6	19	3.9%	-0.05 [-0.66, 0.56]
Kampshoff 2015	88.3	15.3	91	83.3	17.5	91	17.4%	0.30 [0.01, 0.60]
Martin 2015 (a)	19.6	2.6	12	20.6	3.2	23	3.0%	-0.32 [-1.03, 0.38]
Martin 2015 (b)	20	3.5	26	20.3	2.8	25	4.9%	-0.09 [-0.64, 0.46]
Mijwel 2017	72.24	22.23	74	70.1	25.6	60	12.8%	0.09 [-0.25, 0.43]
Moller 2015 (a)	87.04	16.72	9	74.07	20.17	9	1.6%	0.67 [-0.29, 1.62]
Moller 2015 (b)	66.67	24.53	4	54.17	5.89	2	0.5%	0.47 [-1.29, 2.22]
Persoon 2017	86.2	16.3	50	86	14.7	47	9.4%	0.01 [-0.39, 0.41]
**Total (95% CI)**			**529**			**517**	**100.0%**	**0.09 [-0.03, 0.21]**

Heterogeneity: Tau² = 0.00; Chi² = 5.89, df = 9 (P = 0.75); I² = 0%
Test for overall effect: Z = 1.46 (P = 0.14)

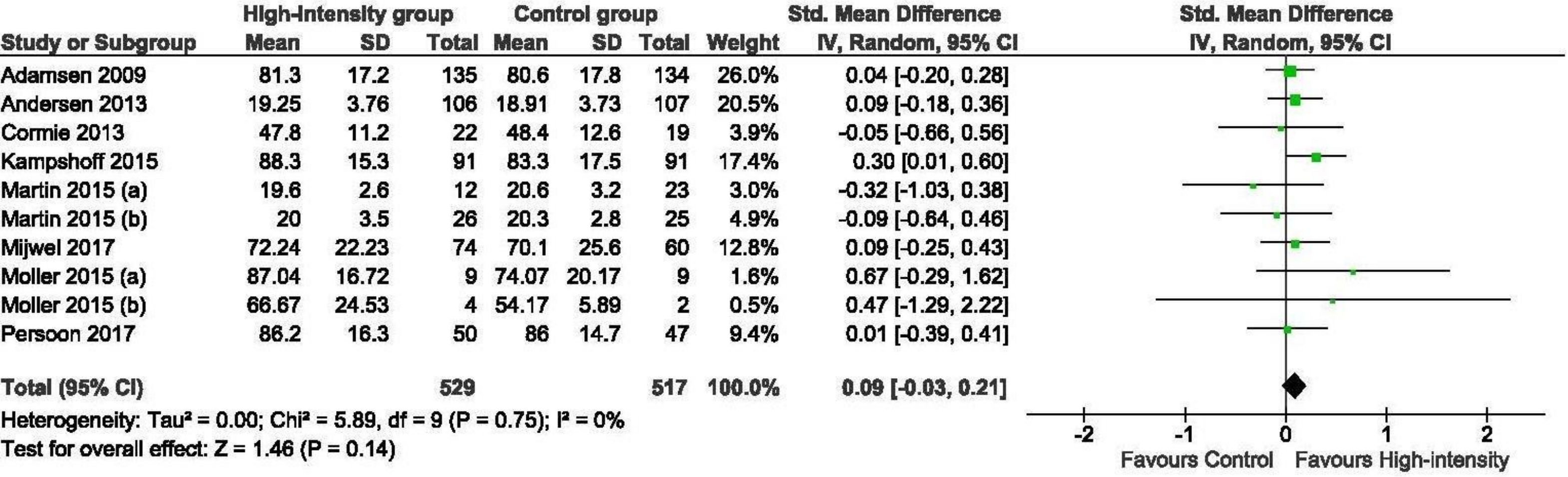

Supplementary Figure S33 Effects of the exercise programs length in emotional functioning dimension.

Emotional functioning dimension: Eight weeks of exercise program or less

	High-intensity group			Control group				Std. Mean Difference
Study or Subgroup	Mean	SD	Total	Mean	SD	Total	Weight	IV, Random, 95% CI
Adamsen 2009	81.3	17.2	135	80.6	17.8	134	43.3%	0.04 [-0.20, 0.28]
Andersen 2013	19.25	3.76	106	18.91	3.73	107	34.3%	0.09 [-0.18, 0.36]
Egegaard 2019	18.9	3.8	8	20.2	3.1	5	1.9%	-0.34 [-1.47, 0.79]
Hwang 2012	91.7	12.3	13	85.6	13	11	3.7%	0.47 [-0.35, 1.28]
Martin 2015 (a)	19.6	2.6	12	20.6	3.2	23	5.0%	-0.32 [-1.03, 0.38]
Martin 2015 (c)	20	3.5	26	20.3	2.8	25	8.2%	-0.09 [-0.64, 0.46]
Moller 2015 (a)	87.04	16.72	9	74.07	20.17	9	2.7%	0.67 [-0.29, 1.62]
Moller 2015 (b)	66.67	24.53	4	54.17	5.89	2	0.8%	0.47 [-1.29, 2.22]
**Total (95% CI)**			**313**			**316**	**100.0%**	**0.06 [-0.10, 0.21]**

Heterogeneity: Tau² = 0.00; Chi² = 4.71, df = 7 (P = 0.70); I² = 0%
Test for overall effect: Z = 0.71 (P = 0.48)

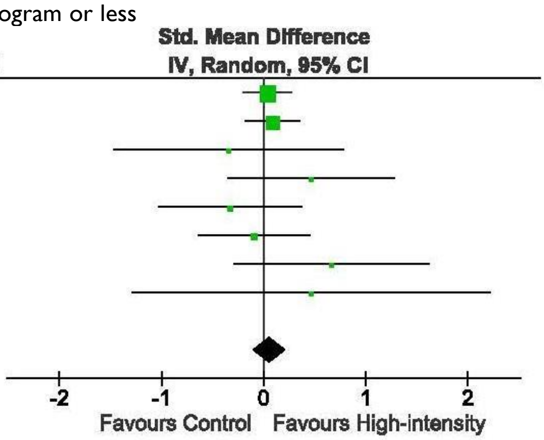

Emotional functioning dimension: More than eight weeks of exercise program

	High-intensity group			Control group				Std. Mean Difference
Study or Subgroup	Mean	SD	Total	Mean	SD	Total	Weight	IV, Random, 95% CI
Adams 2018	53.4	5.2	35	51	8.3	27	11.8%	0.35 [-0.15, 0.86]
Cormie 2013	47.8	11.2	22	48.4	12.6	19	8.0%	-0.05 [-0.66, 0.56]
Kampshoff 2015	88.3	15.3	91	83.3	17.5	91	35.3%	0.30 [0.01, 0.60]
Mijwel 2017	72.24	22.23	74	70.1	25.6	60	26.0%	0.09 [-0.25, 0.43]
Persoon 2017	86.2	16.3	50	86	14.7	47	19.0%	0.01 [-0.39, 0.41]
**Total (95% CI)**			**272**			**244**	**100.0%**	**0.17 [-0.00, 0.34]**

Heterogeneity: Tau² = 0.00; Chi² = 2.60, df = 4 (P = 0.63); I² = 0%
Test for overall effect: Z = 1.92 (P = 0.05)

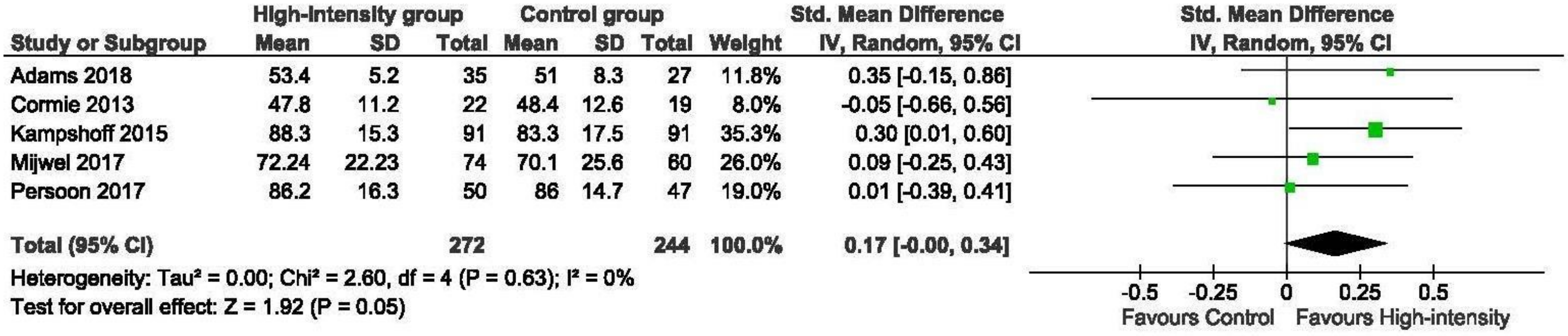

Supplementary Figure S34: Effects of the weekly exercise frequency in emotional functioning dimension.

Emotional functioning dimension: 2 times per week

Study or Subgroup	High-intensity group Mean	SD	Total	Control group Mean	SD	Total	Weight	Std. Mean Difference IV, Random, 95% CI
Cormie 2013	47.8	11.2	22	48.4	12.6	19	9.1%	-0.05 [-0.66, 0.56]
Kampshoff 2015	88.3	15.3	91	83.3	17.5	91	40.0%	0.30 [0.01, 0.60]
Mijwel 2017	72.24	22.23	74	70.1	25.6	60	29.4%	0.09 [-0.25, 0.43]
Persoon 2017	86.2	16.3	50	86	14.7	47	21.5%	0.01 [-0.39, 0.41]
**Total (95% CI)**			**237**			**217**	**100.0%**	**0.15 [-0.04, 0.33]**

Heterogeneity: Tau² = 0.00; Chi² = 2.03, df = 3 (P = 0.57); I² = 0%
Test for overall effect: Z = 1.55 (P = 0.12)

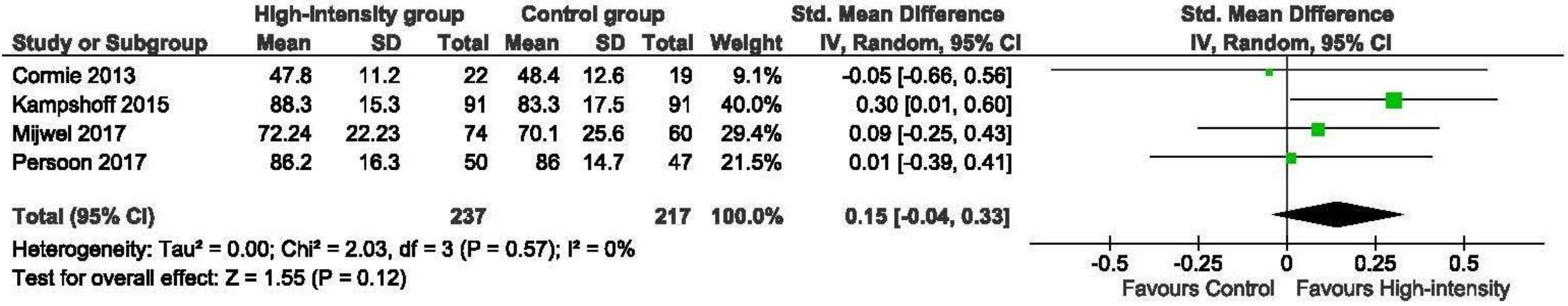

Emotional functioning dimension: 3 times per week

Study or Subgroup	High-intensity group Mean	SD	Total	Control group Mean	SD	Total	Weight	Std. Mean Difference IV, Random, 95% CI
Adams 2018	53.4	5.2	35	51	8.3	27	9.0%	0.35 [-0.15, 0.86]
Adamsen 2009	81.3	17.2	135	80.6	17.8	134	40.2%	0.04 [-0.20, 0.28]
Andersen 2013	19.25	3.76	106	18.91	3.73	107	31.8%	0.09 [-0.18, 0.36]
Hwang 2012	91.7	12.3	13	85.6	13	11	3.5%	0.47 [-0.35, 1.28]
Martin 2015 (a)	19.6	2.6	12	20.6	3.2	23	4.7%	-0.32 [-1.03, 0.38]
Martin 2015 (c)	20	3.5	26	20.3	2.8	25	7.6%	-0.09 [-0.64, 0.46]
Moller 2015 (a)	87.04	16.72	9	74.07	20.17	9	2.5%	0.67 [-0.29, 1.62]
Moller 2015 (b)	66.67	24.53	4	54.17	5.89	2	0.7%	0.47 [-1.29, 2.22]
**Total (95% CI)**			**340**			**338**	**100.0%**	**0.09 [-0.06, 0.24]**

Heterogeneity: Tau² = 0.00; Chi² = 5.36, df = 7 (P = 0.62); I² = 0%
Test for overall effect: Z = 1.17 (P = 0.24)

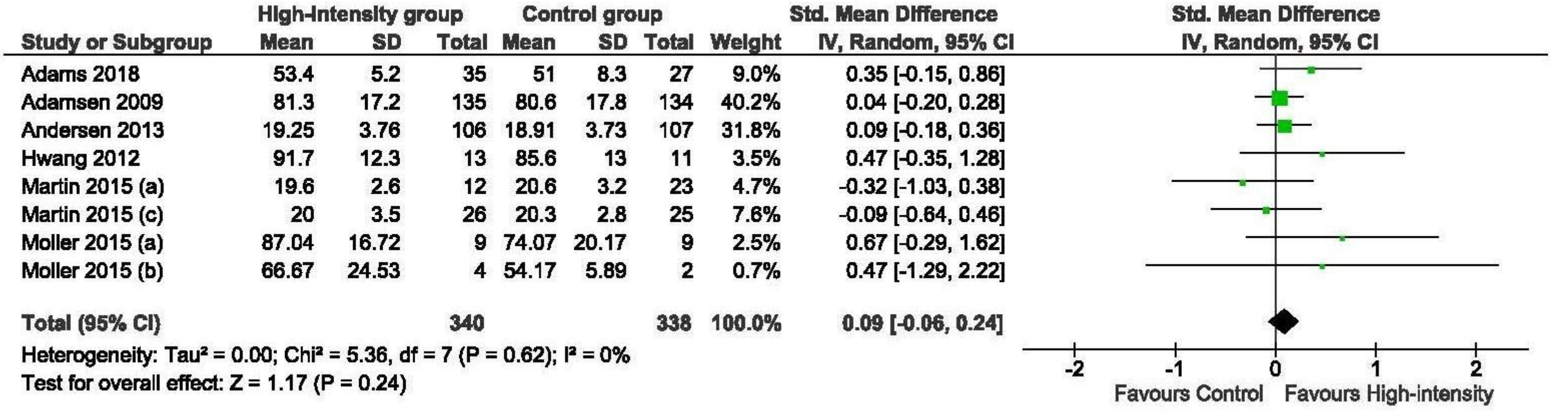

Supplementary Figure S35: Effects of training minutes per week in emotional functioning dimension.

Emotional functioning dimension: Less than 120 minutes of exercise per week

Study or Subgroup	High-intensity group Mean	SD	Total	Control group Mean	SD	Total	Weight	Std. Mean Difference IV, Random, 95% CI
Adams 2018	53.4	5.2	35	51	8.3	27	63.0%	0.35 [-0.15, 0.86]
Egegaard 2019	18.9	3.8	8	20.2	3.1	5	12.7%	-0.34 [-1.47, 0.79]
Hwang 2012	91.7	12.3	13	85.6	13	11	24.3%	0.47 [-0.35, 1.28]
**Total (95% CI)**			**56**			**43**	**100.0%**	**0.29 [-0.11, 0.69]**

Heterogeneity: Tau² = 0.00; Chi² = 1.43, df = 2 (P = 0.49); I² = 0%
Test for overall effect: Z = 1.43 (P = 0.15)

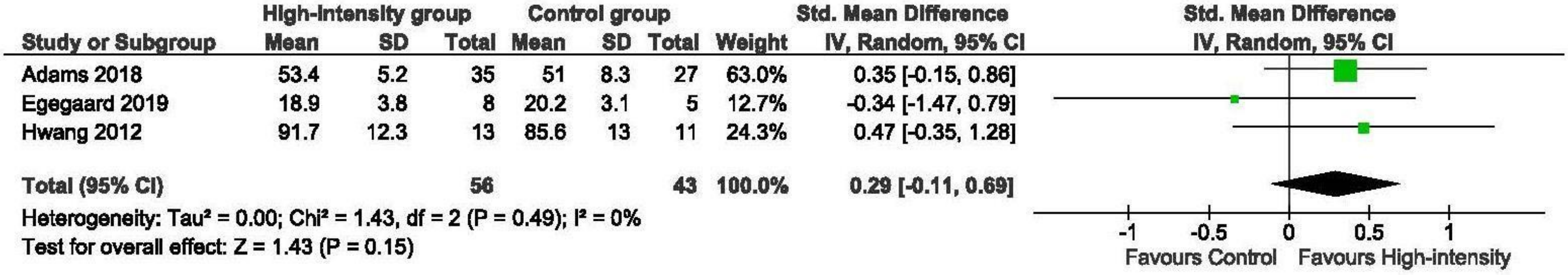

Emotional functioning dimension:120 minutes or more or exercise per week

Study or Subgroup	High-intensity group Mean	SD	Total	Control group Mean	SD	Total	Weight	Std. Mean Difference IV, Random, 95% CI
Adamsen 2009	81.3	17.2	135	80.6	17.8	134	31.4%	0.04 [-0.20, 0.28]
Andersen 2013	19.25	3.76	106	18.91	3.73	107	24.9%	0.09 [-0.18, 0.36]
Cormie 2013	47.8	11.2	22	48.4	12.6	19	4.8%	-0.05 [-0.66, 0.56]
Martin 2015 (a)	19.6	2.6	12	20.6	3.2	23	3.6%	-0.32 [-1.03, 0.38]
Martin 2015 (c)	20	3.5	26	20.3	2.8	25	6.0%	-0.09 [-0.64, 0.46]
Mijwel 2017	72.24	22.23	74	70.1	25.6	60	15.5%	0.09 [-0.25, 0.43]
Moller 2015 (a)	87.04	16.72	9	74.07	20.17	9	2.0%	0.67 [-0.29, 1.62]
Moller 2015 (b)	66.67	24.53	4	54.17	5.89	2	0.6%	0.47 [-1.29, 2.22]
Persoon 2017	86.2	16.3	50	86	14.7	47	11.3%	0.01 [-0.39, 0.41]
**Total (95% CI)**			**438**			**426**	**100.0%**	**0.05 [-0.09, 0.18]**

Heterogeneity: Tau² = 0.00; Chi² = 3.44, df = 8 (P = 0.90); I² = 0%
Test for overall effect: Z = 0.68 (P = 0.50)

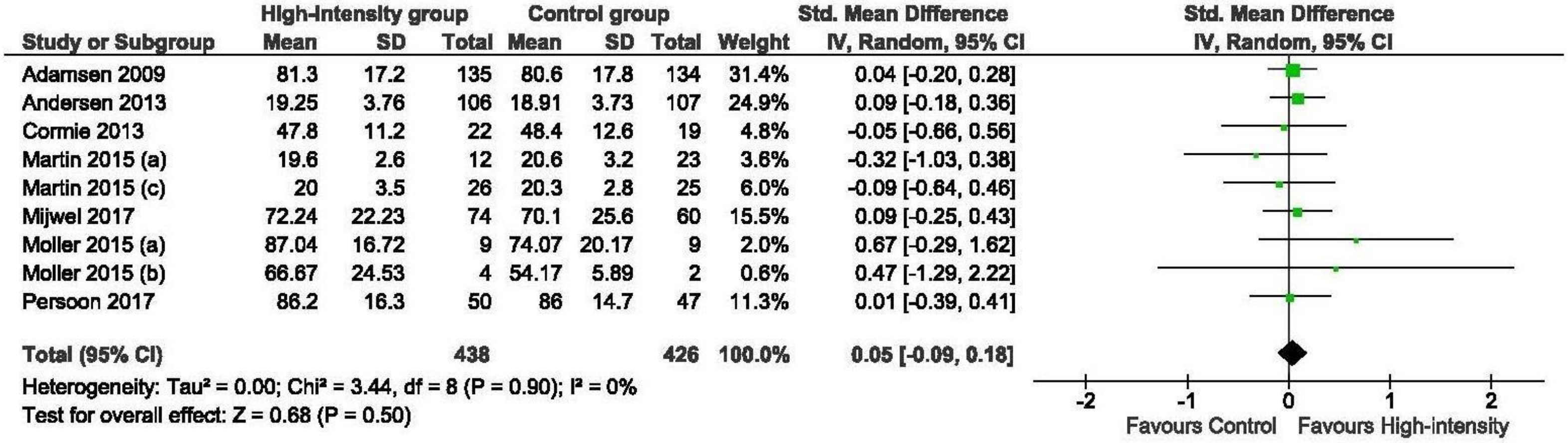

Supplementary Figure S36: Effects of the high-intensity training part duration in emotional functioning dimension.

Emotional functioning dimension: 15 minutes or les of high-intensity training

Study or Subgroup	High-intensity group Mean	SD	Total	Control group Mean	SD	Total	Weight	Std. Mean Difference IV, Random, 95% CI
Adamsen 2009	81.3	17.2	135	80.6	17.8	134	41.5%	0.04 [-0.20, 0.28]
Andersen 2013	19.25	3.76	106	18.91	3.73	107	32.8%	0.09 [-0.18, 0.36]
Egegaard 2019	18.9	3.8	8	20.2	3.1	5	1.9%	-0.34 [-1.47, 0.79]
Mijwel 2017	72.24	22.23	74	70.1	25.6	60	20.4%	0.09 [-0.25, 0.43]
Moller 2015 (a)	87.04	16.72	9	74.07	20.17	9	2.6%	0.67 [-0.29, 1.62]
Moller 2015 (b)	66.67	24.53	4	54.17	5.89	2	0.8%	0.47 [-1.29, 2.22]
**Total (95% CI)**			**336**			**317**	**100.0%**	**0.08 [-0.07, 0.23]**

Heterogeneity: Tau² = 0.00; Chi² = 2.28, df = 5 (P = 0.81); I² = 0%
Test for overall effect: Z = 1.01 (P = 0.31)

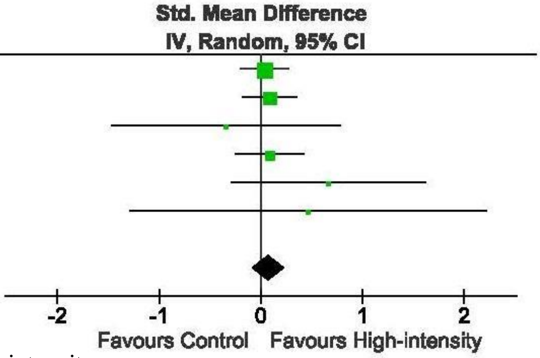

Emotional functioning dimension: More than 15 minutes of high-intensity

Study or Subgroup	High-intensity group Mean	SD	Total	Control group Mean	SD	Total	Weight	Std. Mean Difference IV, Random, 95% CI
Adams 2018	53.4	5.2	35	51	8.3	27	12.4%	0.35 [-0.15, 0.86]
Cormie 2013	47.8	11.2	22	48.4	12.6	19	8.4%	-0.05 [-0.66, 0.56]
Hwang 2012	91.7	12.3	13	85.6	13	11	4.8%	0.47 [-0.35, 1.28]
Kampshoff 2015	88.3	15.3	91	83.3	17.5	91	37.3%	0.30 [0.01, 0.60]
Martin 2015 (a)	19.6	2.6	12	20.6	3.2	23	6.4%	-0.32 [-1.03, 0.38]
Martin 2015 (c)	20	3.5	26	20.3	2.8	25	10.5%	-0.09 [-0.64, 0.46]
Persoon 2017	86.2	16.3	50	86	14.7	47	20.1%	0.01 [-0.39, 0.41]
**Total (95% CI)**			**249**			**243**	**100.0%**	**0.15 [-0.03, 0.33]**

Heterogeneity: Tau² = 0.00; Chi² = 5.61, df = 6 (P = 0.47); I² = 0%
Test for overall effect: Z = 1.61 (P = 0.11)

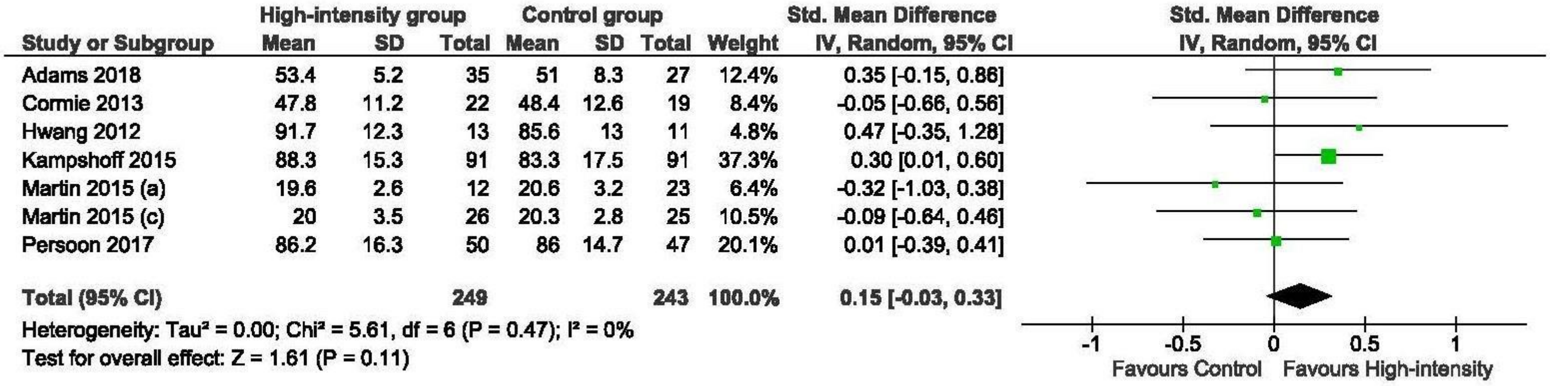

Supplementary Figure S37: Effects in the cognitive functioning dimension of the comparisons between high-intensity training group and control group, and high-intensity training group and low- intensity exercise group.

Cognitive functioning dimension: Comparison between high-intensity group and control group

Study or Subgroup	High-intensity group Mean	SD	Total	Control group Mean	SD	Total	Weight	Mean Difference IV, Random, 95% CI
Adamsen 2009	83.8	16.7	135	81.3	19.8	134	32.6%	2.50 [-1.88, 6.88]
Hwang 2012	87.2	13.9	13	84.8	11.7	11	6.0%	2.40 [-7.84, 12.64]
Kampshoff 2015	84.3	17.5	91	80.6	21.1	91	19.9%	3.70 [-1.93, 9.33]
Mijwel 2017	78.29	20.13	74	70.76	26.65	60	9.5%	7.53 [-0.63, 15.69]
Moller 2015 (a)	79.63	13.89	9	77.78	8.33	9	5.7%	1.85 [-8.73, 12.43]
Moller 2015 (b)	75	21.52	4	50	0.01	2	1.4%	25.00 [3.91, 46.09]
Persoon 2017	83.7	18	50	82.3	20.4	47	10.7%	1.40 [-6.28, 9.08]
Van Wart 2015	78.2	19	76	70.2	23.1	77	14.1%	8.00 [1.30, 14.70]
**Total (95% CI)**			**452**			**431**	**100.0%**	**4.15 [1.63, 6.67]**

Heterogeneity: Tau² = 0.08; Chi² = 7.04, df = 7 (P = 0.42); I² = 1%
Test for overall effect: Z = 3.23 (P = 0.001)

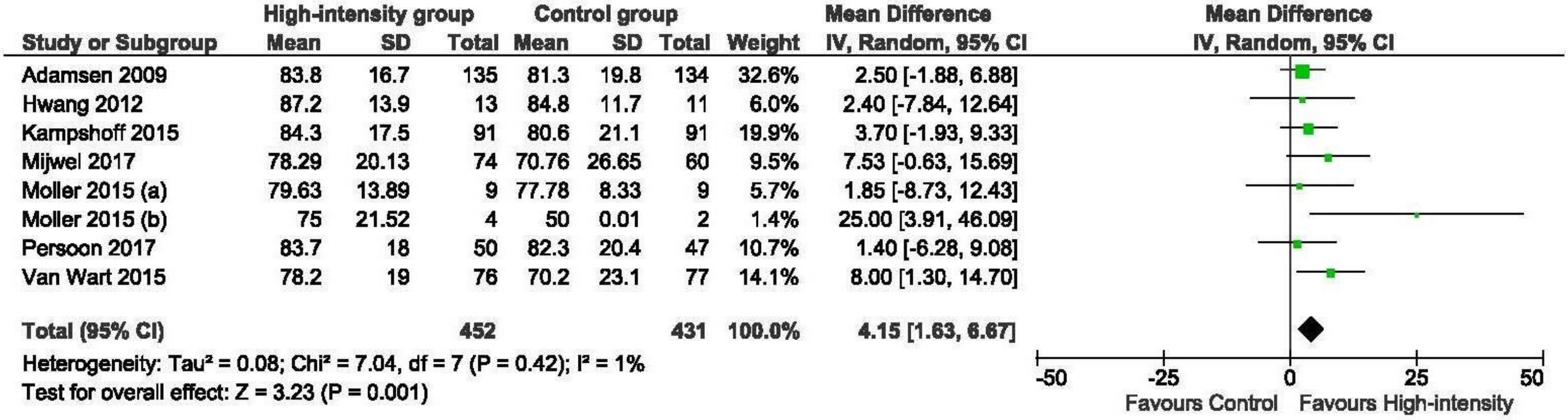

Cognitive functioning dimension: Comparison between high-intensity group and low-moderate intensity

Study or Subgroup	High-intentity group Mean	SD	Total	Active group Mean	SD	Total	Weight	Mean Difference IV, Random, 95% CI
Kampshoff 2015	84.3	17.5	91	80.3	19.5	95	56.2%	4.00 [-1.32, 9.32]
Moller 2015 (a)	79.63	13.89	9	70.37	27.36	9	4.0%	9.26 [-10.79, 29.31]
Moller 2015 (b)	75	21.52	4	83.33	0.01	3	3.6%	-8.33 [-29.42, 12.76]
Schmitt 2016	69.2	29.5	13	73.1	24	13	3.7%	-3.90 [-24.57, 16.77]
Van Wart 2015	78.2	19	76	73.6	24.8	77	32.5%	4.60 [-2.40, 11.60]
**Total (95% CI)**			**193**			**197**	**100.0%**	**3.67 [-0.32, 7.66]**

Heterogeneity: Tau² = 0.00; Chi² = 2.14, df = 4 (P = 0.71); I² = 0%
Test for overall effect: Z = 1.80 (P = 0.07)

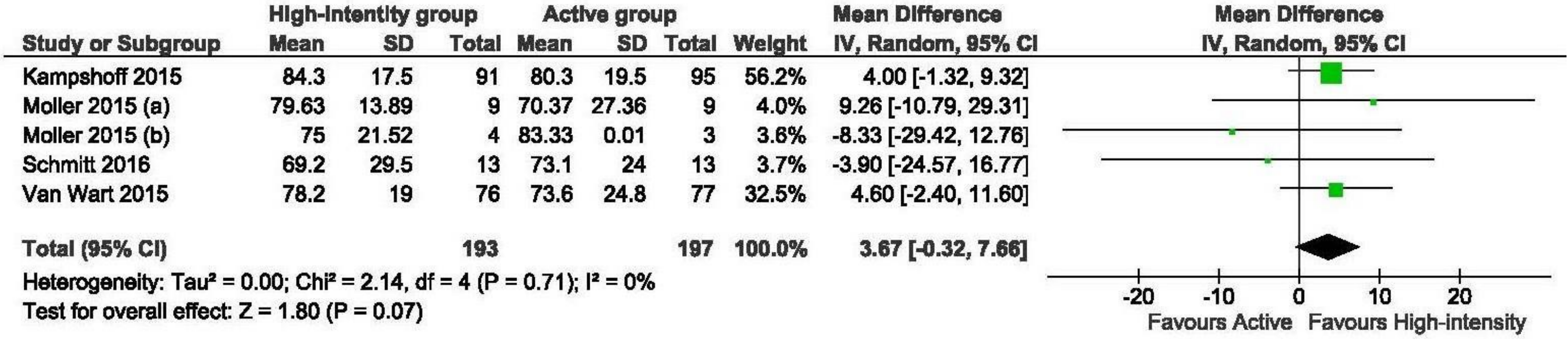

Supplementary Figure S38: Effects of the exercise programs length in global health dimension.

Cognitive functioning dimension: Eight weeks of exercise program or less

	High-intensity group			Control group				Std. Mean Difference
Study or Subgroup	Mean	SD	Total	Mean	SD	Total	Weight	IV, Random, 95% CI
Adamsen 2009	83.8	16.7	135	81.3	19.8	134	85.5%	0.14 [-0.10, 0.38]
Hwang 2012	87.2	13.9	13	84.8	11.7	11	7.6%	0.18 [-0.63, 0.98]
Moller 2015 (a)	79.63	13.89	9	77.78	8.33	9	5.7%	0.15 [-0.77, 1.08]
Moller 2015 (b)	75	21.52	4	50	0.01	2	1.2%	1.07 [-0.92, 3.06]
**Total (95% CI)**			**161**			**156**	**100.0%**	**0.15 [-0.07, 0.37]**

Heterogeneity: Tau² = 0.00; Chi² = 0.85, df = 3 (P = 0.84); I² = 0%
Test for overall effect: Z = 1.35 (P = 0.18)

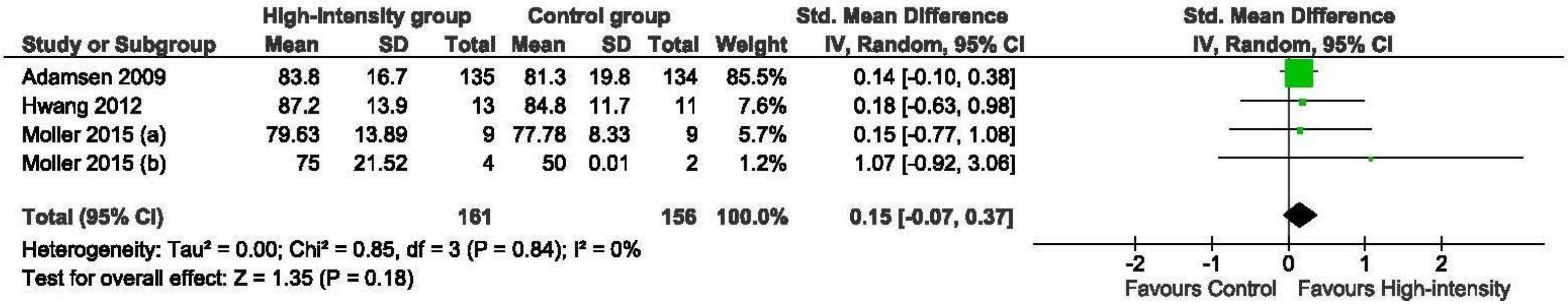

Cognitive functioning dimension: More than eight weeks of exercise program

	High-intensity group			Control group				Std. Mean Difference
Study or Subgroup	Mean	SD	Total	Mean	SD	Total	Weight	IV, Random, 95% CI
Kampshoff 2015	84.3	17.5	91	80.6	21.1	91	32.4%	0.19 [-0.10, 0.48]
Mijwel 2017	78.29	20.13	74	70.76	26.65	60	23.4%	0.32 [-0.02, 0.66]
Persoon 2017	83.7	18	50	82.3	20.4	47	17.3%	0.07 [-0.33, 0.47]
Van Wart 2015	78.2	19	76	70.2	23.1	77	26.9%	0.38 [0.06, 0.70]
**Total (95% CI)**			**291**			**275**	**100.0%**	**0.25 [0.08, 0.42]**

Heterogeneity: Tau² = 0.00; Chi² = 1.69, df = 3 (P = 0.64); I² = 0%
Test for overall effect: Z = 2.96 (P = 0.003)

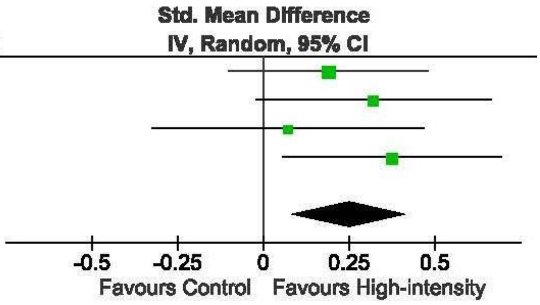

Supplementary Figure S39: Effects of the weekly exercise frequency in cognitive functioning dimension.

Cognitive functioning dimension: 2 times per week

	High-intensity group			Control group				Std. Mean Difference
Study or Subgroup	Mean	SD	Total	Mean	SD	Total	Weight	IV, Random, 95% CI
Kampshoff 2015	84.3	17.5	91	80.6	21.1	91	44.3%	0.19 [-0.10, 0.48]
Mijwel 2017	78.29	20.13	74	70.76	26.65	60	32.0%	0.32 [-0.02, 0.66]
Persoon 2017	83.7	18	50	82.3	20.4	47	23.7%	0.07 [-0.33, 0.47]
**Total (95% CI)**			**215**			**198**	**100.0%**	**0.20 [0.01, 0.40]**

Heterogeneity: Tau² = 0.00; Chi² = 0.88, df = 2 (P = 0.64); I² = 0%
Test for overall effect: Z = 2.07 (P = 0.04)

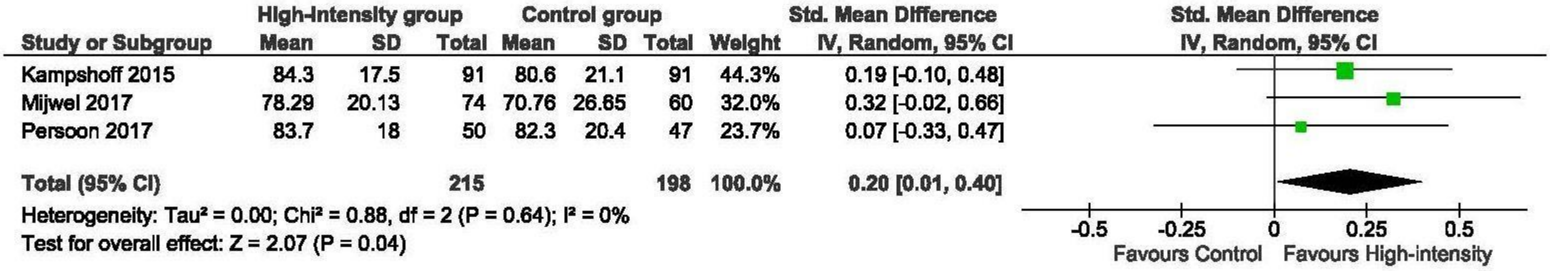

Cognitive functioning dimension: 3 times per week

	High-intensity group			Control group				Std. Mean Difference
Study or Subgroup	Mean	SD	Total	Mean	SD	Total	Weight	IV, Random, 95% CI
Adamsen 2009	83.8	16.7	135	81.3	19.8	134	85.5%	0.14 [-0.10, 0.38]
Hwang 2012	87.2	13.9	13	84.8	11.7	11	7.6%	0.18 [-0.63, 0.98]
Moller 2015 (a)	79.63	13.89	9	77.78	8.33	9	5.7%	0.15 [-0.77, 1.08]
Moller 2015 (b)	75	21.52	4	50	0.01	2	1.2%	1.07 [-0.92, 3.06]
**Total (95% CI)**			**161**			**156**	**100.0%**	**0.15 [-0.07, 0.37]**

Heterogeneity: Tau² = 0.00; Chi² = 0.85, df = 3 (P = 0.84); I² = 0%
Test for overall effect: Z = 1.35 (P = 0.18)

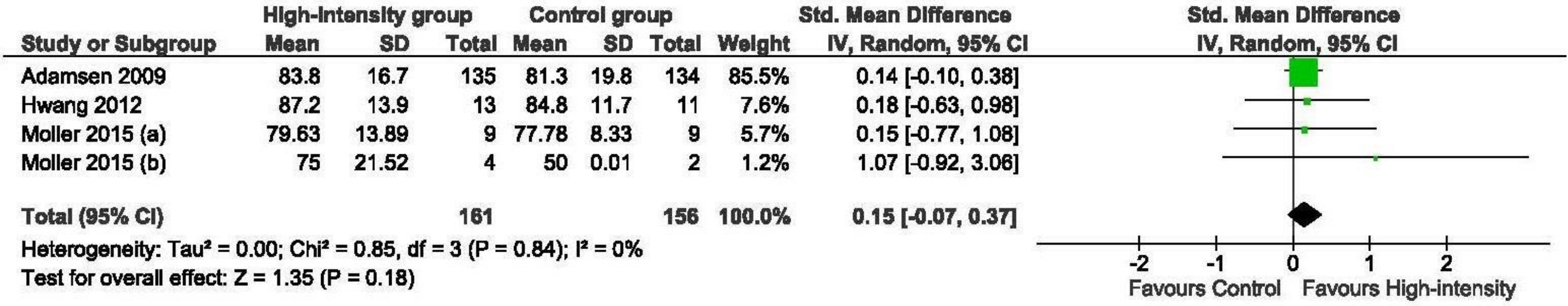

Supplementary Figure S40: Effects of the high-intensity training part duration in cognitive functioning dimension.

Cognitive functioning dimension: 15 minutes or les of high-intensity training

	High-intensity group			Control group				Std. Mean Difference
Study or Subgroup	Mean	SD	Total	Mean	SD	Total	Weight	IV, Random, 95% CI
Adamsen 2009	83.8	16.7	135	81.3	19.8	134	63.7%	0.14 [-0.10, 0.38]
Mijwel 2017	78.29	20.13	74	70.76	26.65	60	31.1%	0.32 [-0.02, 0.66]
Moller 2015 (a)	79.63	13.89	9	77.78	8.33	9	4.3%	0.15 [-0.77, 1.08]
Moller 2015 (b)	75	21.52	4	50	0.01	2	0.9%	1.07 [-0.92, 3.06]
**Total (95% CI)**			**222**			**205**	**100.0%**	**0.20 [0.01, 0.39]**

Heterogeneity: Tau² = 0.00; Chi² = 1.51, df = 3 (P = 0.68); I² = 0%
Test for overall effect: Z = 2.08 (P = 0.04)

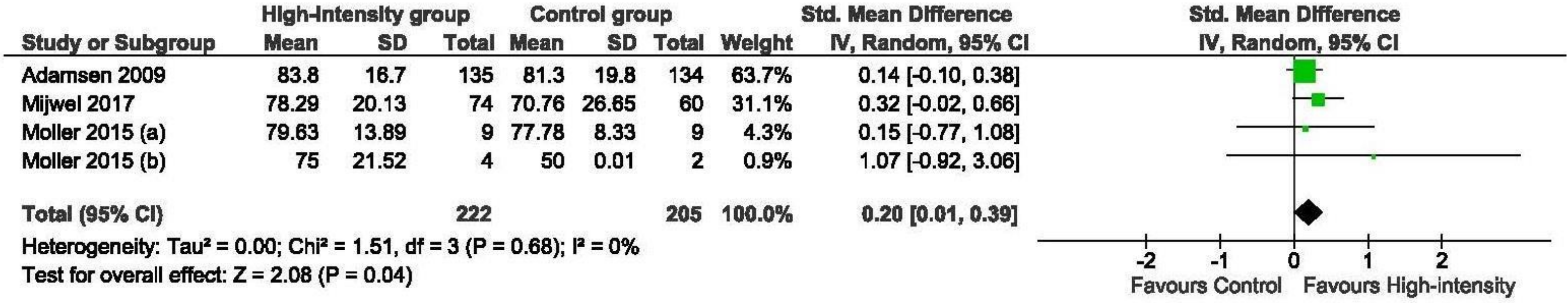

Cognitive functioning dimension: More than 15 minutes of high-intensity training

	High-intensity group			Control group				Std. Mean Difference
Study or Subgroup	Mean	SD	Total	Mean	SD	Total	Weight	IV, Random, 95% CI
Hwang 2012	87.2	13.9	13	84.8	11.7	11	5.2%	0.18 [-0.63, 0.98]
Kampshoff 2015	84.3	17.5	91	80.6	21.1	91	40.1%	0.19 [-0.10, 0.48]
Persoon 2017	83.7	18	50	82.3	20.4	47	21.4%	0.07 [-0.33, 0.47]
Van Wart 2015	78.2	19	76	70.2	23.1	77	33.2%	0.38 [0.06, 0.70]
**Total (95% CI)**			**230**			**226**	**100.0%**	**0.23 [0.04, 0.41]**

Heterogeneity: Tau² = 0.00; Chi² = 1.49, df = 3 (P = 0.68); I² = 0%
Test for overall effect: Z = 2.40 (P = 0.02)

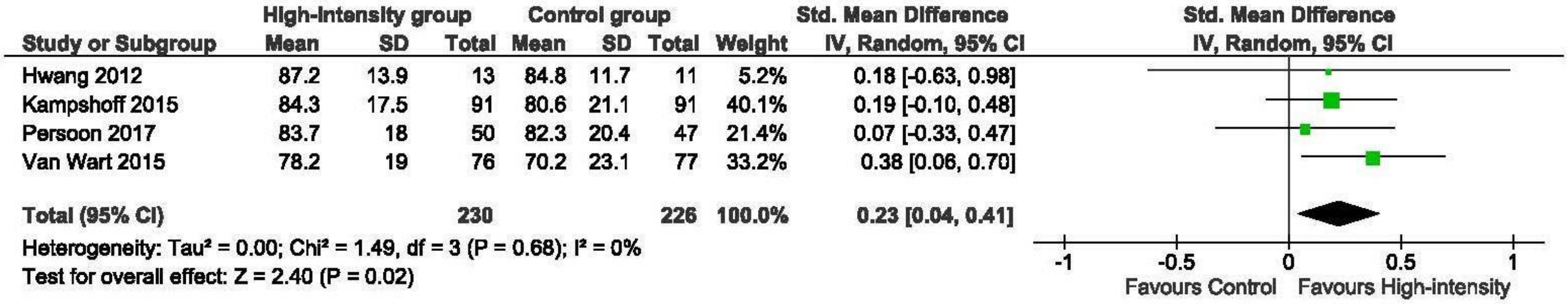

Supplementary Figure S41: Effects in social functioning dimension of the comparisons between high-intensity training group and control group, and high- intensity training group and low- intensity exercise group.

Social functioning dimension: Comparison between high-intensity group and control group

	High-intensity group			Control group				Std. Mean Difference
Study or Subgroup	Mean	SD	Total	Mean	SD	Total	Weight	IV, Random, 95% CI
Adams 2018	45	5.1	35	42.6	7.4	27	4.7%	0.38 [-0.12, 0.89]
Adamsen 2009	82.6	20.5	135	79.4	20.8	134	20.9%	0.15 [-0.08, 0.39]
Andersen 2013	23.25	3.84	106	23.27	3.73	107	16.6%	-0.01 [-0.27, 0.26]
Cormie 2013	48.2	8.4	22	48.5	12.1	19	3.2%	-0.03 [-0.64, 0.59]
Egegaard 2019	23.3	5.8	8	24.2	3.8	5	1.0%	-0.16 [-1.28, 0.96]
Hwang 2012	91	12.9	13	87.8	21.8	11	1.8%	0.18 [-0.63, 0.98]
Kampshoff 2015	89.6	15.1	91	85.2	21.7	91	14.1%	0.23 [-0.06, 0.53]
Martin 2015 (a)	24	3.5	12	22.1	5.4	23	2.4%	0.38 [-0.32, 1.09]
Martin 2015 (c)	20.2	3.6	26	21.5	4.4	25	3.9%	-0.32 [-0.87, 0.23]
Mijwel 2017	62.32	46.87	74	63.3	27.67	60	10.3%	-0.02 [-0.37, 0.32]
Moller 2015 (a)	87.04	16.2	9	74.07	14.7	9	1.3%	0.80 [-0.17, 1.77]
Moller 2015 (b)	75	31.91	4	66.67	47.14	2	0.4%	0.18 [-1.52, 1.89]
Persoon 2017	86	20.3	50	83.7	20.4	47	7.5%	0.11 [-0.29, 0.51]
Van Wart 2015	73.5	21.6	76	67.9	29.1	77	11.9%	0.22 [-0.10, 0.54]
**Total (95% CI)**			**661**			**637**	**100.0%**	**0.12 [0.01, 0.23]**

Heterogeneity: Tau² = 0.00; Chi² = 8.92, df = 13 (P = 0.78); I² = 0%
Test for overall effect: Z = 2.19 (P = 0.03)

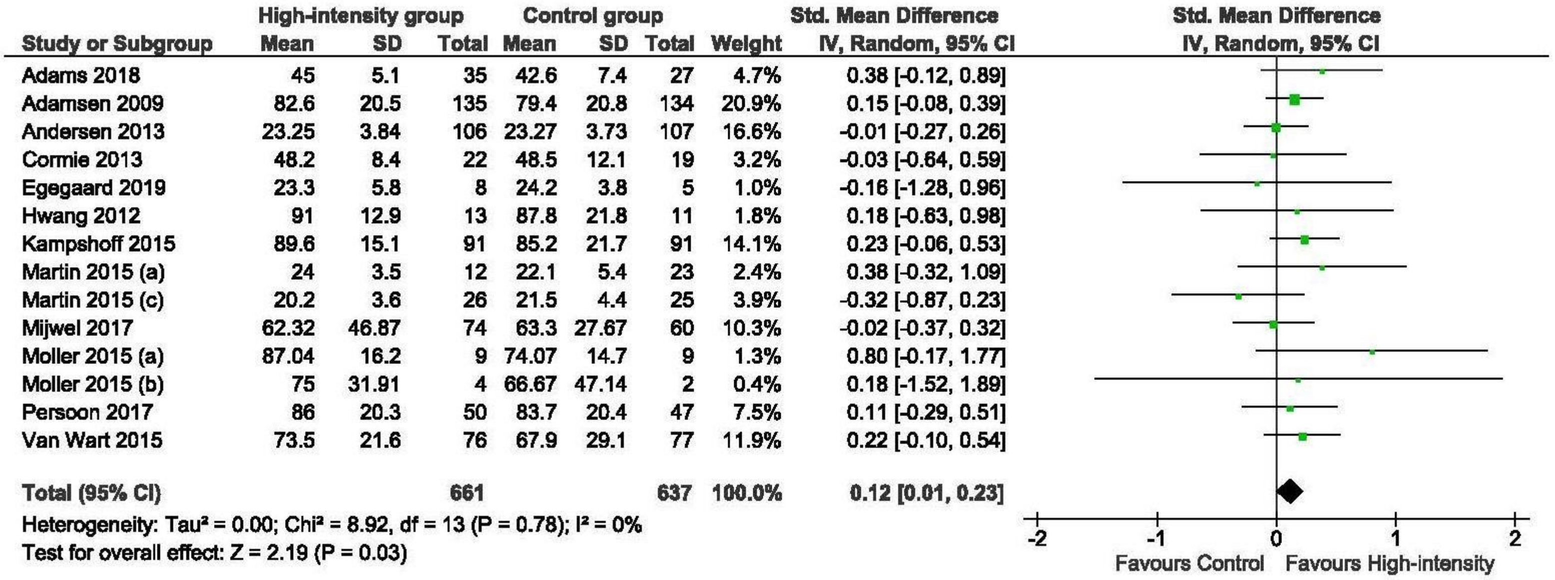

Social functioning dimension: Comparison between high-intensity group and low-moderate intensity group

	High-intensity group			Control group				Std. Mean Difference
Study or Subgroup	Mean	SD	Total	Mean	SD	Total	Weight	IV, Random, 95% CI
Adams 2018	45	5.1	35	42.6	7.4	27	13.0%	0.38 [-0.12, 0.89]
Cormie 2013	48.2	8.4	22	48.5	12.1	19	8.9%	-0.03 [-0.64, 0.59]
Kampshoff 2015	89.6	15.1	91	85.2	21.7	91	39.3%	0.23 [-0.06, 0.53]
Martin 2015 (a)	24	3.5	12	22.1	5.4	23	6.7%	0.38 [-0.32, 1.09]
Martin 2015 (c)	20.2	3.6	26	21.5	4.4	25	10.9%	-0.32 [-0.87, 0.23]
Persoon 2017	86	20.3	50	83.7	20.4	47	21.1%	0.11 [-0.29, 0.51]
**Total (95% CI)**			**236**			**232**	**100.0%**	**0.15 [-0.03, 0.34]**

Heterogeneity: Tau² = 0.00; Chi² = 4.67, df = 5 (P = 0.46); I² = 0%
Test for overall effect: Z = 1.65 (P = 0.10)

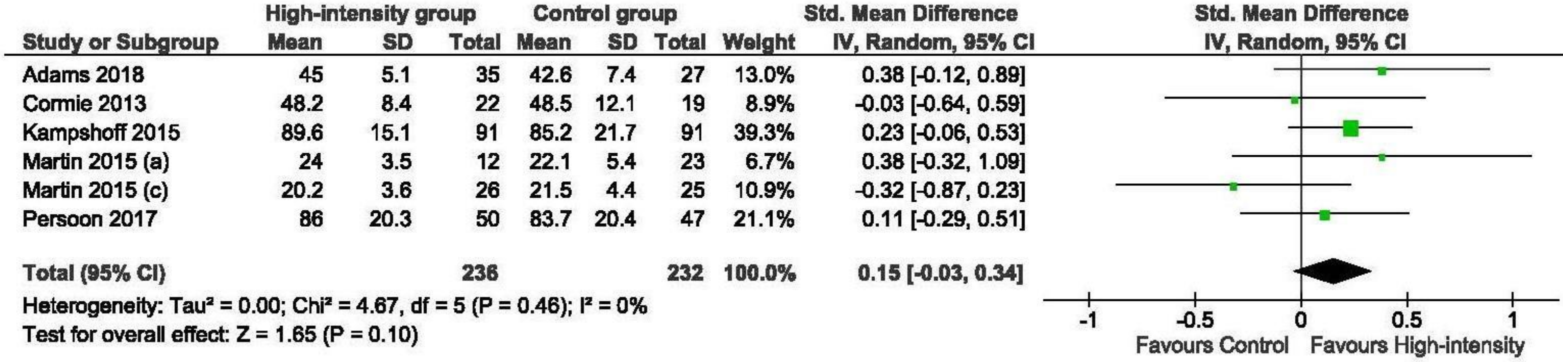

Supplementary Figure S42: Effects of high-intensity training in social functioning according to the treatment timing.

Social functioning dimension: During treatment

Study or Subgroup	High-intensity group Mean	SD	Total	Control group Mean	SD	Total	Weight	Std. Mean Difference IV, Random, 95% CI
Adamsen 2009	45	5.1	35	42.6	7.4	27	8.9%	0.38 [-0.12, 0.89]
Andersen 2013	82.6	20.5	135	79.4	20.8	134	40.0%	0.15 [-0.08, 0.39]
Egegaard 2019	23.3	5.8	8	24.2	3.8	5	1.8%	-0.16 [-1.28, 0.96]
Hwang 2012	91	12.9	13	87.8	21.8	11	3.5%	0.18 [-0.63, 0.98]
Mijwel 2017	62.32	46.87	74	63.3	27.67	60	19.8%	-0.02 [-0.37, 0.32]
Moller 2015 (a)	87.04	16.2	9	74.07	14.7	9	2.4%	0.80 [-0.17, 1.77]
Moller 2015 (b)	75	31.91	4	66.67	47.14	2	0.8%	0.18 [-1.52, 1.89]
Van Wart 2015	73.5	21.6	76	67.9	29.1	77	22.7%	0.22 [-0.10, 0.54]
**Total (95% CI)**			**354**			**325**	**100.0%**	**0.16 [0.01, 0.32]**

Heterogeneity: Tau² = 0.00; Chi² = 3.98, df = 7 (P = 0.78); I² = 0%
Test for overall effect: Z = 2.13 (P = 0.03)

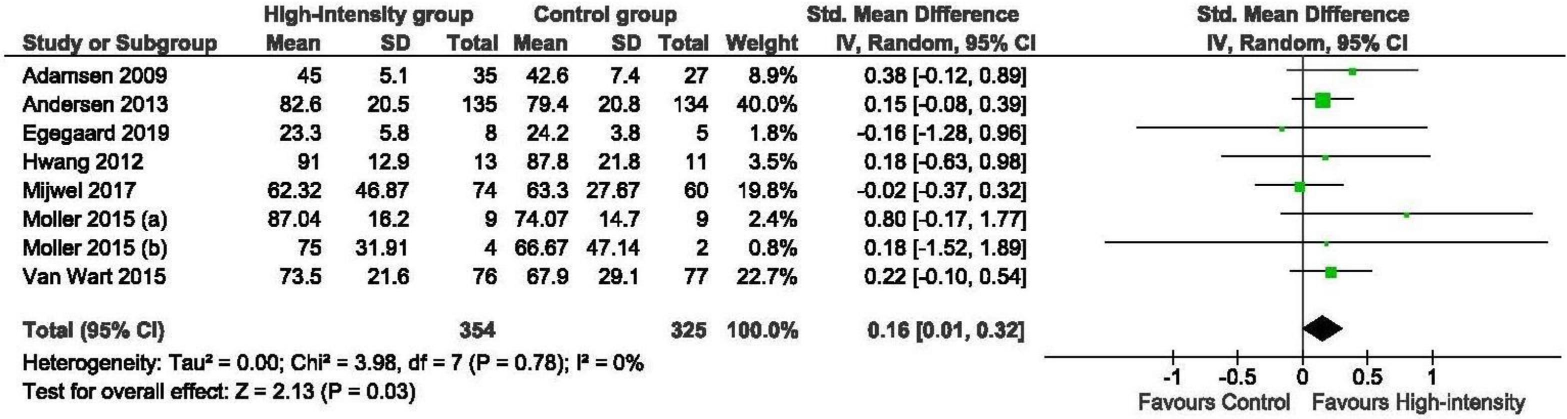

Social functioning dimension: After treatment

Study or Subgroup	High-intensity group Mean	SD	Total	Control group Mean	SD	Total	Weight	Std. Mean Difference IV, Random, 95% CI
Adams 2018	45	5.1	35	42.6	7.4	27	13.0%	0.38 [-0.12, 0.89]
Cormie 2013	48.2	8.4	22	48.5	12.1	19	8.9%	-0.03 [-0.64, 0.59]
Kampshoff 2015	89.6	15.1	91	85.2	21.7	91	39.3%	0.23 [-0.06, 0.53]
Martin 2015 (a)	24	3.5	12	22.1	5.4	23	6.7%	0.38 [-0.32, 1.09]
Martin 2015 (c)	20.2	3.6	26	21.5	4.4	25	10.9%	-0.32 [-0.87, 0.23]
Persoon 2017	86	20.3	50	83.7	20.4	47	21.1%	0.11 [-0.29, 0.51]
**Total (95% CI)**			**236**			**232**	**100.0%**	**0.15 [-0.03, 0.34]**

Heterogeneity: Tau² = 0.00; Chi² = 4.67, df = 5 (P = 0.46); I² = 0%
Test for overall effect: Z = 1.65 (P = 0.10)

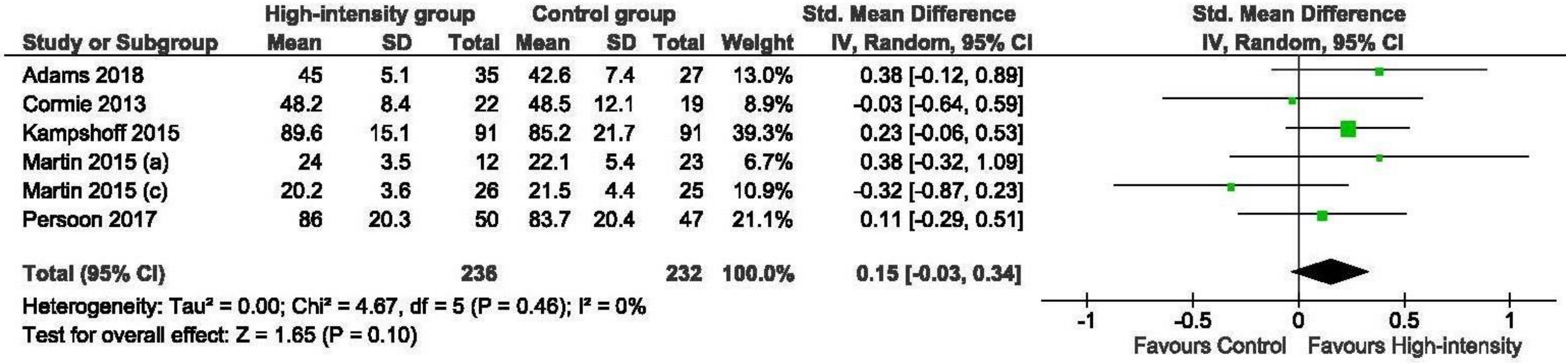

Supplementary Figure S43: Effects of the type of exercise in social functioning dimension.

Social functioning dimension: High-intensity cardiovascular training

Study or Subgroup	High-intensity group Mean	SD	Total	Control group Mean	SD	Total	Weight	Std. Mean Difference IV, Random, 95% CI
Adams 2018	45	5.1	35	42.6	7.4	27	26.8%	0.38 [-0.12, 0.89]
Egegaard 2019	23.3	5.8	8	24.2	3.8	5	5.5%	-0.16 [-1.28, 0.96]
Hwang 2012	91	12.9	13	87.8	21.8	11	10.6%	0.18 [-0.63, 0.98]
Mijwel 2017	71.33	23.94	70	63.3	27.67	60	57.1%	0.31 [-0.04, 0.66]
**Total (95% CI)**			**126**			**103**	**100.0%**	**0.29 [0.03, 0.55]**

Heterogeneity: Tau² = 0.00; Chi² = 0.84, df = 3 (P = 0.84); I² = 0%
Test for overall effect: Z = 2.16 (P = 0.03)

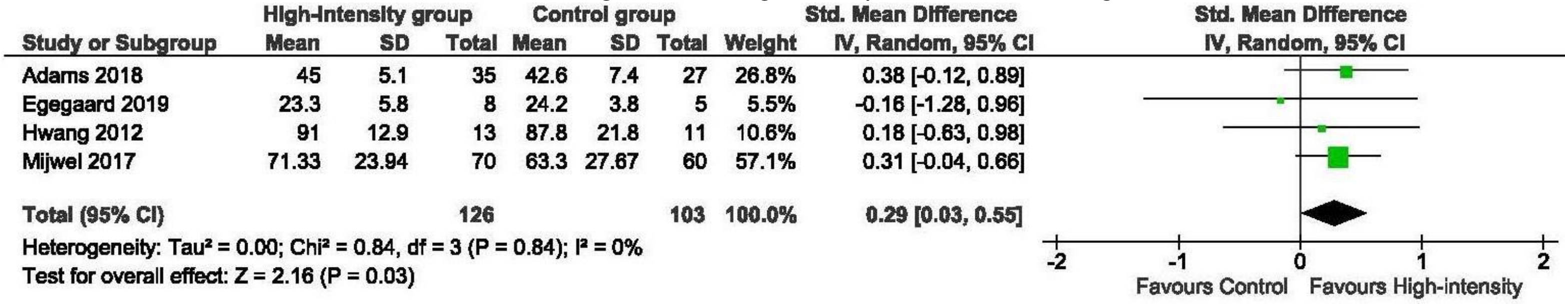

Social functioning dimension: High-intensity cardiovascular training + Resistance training

Study or Subgroup	High-intensity group Mean	SD	Total	Control group Mean	SD	Total	Weight	Std. Mean Difference IV, Random, 95% CI
Adamsen 2009	82.6	20.5	135	79.4	20.8	134	22.6%	0.15 [-0.08, 0.39]
Andersen 2013	23.25	3.84	106	23.27	3.73	107	17.9%	-0.01 [-0.27, 0.26]
Cormie 2013	48.2	8.4	22	48.5	12.1	19	3.4%	-0.03 [-0.64, 0.59]
Kampshoff 2015	89.6	15.1	91	85.2	21.7	91	15.2%	0.23 [-0.06, 0.53]
Martin 2015 (a)	24	3.5	12	22.1	5.4	23	2.6%	0.38 [-0.32, 1.09]
Martin 2015 (c)	20.2	3.6	26	21.5	4.4	25	4.2%	-0.32 [-0.87, 0.23]
Mijwel 2017	62.32	46.87	74	63.3	27.67	60	11.2%	-0.02 [-0.37, 0.32]
Moller 2015 (a)	87.04	16.2	9	74.07	14.7	9	1.4%	0.80 [-0.17, 1.77]
Moller 2015 (b)	75	31.91	4	66.67	47.14	2	0.4%	0.18 [-1.52, 1.89]
Persoon 2017	86	20.3	50	83.7	20.4	47	8.2%	0.11 [-0.29, 0.51]
Van Wart 2015	73.5	21.6	76	67.9	29.1	77	12.8%	0.22 [-0.10, 0.54]
**Total (95% CI)**			**605**			**594**	**100.0%**	**0.11 [-0.00, 0.22]**

Heterogeneity: Tau² = 0.00; Chi² = 7.61, df = 10 (P = 0.67); I² = 0%
Test for overall effect: Z = 1.91 (P = 0.06)

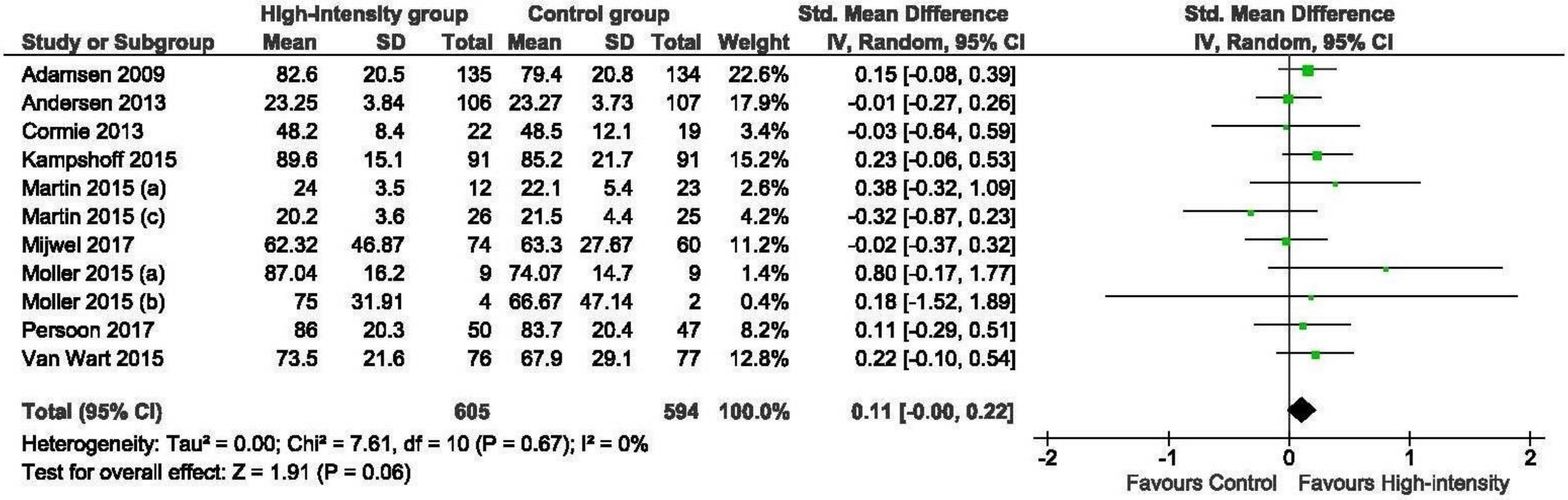

Supplementary Figure S44: Effects of the exercise programs length in social functioning dimension.

Social functioning dimension: Eight weeks of exercise program or less

Study or Subgroup	High-intensity group Mean	SD	Total	Control group Mean	SD	Total	Weight	Std. Mean Difference IV, Random, 95% CI
Adamsen 2009	82.6	20.5	135	79.4	20.8	134	43.3%	0.15 [-0.08, 0.39]
Andersen 2013	23.25	3.84	106	23.27	3.73	107	34.4%	-0.01 [-0.27, 0.26]
Egegaard 2019	23.3	5.8	8	24.2	3.8	5	2.0%	-0.16 [-1.28, 0.96]
Hwang 2012	91	12.9	13	87.8	21.8	11	3.8%	0.18 [-0.63, 0.98]
Martin 2015 (a)	24	3.5	12	22.1	5.4	23	5.0%	0.38 [-0.32, 1.09]
Martin 2015 (c)	20.2	3.6	26	21.5	4.4	25	8.1%	-0.32 [-0.87, 0.23]
Moller 2015 (a)	87.04	16.2	9	74.07	14.7	9	2.6%	0.80 [-0.17, 1.77]
Moller 2015 (b)	75	31.91	4	66.67	47.14	2	0.9%	0.18 [-1.52, 1.89]
**Total (95% CI)**			**313**			**316**	**100.0%**	**0.08 [-0.07, 0.24]**

Heterogeneity: Tau² = 0.00; Chi² = 5.83, df = 7 (P = 0.56); I² = 0%
Test for overall effect: Z = 1.05 (P = 0.29)

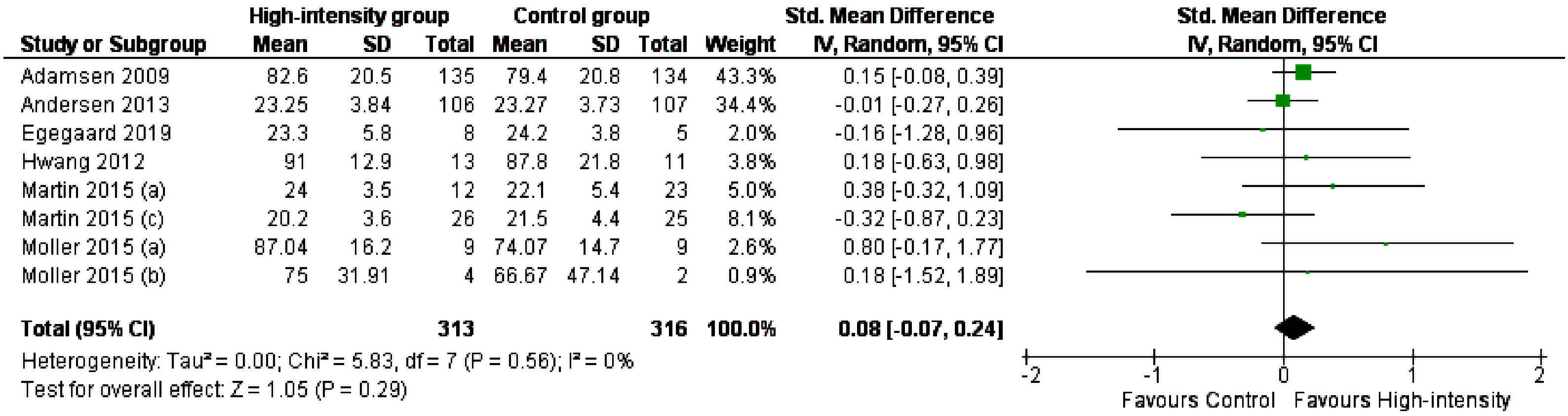

Social functioning dimension: More than eight weeks of exercise program

Study or Subgroup	High-intensity group Mean	SD	Total	Control group Mean	SD	Total	Weight	Std. Mean Difference IV, Random, 95% CI
Adams 2018	45	5.1	35	42.6	7.4	27	11.7%	0.38 [-0.12, 0.89]
Cormie 2013	48.2	8.4	22	48.5	12.1	19	8.0%	-0.03 [-0.64, 0.59]
Kampshoff 2015	89.6	15.1	91	85.2	21.7	91	35.4%	0.23 [-0.06, 0.53]
Mijwel 2017	62.32	46.87	74	63.3	27.67	60	26.0%	-0.02 [-0.37, 0.32]
Persoon 2017	86	20.3	50	83.7	20.4	47	18.9%	0.11 [-0.29, 0.51]
**Total (95% CI)**			**272**			**244**	**100.0%**	**0.14 [-0.03, 0.31]**

Heterogeneity: Tau² = 0.00; Chi² = 2.49, df = 4 (P = 0.65); I² = 0%
Test for overall effect: Z = 1.58 (P = 0.11)

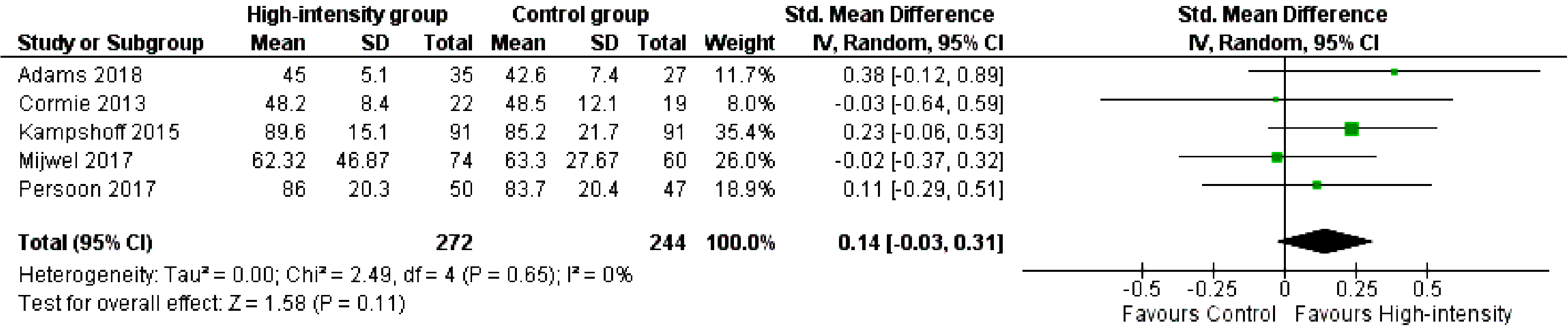

Supplementary Figure S45: Effects of the weekly exercise frequency in social functioning dimension

Social functioning dimension: 2 times per week

Study or Subgroup	High-intensity group Mean	SD	Total	Control group Mean	SD	Total	Weight	Std. Mean Difference IV, Random, 95% CI
Cormie 2013	48.2	8.4	22	48.5	12.1	19	6.8%	-0.03 [-0.64, 0.59]
Kampshoff 2015	89.6	15.1	91	85.2	21.7	91	30.0%	0.23 [-0.06, 0.53]
Mijwel 2017	62.32	46.87	74	63.3	27.67	60	22.0%	-0.02 [-0.37, 0.32]
Persoon 2017	86	20.3	50	83.7	20.4	47	16.0%	0.11 [-0.29, 0.51]
Van Wart 2015	73.5	21.6	76	67.9	29.1	77	25.2%	0.22 [-0.10, 0.54]
**Total (95% CI)**			**313**			**294**	**100.0%**	**0.14 [-0.02, 0.30]**

Heterogeneity: Tau² = 0.00; Chi² = 1.83, df = 4 (P = 0.77); I² = 0%
Test for overall effect: Z = 1.67 (P = 0.10)

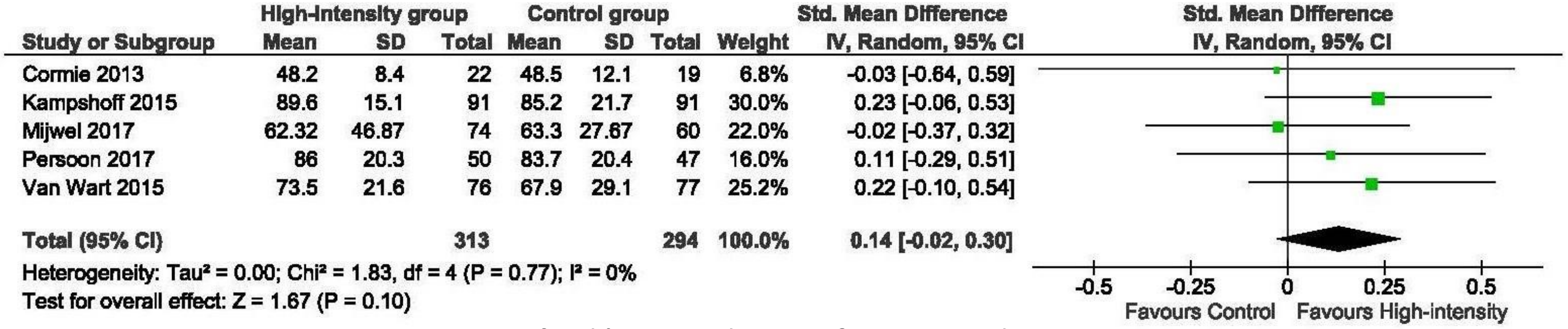

Social functioning dimension: 3 times per week

Study or Subgroup	High-intensity group Mean	SD	Total	Control group Mean	SD	Total	Weight	Std. Mean Difference IV, Random, 95% CI
Adams 2018	45	5.1	35	42.6	7.4	27	9.0%	0.38 [-0.12, 0.89]
Adamsen 2009	82.6	20.5	135	79.4	20.8	134	40.2%	0.15 [-0.08, 0.39]
Andersen 2013	23.25	3.84	106	23.27	3.73	107	31.9%	-0.01 [-0.27, 0.26]
Hwang 2012	91	12.9	13	87.8	21.8	11	3.6%	0.18 [-0.63, 0.98]
Martin 2015 (a)	24	3.5	12	22.1	5.4	23	4.6%	0.38 [-0.32, 1.09]
Martin 2015 (c)	20.2	3.6	26	21.5	4.4	25	7.5%	-0.32 [-0.87, 0.23]
Moller 2015 (a)	87.04	16.2	9	74.07	14.7	9	2.4%	0.80 [-0.17, 1.77]
Moller 2015 (b)	75	31.91	4	66.67	47.14	2	0.8%	0.18 [-1.52, 1.89]
**Total (95% CI)**			**340**			**338**	**100.0%**	**0.12 [-0.04, 0.27]**

Heterogeneity: Tau² = 0.00; Chi² = 6.80, df = 7 (P = 0.45); I² = 0%
Test for overall effect: Z = 1.49 (P = 0.14)

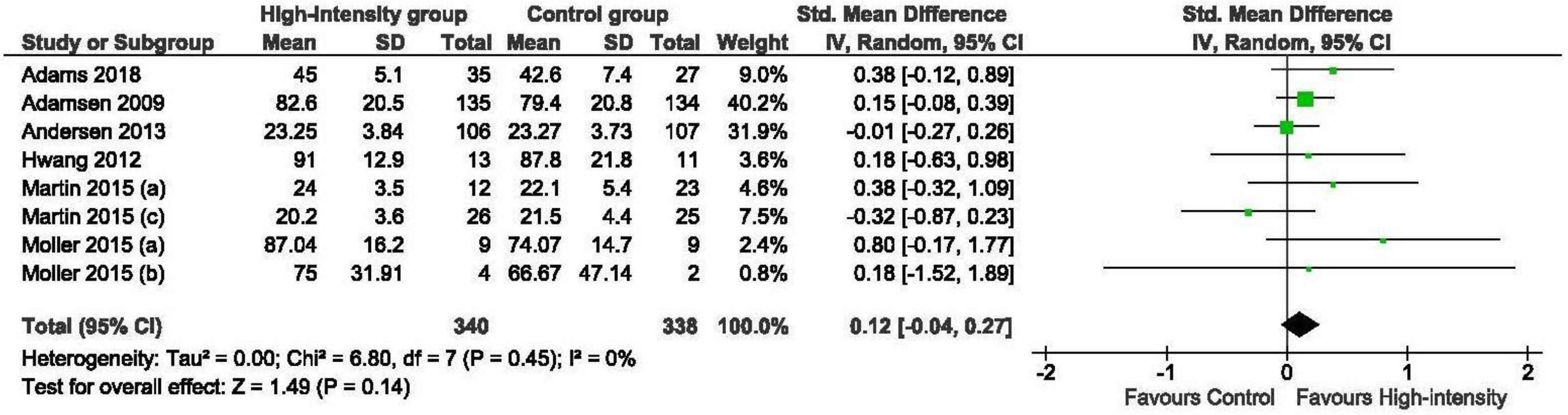

.

Supplementary Figure S46: Effects of training minutes per week in social functioning dimension.

Social functioning dimension: Less than 120 minutes of exercise per weeks

Study or Subgroup	High-intensity group Mean	SD	Total	Control group Mean	SD	Total	Weight	Std. Mean Difference IV, Random, 95% CI
Adams 2018	45	5.1	35	42.6	7.4	27	24.1%	0.38 [-0.12, 0.89]
Egegaard 2019	23.3	5.8	8	24.2	3.8	5	4.9%	-0.16 [-1.28, 0.96]
Hwang 2012	91	12.9	13	87.8	21.8	11	9.6%	0.18 [-0.63, 0.98]
Van Wart 2015	73.5	21.6	76	67.9	29.1	77	61.4%	0.22 [-0.10, 0.54]
**Total (95% CI)**			**132**			**120**	**100.0%**	**0.23 [-0.01, 0.48]**

Heterogeneity: Tau² = 0.00; Chi² = 0.84, df = 3 (P = 0.84); I² = 0%
Test for overall effect: Z = 1.84 (P = 0.07)

Std. Mean Difference IV, Random, 95% CI

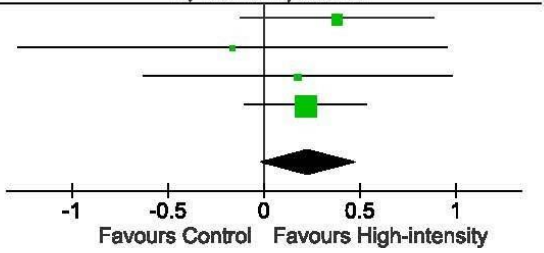

Social functioning dimension:120 minutes or more or exercise per weeks

Study or Subgroup	High-intensity group Mean	SD	Total	Control group Mean	SD	Total	Weight	Std. Mean Difference IV, Random, 95% CI
Adamsen 2009	82.6	20.5	135	79.4	20.8	134	31.4%	0.15 [-0.08, 0.39]
Andersen 2013	23.25	3.84	106	23.27	3.73	107	24.9%	-0.01 [-0.27, 0.26]
Cormie 2013	48.2	8.4	22	48.5	12.1	19	4.8%	-0.03 [-0.64, 0.59]
Martin 2015 (a)	24	3.5	12	22.1	5.4	23	3.6%	0.38 [-0.32, 1.09]
Martin 2015 (c)	20.2	3.6	26	21.5	4.4	25	5.9%	-0.32 [-0.87, 0.23]
Mijwel 2017	62.32	46.87	74	63.3	27.67	60	15.5%	-0.02 [-0.37, 0.32]
Moller 2015 (a)	87.04	16.2	9	74.07	14.7	9	1.9%	0.80 [-0.17, 1.77]
Moller 2015 (b)	75	31.91	4	66.67	47.14	2	0.6%	0.18 [-1.52, 1.89]
Persoon 2017	86	20.3	50	83.7	20.4	47	11.3%	0.11 [-0.29, 0.51]
**Total (95% CI)**			**438**			**426**	**100.0%**	**0.07 [-0.07, 0.20]**

Heterogeneity: Tau² = 0.00; Chi² = 6.06, df = 8 (P = 0.64); I² = 0%
Test for overall effect: Z = 0.97 (P = 0.33)

Std. Mean Difference IV, Random, 95% CI

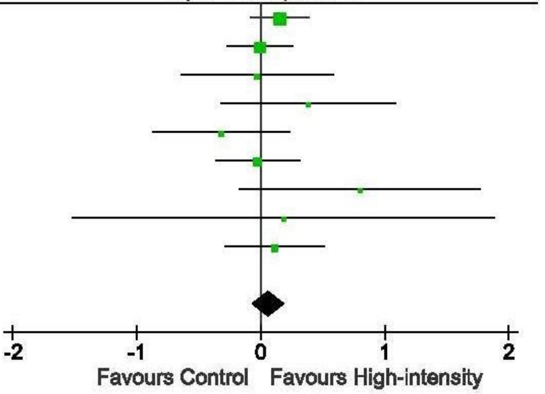

Supplementary Figure S47 Effects of the high-intensity training part duration in social functioning dimension.

Social functioning dimension: 15 minutes or less of high-intensity training

Study or Subgroup	High-intensity group Mean	SD	Total	Control group Mean	SD	Total	Weight	Std. Mean Difference IV, Random, 95% CI
Adamsen 2009	82.6	20.5	135	79.4	20.8	134	41.4%	0.15 [-0.08, 0.39]
Andersen 2013	23.25	3.84	106	23.27	3.73	107	32.9%	-0.01 [-0.27, 0.26]
Egegaard 2019	23.3	5.8	8	24.2	3.8	5	1.9%	-0.16 [-1.28, 0.96]
Mijwel 2017	62.32	46.87	74	63.3	27.67	60	20.5%	-0.02 [-0.37, 0.32]
Moller 2015 (a)	87.04	16.2	9	74.07	14.7	9	2.5%	0.80 [-0.17, 1.77]
Moller 2015 (b)	75	31.91	4	66.67	47.14	2	0.8%	0.18 [-1.52, 1.89]
**Total (95% CI)**			**336**			**317**	**100.0%**	**0.08 [-0.08, 0.23]**

Heterogeneity: Tau² = 0.00; Chi² = 3.42, df = 5 (P = 0.64); I² = 0%
Test for overall effect: Z = 0.96 (P = 0.33)

Std. Mean Difference IV, Random, 95% CI

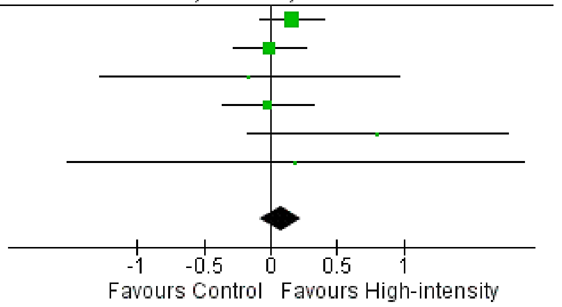

Social functioning dimension: More than 15 minutes of high-intensity training

Study or Subgroup	High-intensity group Mean	SD	Total	Control group Mean	SD	Total	Weight	Std. Mean Difference IV, Random, 95% CI
Adams 2018	45	5.1	35	42.6	7.4	27	9.4%	0.38 [-0.12, 0.89]
Cormie 2013	48.2	8.4	22	48.5	12.1	19	6.4%	-0.03 [-0.64, 0.59]
Hwang 2012	91	12.9	13	87.8	21.8	11	3.7%	0.18 [-0.63, 0.98]
Kampshoff 2015	89.6	15.1	91	85.2	21.7	91	28.5%	0.23 [-0.06, 0.53]
Martin 2015 (a)	24	3.5	12	22.1	5.4	23	4.9%	0.38 [-0.32, 1.09]
Martin 2015 (c)	20.2	3.6	26	21.5	4.4	25	7.9%	-0.32 [-0.87, 0.23]
Persoon 2017	86	20.3	50	83.7	20.4	47	15.2%	0.11 [-0.29, 0.51]
Van Wart 2015	73.5	21.6	76	67.9	29.1	77	23.9%	0.22 [-0.10, 0.54]
**Total (95% CI)**			**325**			**320**	**100.0%**	**0.17 [0.01, 0.33]**

Heterogeneity: Tau² = 0.00; Chi² = 4.79, df = 7 (P = 0.69); I² = 0%
Test for overall effect: Z = 2.14 (P = 0.03)

Std. Mean Difference IV, Random, 95% CI

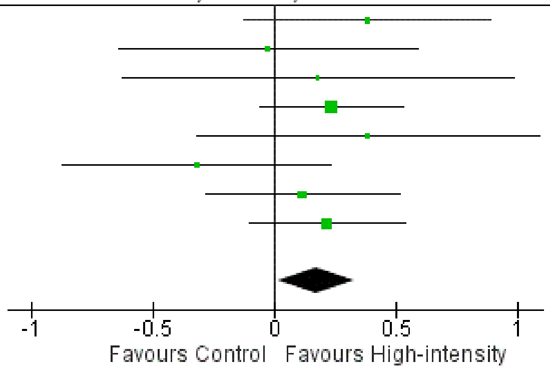

Supplementary Figure S48: Effects in the fatigue dimension of the comparisons between high-intensity training group and control group, and high- intensity training group and low- intensity exercise group.

Fatigue dimension: Comparison between high-intensity group and control group

Study or Subgroup	High-intensity group Mean	SD	Total	Control group Mean	SD	Total	Weight	Std. Mean Difference IV, Random, 95% CI
Adams 2018	-55	7.8	35	-50.8	8.9	28	8.7%	-0.50 [-1.00, 0.01]
Adamsen 2009	34.6	24.3	135	41	22.7	134	38.4%	-0.27 [-0.51, -0.03]
Cormie 2013	-48.6	9.4	22	-46.2	13.9	19	5.8%	-0.20 [-0.82, 0.41]
Hwang 2012	9.4	11.9	13	17.2	18.2	11	3.3%	-0.50 [-1.32, 0.32]
Mijwel 2017	37.18	23.7	74	48.49	24.88	60	18.6%	-0.46 [-0.81, -0.12]
Moller 2015 (a)	39.51	28.93	9	50.62	28.39	9	2.5%	-0.37 [-1.30, 0.56]
Moller 2015 (b)	41.67	31.91	4	66.67	31.43	2	0.7%	-0.63 [-2.43, 1.17]
Van Wart 2015	46	23.7	76	51.3	23.7	77	21.9%	-0.22 [-0.54, 0.10]
**Total (95% CI)**			**368**			**340**	**100.0%**	**-0.32 [-0.47, -0.18]**

Heterogeneity: Tau² = 0.00; Chi² = 2.12, df = 7 (P = 0.95); I² = 0%
Test for overall effect: Z = 4.28 (P < 0.0001)

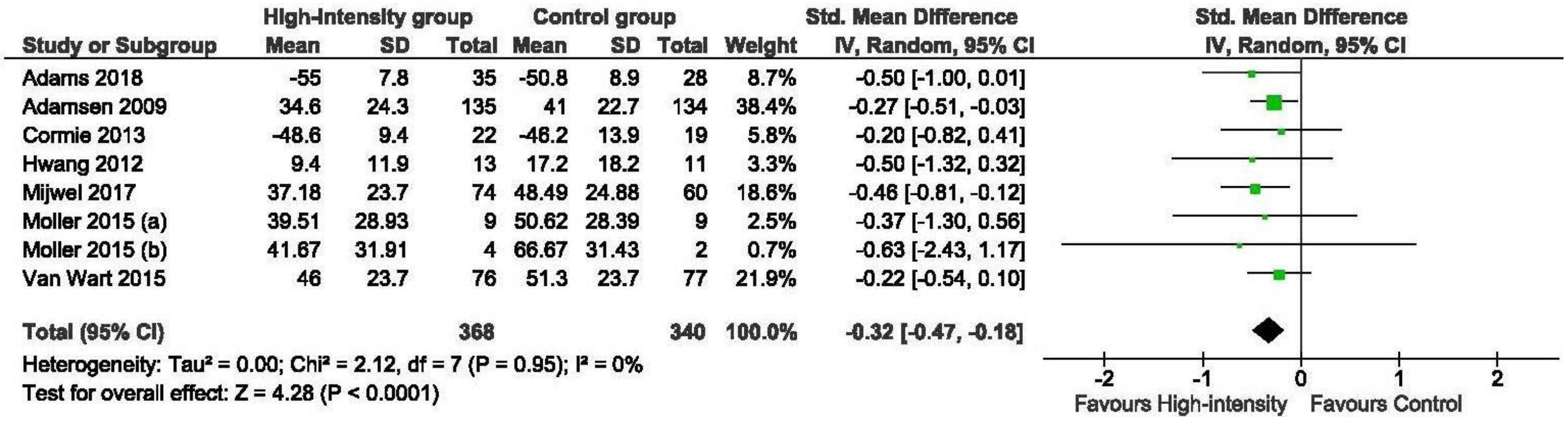

Fatigue dimension: Comparison between high-intensity group and low-moderate intensity group

Study or Subgroup	High-intensity group Mean	SD	Total	Active group Mean	SD	Total	Weight	Std. Mean Difference IV, Random, 95% CI
Cormie 2013	-48.6	9.4	22	-54.3	7.3	21	24.4%	0.66 [0.05, 1.28]
Moller 2015 (a)	39.51	28.93	9	50.62	28.39	72	23.0%	-0.39 [-1.08, 0.31]
Moller 2015 (b)	41.67	31.91	4	66.67	31.43	9	15.0%	-0.74 [-1.96, 0.49]
Schmitt 2016	27.1	13.9	13	51.85	16.97	3	12.8%	-1.63 [-3.04, -0.21]
Van Wart 2015	46	23.7	76	38.3	21.7	13	24.8%	0.33 [-0.26, 0.92]
**Total (95% CI)**			**124**			**118**	**100.0%**	**-0.17 [-0.83, 0.50]**

Heterogeneity: Tau² = 0.37; Chi² = 13.14, df = 4 (P = 0.01); I² = 70%
Test for overall effect: Z = 0.49 (P = 0.62)

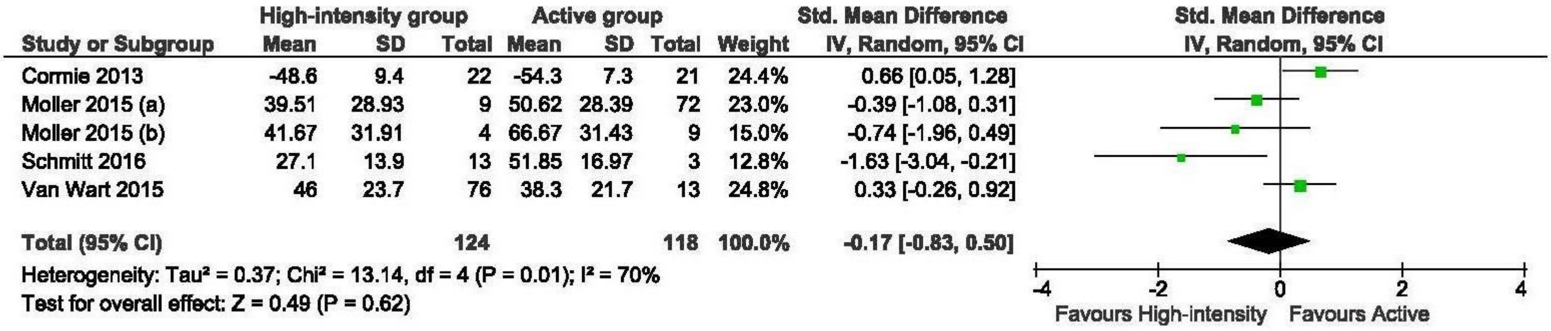

Supplementary Figure S49: Effects of high-intensity training in fatigue according to the treatment timing.

Fatigue functioning dimension: During treatment

Study or Subgroup	High-intensity group Mean	SD	Total	Control group Mean	SD	Total	Weight	Std. Mean Difference IV, Random, 95% CI
Adamsen 2009	34.6	24.3	135	41	22.7	134	85.5%	-0.27 [-0.51, -0.03]
Hwang 2012	9.4	11.9	13	17.2	18.2	11	7.4%	-0.50 [-1.32, 0.32]
Moller 2015 (a)	39.51	28.93	9	50.62	28.39	9	5.6%	-0.37 [-1.30, 0.56]
Moller 2015 (b)	41.67	31.91	4	66.67	31.43	2	1.5%	-0.63 [-2.43, 1.17]
**Total (95% CI)**			**161**			**156**	**100.0%**	**-0.30 [-0.52, -0.08]**

Heterogeneity: Tau² = 0.00; Chi² = 0.43, df = 3 (P = 0.93); I² = 0%
Test for overall effect: Z = 2.64 (P = 0.008)

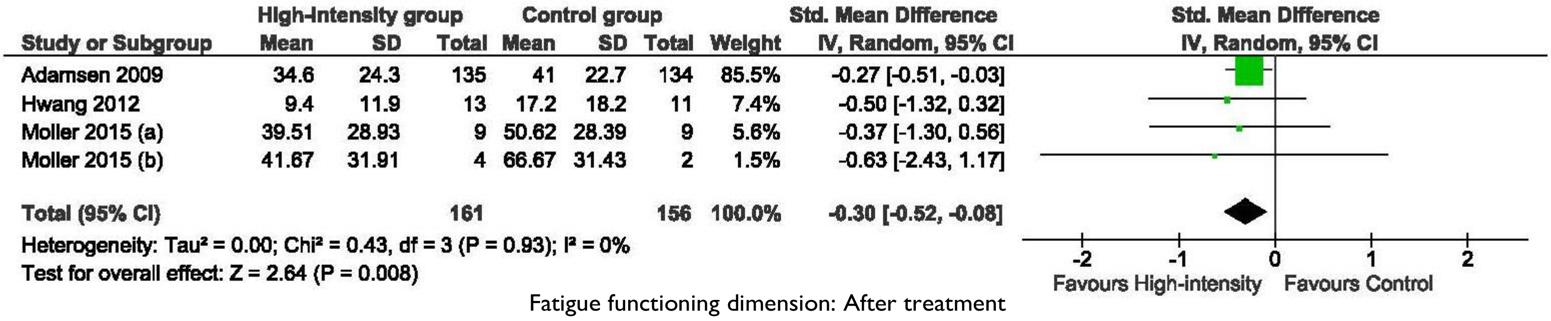

Fatigue functioning dimension: After treatment

Study or Subgroup	High-intensity group Mean	SD	Total	Control group Mean	SD	Total	Weight	Std. Mean Difference IV, Random, 95% CI
Adams 2018	-55	7.8	35	-50.8	8.9	28	26.2%	-0.50 [-1.00, 0.01]
Cormie 2013	-48.6	9.4	22	-46.2	13.9	19	17.6%	-0.20 [-0.82, 0.41]
Mijwel 2017	37.18	23.7	74	48.49	24.88	60	56.1%	-0.46 [-0.81, -0.12]
**Total (95% CI)**			**131**			**107**	**100.0%**	**-0.43 [-0.69, -0.17]**

Heterogeneity: Tau² = 0.00; Chi² = 0.64, df = 2 (P = 0.73); I² = 0%
Test for overall effect: Z = 3.24 (P = 0.001)

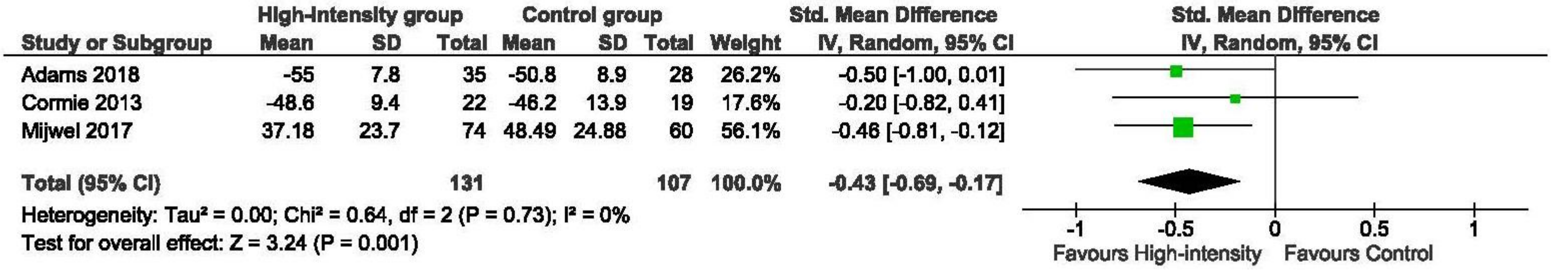

Supplementary Figure S50: Effects of the weekly exercise frequency in fatigue dimension.

Fatigue functioning dimension: 2 times per week

Study or Subgroup	High-intensity group Mean	SD	Total	Control group Mean	SD	Total	Weight	Std. Mean Difference IV, Random, 95% CI
Cormie 2013	-48.6	9.4	22	-46.2	13.9	19	12.6%	-0.20 [-0.82, 0.41]
Mijwel 2017	37.18	23.7	74	48.49	24.88	60	40.1%	-0.46 [-0.81, -0.12]
Van Wart 2015	46	23.7	76	51.3	23.7	77	47.3%	-0.22 [-0.54, 0.10]
**Total (95% CI)**			**172**			**156**	**100.0%**	**-0.32 [-0.54, -0.10]**

Heterogeneity: Tau² = 0.00; Chi² = 1.17, df = 2 (P = 0.56); I² = 0%
Test for overall effect: Z = 2.84 (P = 0.005)

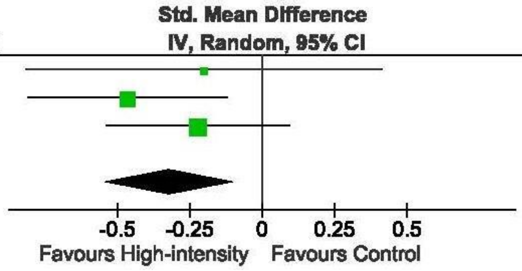

Fatigue functioning dimension: 3 times per week

Study or Subgroup	High-intensity group Mean	SD	Total	Control group Mean	SD	Total	Weight	Std. Mean Difference IV, Random, 95% CI
Adams 2018	-55	7.8	35	-50.8	8.9	28	16.2%	-0.50 [-1.00, 0.01]
Adamsen 2009	34.6	24.3	135	41	22.7	134	71.6%	-0.27 [-0.51, -0.03]
Hwang 2012	9.4	11.9	13	17.2	18.2	11	6.2%	-0.50 [-1.32, 0.32]
Moller 2015 (a)	39.51	28.93	9	50.62	28.39	9	4.7%	-0.37 [-1.30, 0.56]
Moller 2015 (b)	41.67	31.91	4	66.67	31.43	2	1.3%	-0.63 [-2.43, 1.17]
**Total (95% CI)**			**196**			**184**	**100.0%**	**-0.33 [-0.53, -0.13]**

Heterogeneity: Tau² = 0.00; Chi² = 0.94, df = 4 (P = 0.92); I² = 0%
Test for overall effect: Z = 3.20 (P = 0.001)

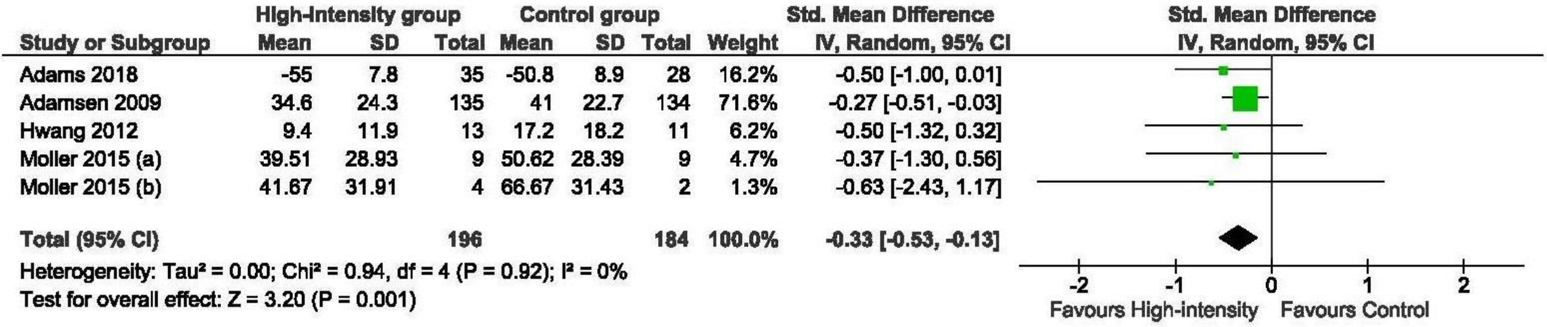

Supplementary Figure S51: Effects of training minutes per week in fatigue dimension

Fatigue dimension: Less than 120 minutes of exercise per week

Study or Subgroup	High-intensity group Mean	SD	Total	Control group Mean	SD	Total	Weight	Std. Mean Difference IV, Random, 95% CI
Adams 2018	-55	7.8	35	-50.8	8.9	28	25.6%	-0.50 [-1.00, 0.01]
Hwang 2012	9.4	11.9	13	17.2	18.2	11	9.8%	-0.50 [-1.32, 0.32]
Van Wart 2015	46	23.7	76	51.3	23.7	77	64.6%	-0.22 [-0.54, 0.10]
**Total (95% CI)**			**124**			**116**	**100.0%**	**-0.32 [-0.58, -0.06]**

Heterogeneity: Tau² = 0.00; Chi² = 1.03, df = 2 (P = 0.60); I² = 0%
Test for overall effect: Z = 2.46 (P = 0.01)

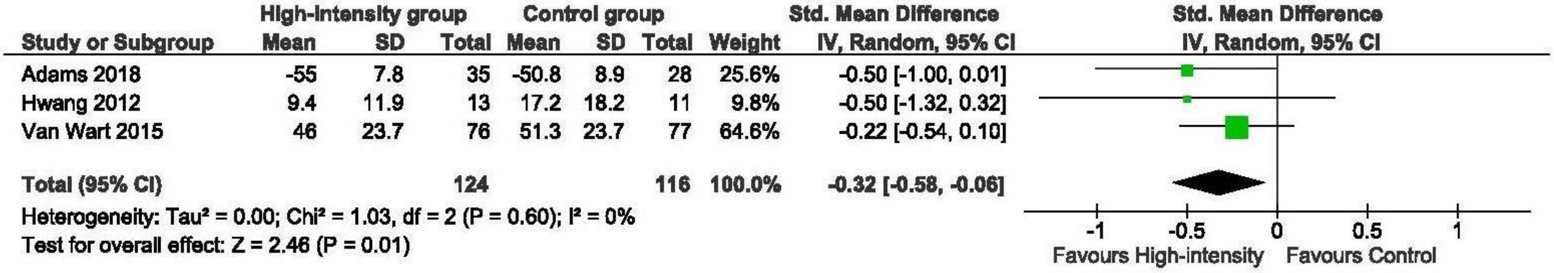

Fatigue dimension:120 minutes or more of exercise per week

Study or Subgroup	High-intensity group Mean	SD	Total	Control group Mean	SD	Total	Weight	Std. Mean Difference IV, Random, 95% CI
Adamsen 2009	34.6	24.3	135	41	22.7	134	58.1%	-0.27 [-0.51, -0.03]
Cormie 2013	-48.6	9.4	22	-46.2	13.9	19	8.8%	-0.20 [-0.82, 0.41]
Mijwel 2017	37.18	23.7	74	48.49	24.88	60	28.1%	-0.46 [-0.81, -0.12]
Moller 2015 (a)	39.51	28.93	9	50.62	28.39	9	3.8%	-0.37 [-1.30, 0.56]
Moller 2015 (b)	41.67	31.91	4	66.67	31.43	2	1.0%	-0.63 [-2.43, 1.17]
**Total (95% CI)**			**244**			**224**	**100.0%**	**-0.33 [-0.51, -0.14]**

Heterogeneity: Tau² = 0.00; Chi² = 1.09, df = 4 (P = 0.90); I² = 0%
Test for overall effect: Z = 3.50 (P = 0.0005)

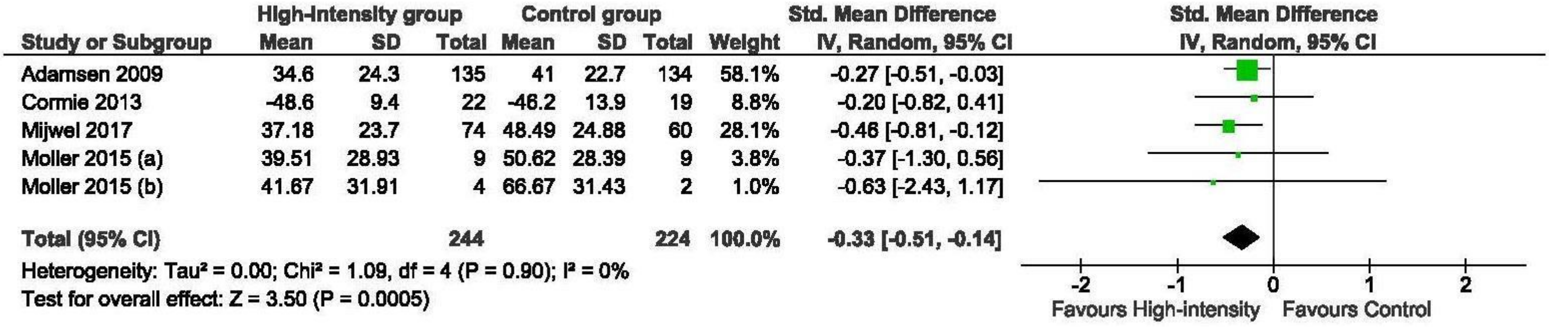

Supplementary Figure S52: Effects of the high-intensity training part duration in fatigue dimension

Fatigue dimension:15 minutes or les of high-intensity training

Study or Subgroup	High-intensity group Mean	SD	Total	Control group Mean	SD	Total	Weight	Std. Mean Difference IV, Random, 95% CI
Adamsen 2009	34.6	24.3	135	41	22.7	134	63.8%	-0.27 [-0.51, -0.03]
Mijwel 2017	37.18	23.7	74	48.49	24.88	60	30.9%	-0.46 [-0.81, -0.12]
Moller 2015 (a)	39.51	28.93	9	50.62	28.39	9	4.2%	-0.37 [-1.30, 0.56]
Moller 2015 (b)	41.67	31.91	4	66.67	31.43	2	1.1%	-0.63 [-2.43, 1.17]
**Total (95% CI)**			**222**			**205**	**100.0%**	**-0.34 [-0.53, -0.15]**

Heterogeneity: Tau² = 0.00; Chi² = 0.91, df = 3 (P = 0.82); I² = 0%
Test for overall effect: Z = 3.47 (P = 0.0005)

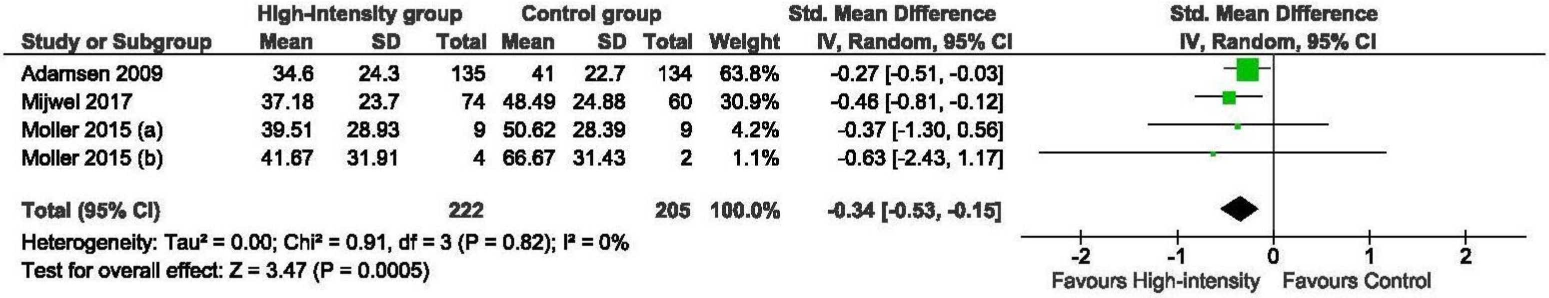

Fatigue dimension: More than 15 minutes of high-intensity training

Study or Subgroup	High-intensity group Mean	SD	Total	Control group Mean	SD	Total	Weight	Std. Mean Difference IV, Random, 95% CI
Adams 2018	-55	7.8	35	-50.8	8.9	28	21.8%	-0.50 [-1.00, 0.01]
Cormie 2013	-48.6	9.4	22	-46.2	13.9	19	14.7%	-0.20 [-0.82, 0.41]
Hwang 2012	9.4	11.9	13	17.2	18.2	11	8.3%	-0.50 [-1.32, 0.32]
Van Wart 2015	46	23.7	76	51.3	23.7	77	55.1%	-0.22 [-0.54, 0.10]
**Total (95% CI)**			**146**			**135**	**100.0%**	**-0.30 [-0.54, -0.07]**

Heterogeneity: Tau² = 0.00; Chi² = 1.15, df = 3 (P = 0.76); I² = 0%
Test for overall effect: Z = 2.52 (P = 0.01)

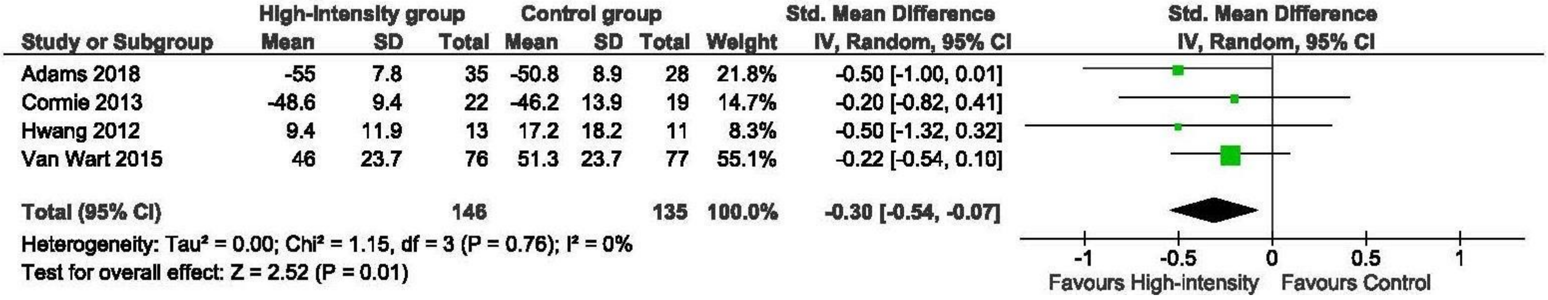

Supplementary Figure S53: Effects in the bodily pain dimension of the comparisons between high-intensity training group and control group, and high- intensity training group and low- intensity exercise group.

Bodily pain dimension: Comparison between high-intensity group and control group

Study or Subgroup	High-intensity group Mean	SD	Total	Control group Mean	SD	Total	Weight	Std. Mean Difference IV, Random, 95% CI
Adams 2018	-52.5	7.3	35	-52.8	7.9	27	9.5%	0.04 [-0.46, 0.54]
Adamsen 2009	14.6	17.1	135	16.8	20.6	134	38.2%	-0.12 [-0.36, 0.12]
Cormie 2013	-49.9	9.1	22	-52.8	9.6	19	6.3%	0.30 [-0.31, 0.92]
Mijwel 2017	21.01	24.06	74	27.95	30.2	60	19.9%	-0.26 [-0.60, 0.09]
Moller 2015 (a)	20.37	18.22	9	25.93	22.22	9	2.8%	-0.26 [-1.19, 0.67]
Moller 2015 (b)	33.33	45.13	4	50	70.71	2	0.8%	-0.25 [-1.97, 1.46]
Van Wart 2015	22.3	20.1	76	31.8	22.2	77	22.4%	-0.45 [-0.77, -0.13]
**Total (95% CI)**			**355**			**328**	**100.0%**	**-0.18 [-0.34, -0.02]**

Heterogeneity: Tau² = 0.00; Chi² = 6.24, df = 6 (P = 0.40); I² = 4%
Test for overall effect: Z = 2.26 (P = 0.02)

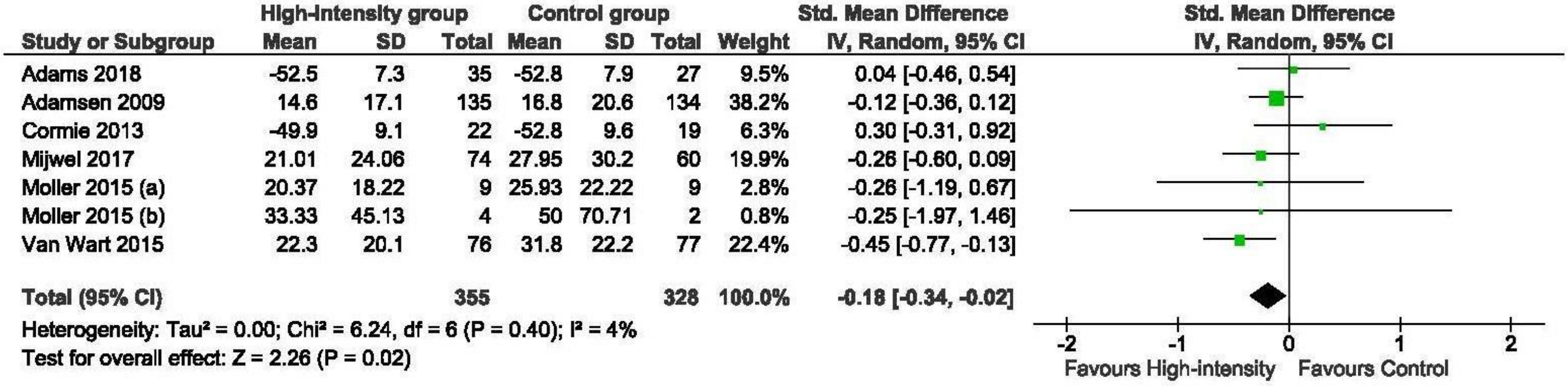

Bodily pain dimension: Comparison between high-intensity group and low-moderate intensity group

Study or Subgroup	High-intensity group Mean	SD	Total	Active group Mean	SD	Total	Weight	Std. Mean Difference IV, Random, 95% CI
Cormie 2013	-49.9	9.1	22	-51.7	8.7	21	17.5%	0.20 [-0.40, 0.80]
Moller 2015 (a)	20.37	18.22	9	20.37	18.22	9	7.4%	0.00 [-0.92, 0.92]
Moller 2015 (b)	33.33	45.13	4	22.22	19.25	3	2.8%	0.25 [-1.26, 1.76]
Schmitt 2016	19.2	20.2	13	32	18.6	13	10.0%	-0.64 [-1.43, 0.15]
Van Wart 2015	22.3	20.1	76	19.9	24.8	77	62.4%	0.11 [-0.21, 0.42]
**Total (95% CI)**			**124**			**123**	**100.0%**	**0.04 [-0.21, 0.29]**

Heterogeneity: Tau² = 0.00; Chi² = 3.34, df = 4 (P = 0.50); I² = 0%
Test for overall effect: Z = 0.34 (P = 0.73)

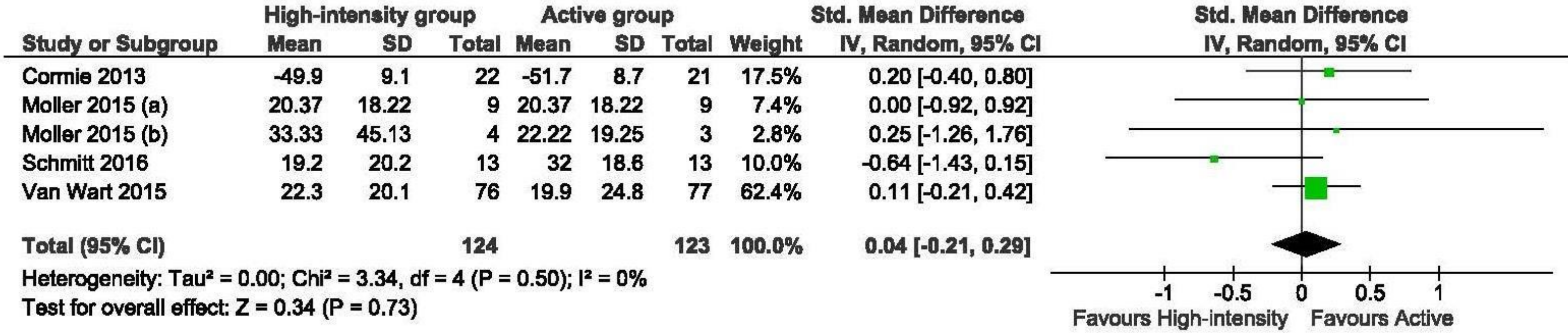

Supplementary Figure S54: Effects of the exercise programs length in bodily pain dimension

Bodily pain dimension: Eight weeks of exercise program or less

Study or Subgroup	High-intensity group Mean	SD	Total	Control group Mean	SD	Total	Weight	Std. Mean Difference IV, Random, 95% CI
Adamsen 2009	14.6	17.1	135	16.8	20.6	134	92.1%	-0.12 [-0.36, 0.12]
Moller 2015 (a)	20.37	18.22	9	25.93	22.22	9	6.1%	-0.26 [-1.19, 0.67]
Moller 2015 (b)	33.33	45.13	4	50	70.71	2	1.8%	-0.25 [-1.97, 1.46]
**Total (95% CI)**			**148**			**145**	**100.0%**	**-0.13 [-0.36, 0.10]**

Heterogeneity: Tau² = 0.00; Chi² = 0.11, df = 2 (P = 0.95); I² = 0%
Test for overall effect: Z = 1.09 (P = 0.28)

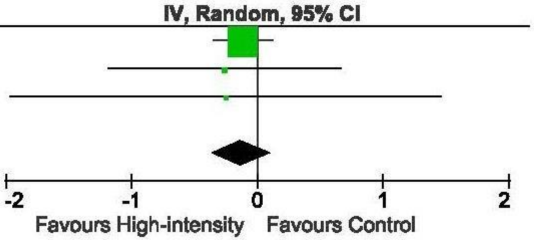

Bodily pain dimension: More than eight weeks of exercise program

Study or Subgroup	High- intensity group Mean	SD	Total	Control group Mean	SD	Total	Weight	Std. Mean Difference IV, Random, 95% CI
Adams 2018	-52.5	7.3	35	-52.8	7.9	27	29.4%	0.04 [-0.46, 0.54]
Cormie 2013	-49.9	9.1	22	-52.8	9.6	19	21.2%	0.30 [-0.31, 0.92]
Mijwel 2017	21.01	24.06	74	27.95	30.2	60	49.4%	-0.26 [-0.60, 0.09]
**Total (95% CI)**			**131**			**106**	**100.0%**	**-0.05 [-0.36, 0.26]**

Heterogeneity: Tau² = 0.02; Chi² = 2.72, df = 2 (P = 0.26); I² = 27%
Test for overall effect: Z = 0.31 (P = 0.75)

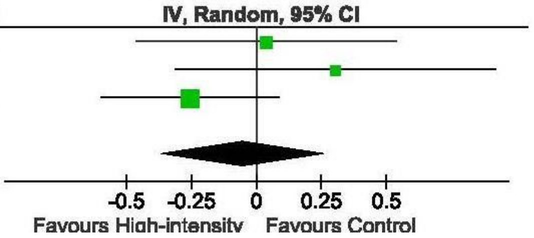

Supplementary Figure S55: Effects of the weekly exercise frequency in bodily pain dimension.

Bodily pain dimension: 2 times per week

Study or Subgroup	High-intensity group Mean	SD	Total	Control group Mean	SD	Total	Weight	Std. Mean Difference IV, Random, 95% CI
Cormie 2013	-49.9	9.1	22	-52.8	9.6	19	21.0%	0.30 [-0.31, 0.92]
Mijwel 2017	21.01	24.06	74	27.95	30.2	60	38.6%	-0.26 [-0.60, 0.09]
Van Wart 2015	22.3	20.1	76	31.8	22.2	77	40.4%	-0.45 [-0.77, -0.13]
**Total (95% CI)**			**172**			**156**	**100.0%**	**-0.22 [-0.56, 0.13]**

Heterogeneity: Tau² = 0.05; Chi² = 4.49, df = 2 (P = 0.11); I² = 55%
Test for overall effect: Z = 1.21 (P = 0.23)

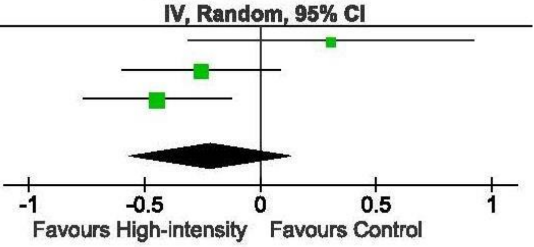

Bodily pain dimension: 3 times per week

Study or Subgroup	High-intensity group Mean	SD	Total	Control group Mean	SD	Total	Weight	Std. Mean Difference IV, Random, 95% CI
Adams 2018	-52.5	7.3	35	-52.8	7.9	27	17.3%	0.04 [-0.46, 0.54]
Adamsen 2009	14.6	17.1	135	16.8	20.6	134	76.2%	-0.12 [-0.36, 0.12]
Moller 2015 (a)	20.37	18.22	9	25.93	22.22	9	5.1%	-0.26 [-1.19, 0.67]
Moller 2015 (b)	33.33	45.13	4	50	70.71	2	1.5%	-0.25 [-1.97, 1.46]
**Total (95% CI)**			**183**			**172**	**100.0%**	**-0.10 [-0.31, 0.11]**

Heterogeneity: Tau² = 0.00; Chi² = 0.46, df = 3 (P = 0.93); I² = 0%
Test for overall effect: Z = 0.92 (P = 0.36)

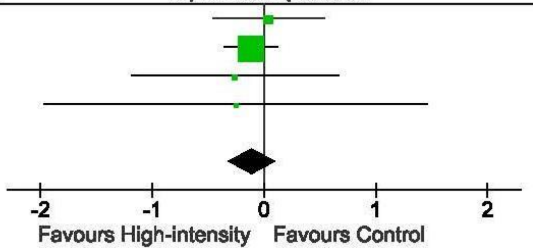

Supplementary Figure S56: Effects of the high-intensity training part duration in bodily pain dimension.

Bodily pain dimension: 15 minutes or les of high-intensity training

Study or Subgroup	High-Intensity group Mean	SD	Total	Control group Mean	SD	Total	Weight	Std. Mean Difference IV, Random, 95% CI
Adamsen 2009	14.6	17.1	135	16.8	20.6	134	63.5%	-0.12 [-0.36, 0.12]
Mijwel 2017	21.01	24.06	74	27.95	30.2	60	31.1%	-0.26 [-0.60, 0.09]
Moller 2015 (a)	20.37	18.22	9	25.93	22.22	9	4.2%	-0.26 [-1.19, 0.67]
Moller 2015 (b)	33.33	45.13	4	50	70.71	2	1.2%	-0.25 [-1.97, 1.46]
**Total (95% CI)**			**222**			**205**	**100.0%**	**-0.17 [-0.36, 0.02]**

Heterogeneity: Tau² = 0.00; Chi² = 0.48, df = 3 (P = 0.92); I² = 0%
Test for overall effect: Z = 1.72 (P = 0.09)

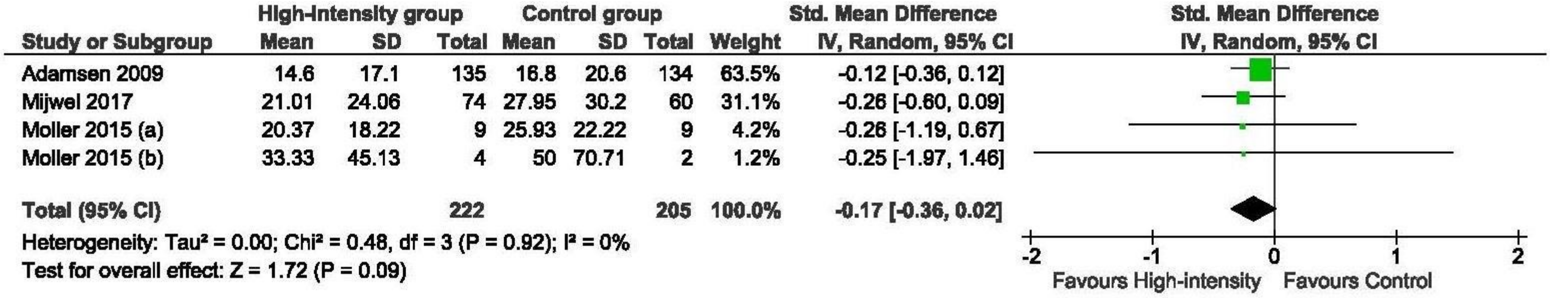

Bodily pain dimension: More than 15 minutes of high-intensity training

Study or Subgroup	High-Intensity group Mean	SD	Total	Control group Mean	SD	Total	Weight	Std. Mean Difference IV, Random, 95% CI
Adams 2018	-52.5	7.3	35	-52.8	7.9	27	32.0%	0.04 [-0.46, 0.54]
Cormie 2013	-49.9	9.1	22	-52.8	9.6	19	26.7%	0.30 [-0.31, 0.92]
Van Wart 2015	22.3	20.1	76	31.8	22.2	77	41.3%	-0.45 [-0.77, -0.13]
**Total (95% CI)**			**133**			**123**	**100.0%**	**-0.09 [-0.55, 0.37]**

Heterogeneity: Tau² = 0.11; Chi² = 5.69, df = 2 (P = 0.06); I² = 65%
Test for overall effect: Z = 0.39 (P = 0.70)

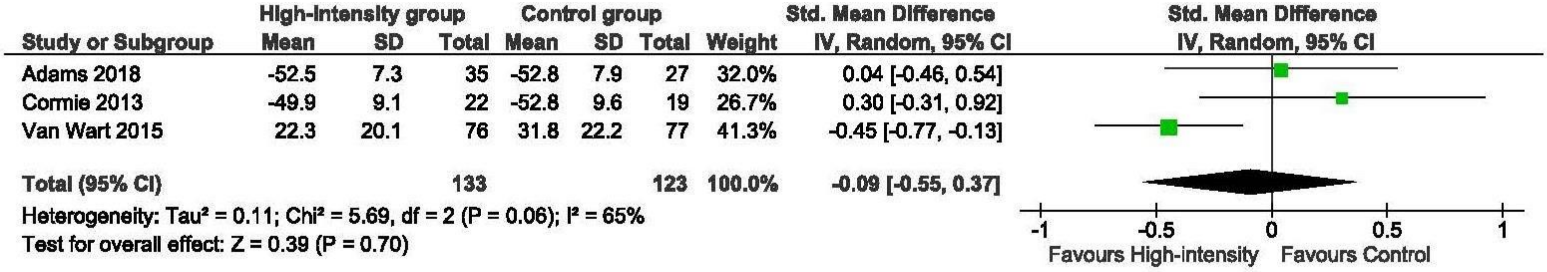

Supplementary Figure S57: Effects in the dysnoea dimension of the comparisons between high-intensity training group and control group, and high- intensity training group and low- intensity exercise group.

Dyspnoea dimension: Comparison between high-intensity group and control group

Study or Subgroup	High-Intensity group Mean	SD	Total	Control group Mean	SD	Total	Weight	Std. Mean Difference IV, Random, 95% CI
Adamsen 2009	14.3	21.5	135	18.2	24.4	138	45.6%	-0.17 [-0.41, 0.07]
Edvardsen 2015	37	25.3	30	58	32.1	31	14.3%	-0.72 [-1.23, -0.20]
Hwang 2012	3.8	5.5	13	13.6	14.6	11	5.8%	-0.89 [-1.74, -0.04]
Mijwel 2017	35.17	28.83	74	43.49	28.97	60	28.1%	-0.29 [-0.63, 0.06]
Moller 2015 (a)	11.11	16.67	9	18.52	24.22	9	4.9%	-0.34 [-1.27, 0.59]
Moller 2015 (b)	8.33	16.67	4	50	70.71	2	1.2%	-0.87 [-2.77, 1.02]
**Total (95% CI)**			**265**			**251**	**100.0%**	**-0.34 [-0.55, -0.13]**

Heterogeneity: Tau² = 0.01; Chi² = 5.84, df = 5 (P = 0.32); I² = 14%
Test for overall effect: Z = 3.15 (P = 0.002)

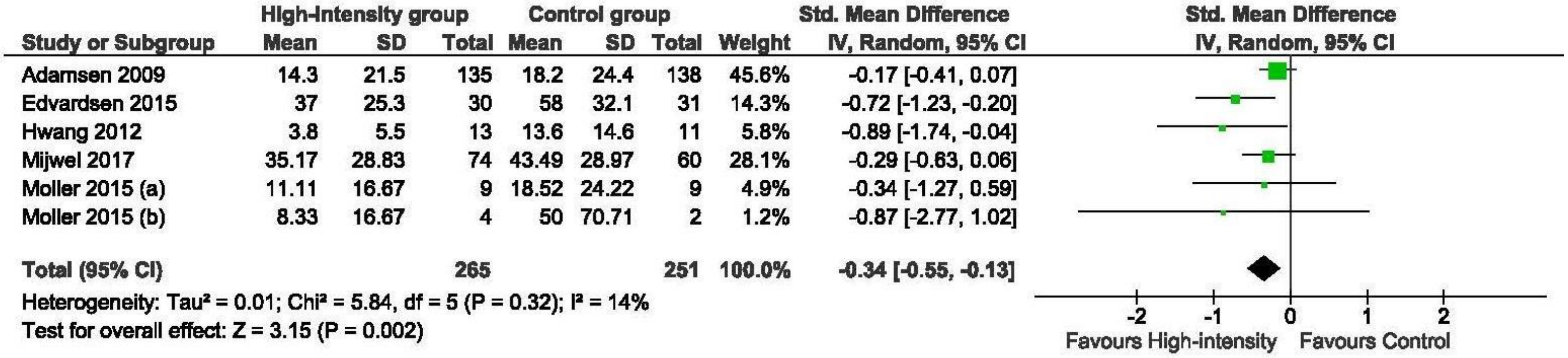

Dyspnoea dimension: Comparison between high-intensity group and low-moderate intensity group

Study or Subgroup	High-intensity group Mean	SD	Total	Active group Mean	SD	Total	Weight	Std. Mean Difference IV, Random, 95% CI
Moller 2015 (a)	11.11	16.67	9	18.52	24.22	9	35.8%	-0.34 [-1.27, 0.59]
Moller 2015 (b)	8.33	16.67	4	11.11	19.35	3	13.8%	-0.13 [-1.63, 1.37]
Schmitt 2016	20.3	16.7	13	32.2	23.7	13	50.3%	-0.56 [-1.35, 0.22]
**Total (95% CI)**			**26**			**25**	**100.0%**	**-0.42 [-0.98, 0.14]**

Heterogeneity: Tau² = 0.00; Chi² = 0.30, df = 2 (P = 0.86); I² = 0%
Test for overall effect: Z = 1.49 (P = 0.14)

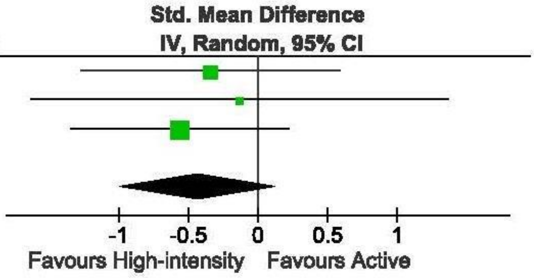

Supplementary Figure S58: Effects in the diarrhoea dimension of the comparisons between high-intensity training group and control group, and high- intensity training group and low- intensity exercise group.

Diarrhoea dimension: Comparison between high-intensity group and control group

Study or Subgroup	High-intensity group Mean	SD	Total	Control group Mean	SD	Total	Weight	Std. Mean Difference IV, Random, 95% CI
Adamsen 2009	17	26.1	135	13	21.9	134	63.5%	0.17 [-0.07, 0.40]
Mijwel 2017	8.9	18.88	74	8.47	16.55	60	31.4%	0.02 [-0.32, 0.36]
Moller 2015 (a)	22.22	28.87	9	3.7	11.11	9	3.9%	0.81 [-0.16, 1.78]
Moller 2015 (b)	16.67	19.25	4	33.33	47.14	2	1.2%	-0.46 [-2.22, 1.29]
**Total (95% CI)**			**222**			**205**	**100.0%**	**0.14 [-0.05, 0.33]**

Heterogeneity: Tau² = 0.00; Chi² = 2.75, df = 3 (P = 0.43); I² = 0%
Test for overall effect: Z = 1.42 (P = 0.16)

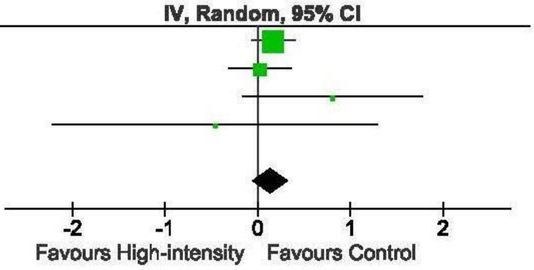

Diarrhoea dimension: Comparison between high-intensity group and low-moderate intensity group

Study or Subgroup	High-intensity group Mean	SD	Total	Active group Mean	SD	Total	Weight	Std. Mean Difference IV, Random, 95% CI
Mijwel 2017	8.9	18.88	74	12.39	19.93	72	67.6%	-0.18 [-0.50, 0.15]
Moller 2015 (a)	22.22	28.87	9	7.41	14.7	9	11.3%	0.62 [-0.34, 1.57]
Moller 2015 (b)	16.67	19.25	4	11.11	19.25	3	4.7%	0.24 [-1.27, 1.75]
Schmitt 2016	5.1	12.4	13	15.3	29.2	13	16.4%	-0.44 [-1.22, 0.34]
**Total (95% CI)**			**100**			**97**	**100.0%**	**-0.11 [-0.44, 0.22]**

Heterogeneity: Tau² = 0.01; Chi² = 3.29, df = 3 (P = 0.35); I² = 9%
Test for overall effect: Z = 0.67 (P = 0.51)

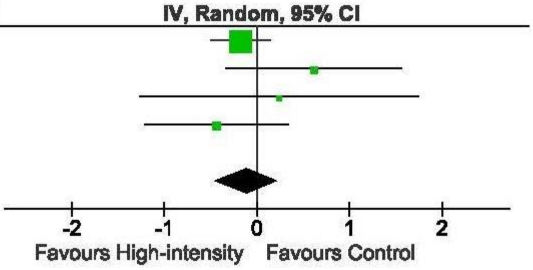

Supplementary Figure S59: Effects in the nausea dimension of the comparisons between high-intensity training group and control group.

Nausea dimension: Comparison between high-intensity group and control group.

Study or Subgroup	High-intensity group Mean	SD	Total	Control group Mean	SD	Total	Weight	Std. Mean Difference IV, Random, 95% CI
Adamsen 2009	13.4	17.3	135	13.7	18.4	134	40.5%	-0.02 [-0.26, 0.22]
Mijwel 2017	5.02	10.1	74	7.79	20.37	60	25.8%	-0.18 [-0.52, 0.16]
Moller 2015 (a)	7.41	12.11	9	18.52	13.03	9	4.2%	-0.84 [-1.82, 0.13]
Moller 2015 (b)	25	16.67	4	41.67	11.79	2	1.2%	-0.86 [-2.74, 1.03]
Van Wart 2015	4.2	9.6	76	10.4	22.8	77	28.3%	-0.35 [-0.67, -0.03]
**Total (95% CI)**			**298**			**282**	**100.0%**	**-0.20 [-0.40, 0.01]**

Heterogeneity: Tau² = 0.01; Chi² = 5.15, df = 4 (P = 0.27); I² = 22%
Test for overall effect: Z = 1.89 (P = 0.06)

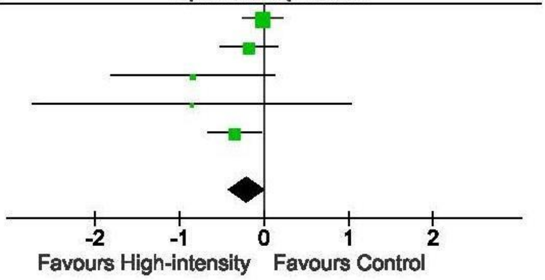

Supplementary Figure S60: Effects in the constipation dimension of the comparisons between high-intensity training group and control group.

Constipation dimension: Comparison between high-intensity group and control group.

Study or Subgroup	High-intensity group Mean	SD	Total	Control group Mean	SD	Total	Weight	Std. Mean Difference IV, Random, 95% CI
Adamsen 2009	20.8	39.4	118	18.4	25.3	117	30.7%	0.07 [-0.18, 0.33]
Mijwel 2017	10.14	21.79	74	14.47	23.76	60	27.4%	-0.19 [-0.53, 0.15]
Moller 2015 (a)	7.41	14.7	9	14.81	24.22	9	10.6%	-0.35 [-1.28, 0.58]
Moller 2015 (b)	25	16.67	4	33.33	47.14	2	4.0%	-0.24 [-1.95, 1.47]
Van Wart 2015	3.3	14	71	17.7	26.3	66	27.3%	-0.69 [-1.03, -0.34]
**Total (95% CI)**			**276**			**254**	**100.0%**	**-0.26 [-0.63, 0.10]**

Heterogeneity: Tau² = 0.09; Chi² = 12.10, df = 4 (P = 0.02); I² = 67%
Test for overall effect: Z = 1.43 (P = 0.15)

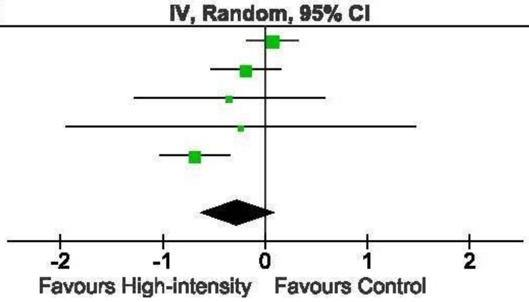

Supplementary Figure S61: Effects in the insomnia dimension of the comparisons between high-intensity training group and control group, and high- intensity training group and low- intensity exercise group.

Insomnia dimension: Comparison between high-intensity group and control group

Study or Subgroup	High-intensity group Mean	SD	Total	Active group Mean	SD	Total	Weight	Std. Mean Difference IV, Random, 95% CI
Adamsen 2009	18.4	23.9	135	26.4	27.5	134	61.2%	-0.31 [-0.55, -0.07]
Mijwel 2017	31.34	30.37	74	39.37	34.52	72	33.3%	-0.25 [-0.57, 0.08]
Moller 2015 (a)	18.52	24.22	9	37.04	42.31	9	4.0%	-0.51 [-1.45, 0.43]
Moller 2015 (b)	58.33	16.67	4	50	23.57	3	1.5%	0.36 [-1.17, 1.88]
**Total (95% CI)**			**222**			**218**	**100.0%**	**-0.29 [-0.47, -0.10]**

Heterogeneity: Tau² = 0.00; Chi² = 1.00, df = 3 (P = 0.80); I² = 0%
Test for overall effect: Z = 2.98 (P = 0.003)

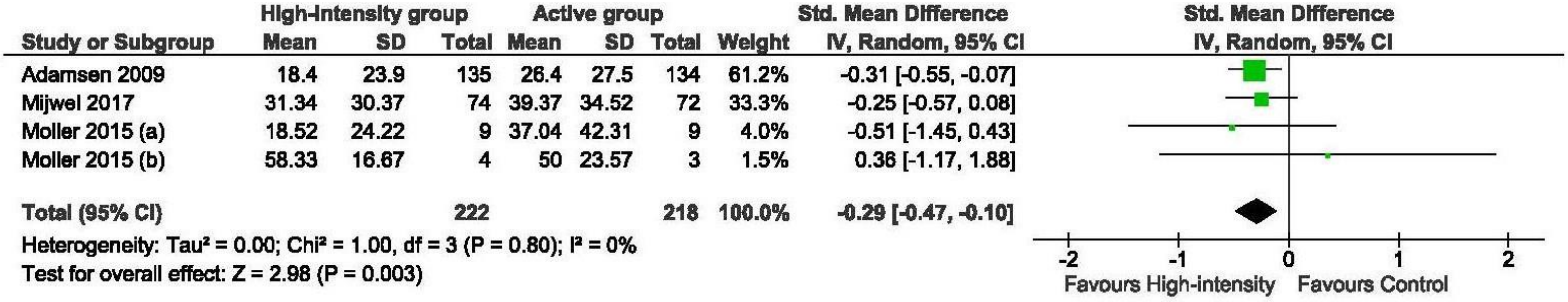

Insomnia dimension: Comparison between high-intensity group and low-moderate intensity group

Study or Subgroup	High-intensity group Mean	SD	Total	Active group Mean	SD	Total	Weight	Std. Mean Difference IV, Random, 95% CI
Mijwel 2017	31.34	30.37	74	39.37	34.52	72	74.4%	-0.25 [-0.57, 0.08]
Moller 2015 (a)	18.52	24.22	9	37.04	42.31	9	8.9%	-0.51 [-1.45, 0.43]
Moller 2015 (b)	58.33	16.67	4	50	23.57	3	3.4%	0.36 [-1.17, 1.88]
Schmitt 2016	38.5	26.9	13	43.5	25.2	13	13.3%	-0.19 [-0.96, 0.58]
**Total (95% CI)**			**100**			**97**	**100.0%**	**-0.24 [-0.52, 0.04]**

Heterogeneity: Tau² = 0.00; Chi² = 0.93, df = 3 (P = 0.82); I² = 0%
Test for overall effect: Z = 1.68 (P = 0.09)

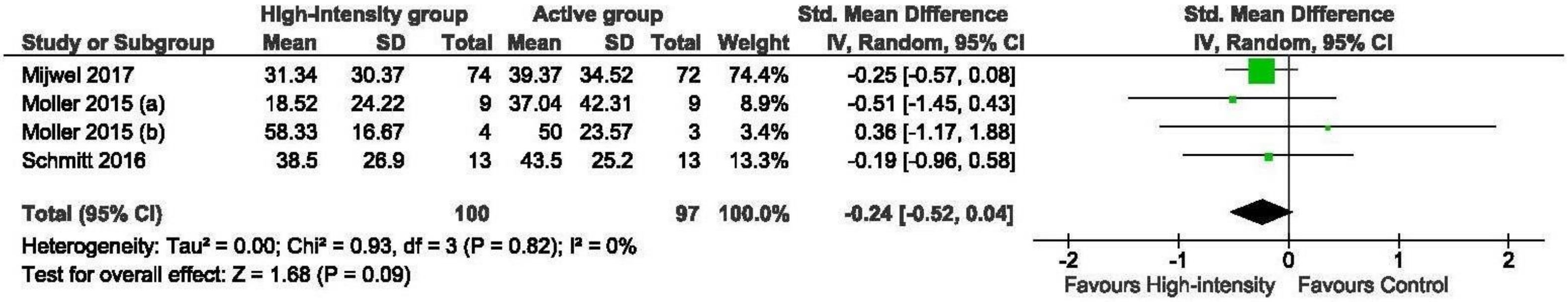

Supplementary Figure S62: Effects in the appetite loss dimension of the comparisons between high-intensity training group and control group, and high- intensity training group and low- intensity exercise group.

Appetite loss dimension: Comparison between high-intensity group and control group.

Study or Subgroup	High-intensity group Mean	SD	Total	Control group Mean	SD	Total	Weight	Std. Mean Difference IV, Random, 95% CI
Adamsen 2009	12	22.3	135	14.6	22.1	134	63.5%	-0.12 [-0.36, 0.12]
Mijwel 2017	13.33	23.62	74	18.83	26.66	60	31.1%	-0.22 [-0.56, 0.12]
Moller 2015 (a)	11.11	16.67	9	18.52	24.22	9	4.2%	-0.34 [-1.27, 0.59]
Moller 2015 (b)	16.67	33.33	4	33.33	33.33	2	1.2%	-0.40 [-2.14, 1.34]
**Total (95% CI)**			**222**			**205**	**100.0%**	**-0.16 [-0.35, 0.03]**

Heterogeneity: Tau² = 0.00; Chi² = 0.45, df = 3 (P = 0.93); I² = 0%
Test for overall effect: Z = 1.66 (P = 0.10)

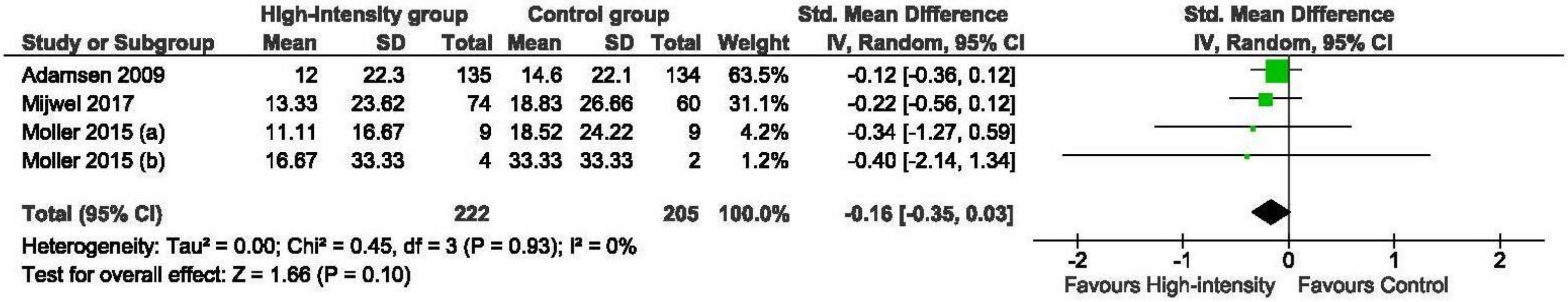

Appetite loss dimension: Comparison between high-intensity group and low-moderate intensity group

Study or Subgroup	High-intensity group Mean	SD	Total	Active group Mean	SD	Total	Weight	Std. Mean Difference IV, Random, 95% CI
Mijwel 2017	13.33	23.62	74	19.53	24.97	72	74.4%	-0.25 [-0.58, 0.07]
Moller 2015 (a)	11.11	16.67	9	14.81	33.79	9	9.2%	-0.13 [-1.06, 0.79]
Moller 2015 (b)	16.67	33.33	4	33.33	33.33	3	3.4%	-0.42 [-1.95, 1.11]
Schmitt 2016	2.5	9.2	13	7.6	14.5	13	13.0%	-0.41 [-1.18, 0.37]
**Total (95% CI)**			**100**			**97**	**100.0%**	**-0.27 [-0.55, 0.01]**

Heterogeneity: Tau² = 0.00; Chi² = 0.25, df = 3 (P = 0.97); I² = 0%
Test for overall effect: Z = 1.87 (P = 0.06)

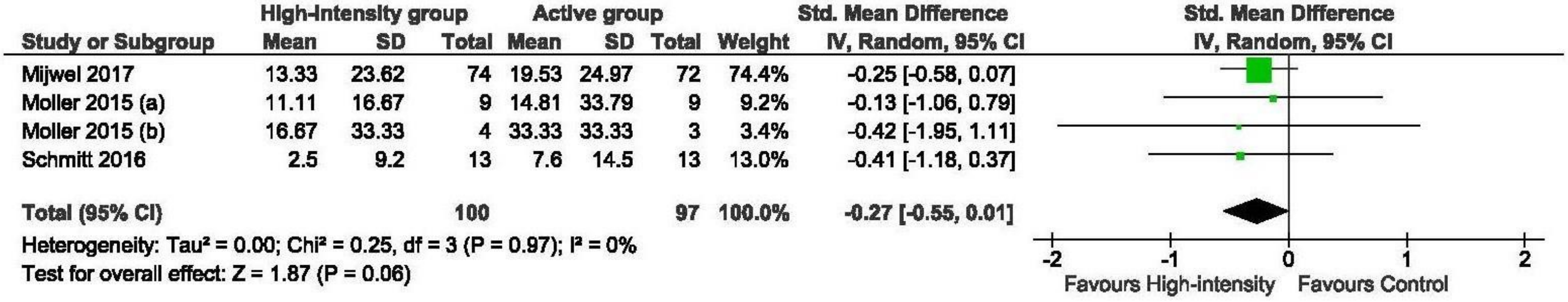

Supplement: Supplementary file 1 — Supplementary Information. [file 41598_2021_94476_MOESM1_ESM.pdf]
